# Global prevalence of liver disease in human and domestic animals caused by *Fasciola*: A systematic review and meta-analysis

**DOI:** 10.7189/jogh.14.04223

**Published:** 2024-09-19

**Authors:** Zhuo Lan, Xin-Hui Zhang, Jia-Luo Xing, Ai-Hui Zhang, Hong-Rui Wang, Xi-Chen Zhang, Jun-Feng Gao, Chun-Ren Wang

**Affiliations:** 1 Key Laboratory of Bovine Disease Control in Northeast China, Ministry of Agriculture and Rural Affairs, College of Animal Science and Veterinary Medicine, Heilongjiang Bayi Agricultural University, Daqing, Heilongjiang Province, China; 2 Heilongjiang Province Cultivating Collaborative Innovation Center for the Beidahuang Modern Agricultural Industry Technology, Daqing, China; 3 Al-Farabi Kazakh National University, Almaty, Kazakhstan; 4 College of Veterinary Medicine, Jilin University, Changchun, China

## Abstract

**Background:**

Liver disease caused by *Fasciola* is a significant zoonotic and parasitic disease with substantial economic impacts on humans and animals. Many studies have looked at the prevalence of fasciolis worldwide, yet the overall prevalence and risk factors in cattle, ruminants, and humans remains unknown.

**Methods:**

We conducted a systematic review and meta-analysis to estimate the global prevalence and risk factors of fascioliasis in humans and domestic ruminants. With this aim, we searched PubMed, ScienceDirect, Web of Science, and Scopus from inception to 8 December 2022 for studies reporting the prevalence of fascioliasis in humans or domestic ruminants post-2000. We then used random effects models to describe the prevalence of fascioliasis; trim-and-fill analysis and Egger’s test to assess publication bias; and meta-regression and sensitivity analyses to examine the risk factors for prevalence and heterogeneity.

**Results:**

We retrieved 4422 articles, with 371 being included in the analysis, as they concerned fascioliasis in humans and ruminants globally. The pooled prevalence of bovine fasciolosis was 17%, while ovine fasciolosis and human fascioliasis had pooled prevalences of 13% and 5%, respectively. We also conducted subgroup analyses by continents, countries, *Fasciola* species, sampling years, altitude, rainfall, temperature, humidity, age, sex, feeding mode, and residence. Here, altitude and age emerged as risk factors associated with an increased prevalence of fascioliasis. Both the trim-and-fill analysis and Egger’s test confirmed the presence of publication bias, while the sensitivity analysis showed that the omission of any single study did not significantly influence the combined pooled prevalence.

**Conclusions:**

Fascioliasis is a widely prevalent zoonosis among humans and livestock worldwide. Strategies targeting risk factors such as altitude and age are urgently needed for prevention and control of this disease, which will consequently reduce *Fasciola* infection. Additionally, given the inadequacy or absence of data in some countries, greater attention should be paid to *Fasciola* infection, with further epidemiological studies focussing on improving data quality.

*Fasciola hepatica* and *Fasciola gigantica* are two species of liver flukes that parasites the liver and bile ducts of ruminant animals [1,2]. Fasciolid flukes have a two-host life cycle. Adult fasciolids produce eggs within the definitive host; they pass into the bile, enter the intestine, and are shed with the faeces. Once they reach freshwater under suitable environmental conditions, they continue their development. The miracidium stage then develops upon contacting a suitable aquatic or amphibious snail host. Several stages then occur within the intermediate snail host, including miracidium penetration, sporocyst formation, redial generations, and the production of cercariae, which escape from the snail and swim in the water. The cercaria stage develops into the long-resistant metacercaria, facilitating transmission between the snail host and the definitive host [3]. Definitive hosts, such as humans and other mammals, become infected by ingesting metacercariae, resulting in serious health damage.

Humans can become infected with these flukes by consuming aquatic plants on which encysted organisms are present [3]. In fact, human fascioliasis has become a public health concern recently, with the World Health Organization (WHO) classifying these infection as neglected diseases [4]. Fascioliasis is the most widely distributed trematode disease, reported in humans across more than 81 countries worldwide [5]. Its impact on humans has become particularly severe, as human fascioliasis is often overlooked and misdiagnosed, leading to delayed treatment, especially in cases of ectopic parasitism. Some flukes migrate to ectopic sites such as the skin, eyes, brain and neck [6]. For example, a deceased woman from Argentina was found to have *Fasciola hepatica* eggs in her brain [7]. According to statistics, an estimated 2.4 to 17 million people are thought to be infected with liver fluke, and this number is likely increasing [8].

Livestock, mainly sheep and cattle but also goats, equines, and camels, have contributed to the global spread of this disease, especially that the migration of these domesticated animals has been driven by human needs since the post-domestication period [9]. For example, *Fasciola hepatica* is widespread in sheep and cattle rearing areas globally, causing severe morbidity and mortality [10]. Animals with fasciolosis typically suffer from prolonged fever, hepatomegaly, eosinophilia, anorexia, weight loss, anaemia, liver damage, and even death [11,12]. The quality of products from infected animals also declines due to fasciolosis, negatively affecting meat, milk and other products. Furthermore, annual losses due to fasciolosis are estimated at 200 million dollars [13]. Despite this, few studies have reported on the prevalence of fasciolosis in equines and camelids.

It is evident that fascioliasis not only seriously affects the productivity of ruminants, but also threatens human health and the development of the global animal husbandry economy. Therefore, understanding its prevalence and risk factors in humans and livestock worldwide would provide useful information for the prevention and control of this parasitic disease. For this reason, we conducted a systematic review and meta-analysis to assess the prevalence of fascioliasis in humans and domestic animals globally and to analyse the associated risk factors.

## METHODS

### Search strategy and study selection

We followed the PRISMA guidelines in reporting our findings [14]. We searched PubMed, ScienceDirect, Web of Science, and Scopus for studies regarding *Fasciola hepatica* and *Fasciola gigantica* using the search string ‘(*Fasciola hepatica*) OR (*Fasciola gigantica*) OR (fascioliasis) OR (fasciolosis) AND (prevalence)’. We retrieved all published studies on fascioliasis in livestock and humans worldwide from inception to 8 December 2022, without restrictions on geography or language. We imported all retrieved studies into EndNote, version X9 (Clarivate, London, UK) and removed any duplicates. We then screened the studies following predetermined inclusion criteria. Specifically, the studies had to include data on the prevalence of fascioliasis in domestic ruminants or humans after 2000; had to provide the total number of samples and the number of disease-positive cases; had to have a study sample size must be >30; and had to have a cross-sectional and provide raw data for epidemiological analysis. We excluded studies and reviews without valuable data; duplicate studies using the same data; and studies lacking epidemiological data on *Fasciola* infection. We did not contact the original authors for further information, nor did we include unpublished studies (e.g. preprints).

### Data extraction and quality assessment

We extracted data from the studies into a standardised data collection forms in Microsoft Excel, version 16.32 (Microsoft, Redmond, WA, USA). These data included the first author; publication year; sampling year; the geographical region of the study (including continent and country); altitude; rainfall; age; sex; sampling method; feeding model; residence; the total number of domestic ruminants and humans examined; and the number testing positive for fascioliasis in domestic ruminants and humans. We also recorded the species of *Fasciola* in the study. If the samples were analysed using multiple methods, we prioritised the data obtained from microscopy for analysis.

We assessed the quality of the included studies using the Grading of Recommendations, Assessment, Development, and Evaluations (GRADE) criteria [15]. Briefly, we gave a study one point if any of the following factors were described: sampling year; sample size >60; sampling method; and four or more potential risk factors. Thus, the possible total score ranged from 0 to 5, with three levels of classification. Studies with a total score of 4 or 5 were classified as high-quality, those with a score of 2 or 3 as medium quality, and those with a score of 0 or 1 as low quality.

### Statistical analysis

We performed all quantitative analyses using the ‘Meta’ package, R, version 4.11.0. (R Core Team, Vienna, Austria). We used a random effects model to provide a pooled estimate for the overall assessment of the meta-analysis and subgroup analyses, and we further presented the results using forest plots. We otherwise used funnel plots, trim-and-fill analysis, and Egger’s test to evaluate publication bias, and the I^2^ and Cochran’s Q statistics to quantify heterogeneity which we further investigated using either individual models or multivariable models. We also performed sensitivity analyses to assess the stability of the results. In the multivariable model, the category with the highest prevalence in the subgroup analysis was taken as the reference group. Lastly, we conducted meta-regression analyses to examine risk factors affecting prevalence and heterogeneity. *P*-values <0.05 indicated statistical significance.

## RESULTS

### Search results and eligible studies

**Figure 1:**
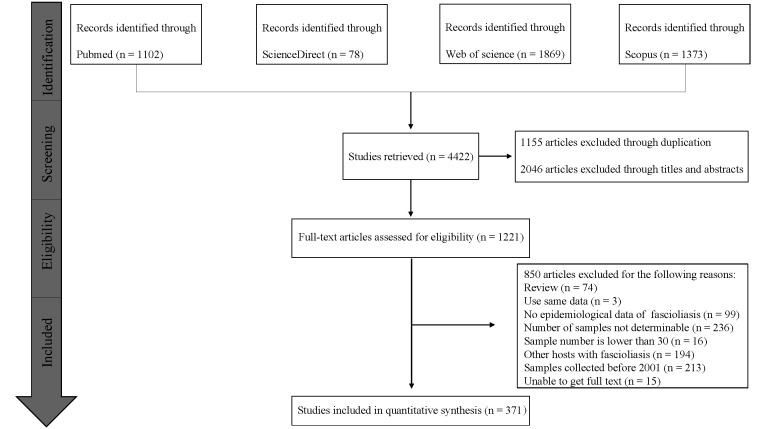
Flow diagram of literature search and selection of fasciolosis in human and domestic ruminants in the world.

We retrieved 4422 records from the four databases. Based on the inclusion and exclusion criteria, we included 371 full-text studies (comprising 162 high-quality, 203 medium-quality, and 6 low-quality studies) in the meta-analysis (**Figure 1**; Table S1 in the **Online Supplementary Document**).

### Pooling and heterogeneity analyses

**Figure 2:**
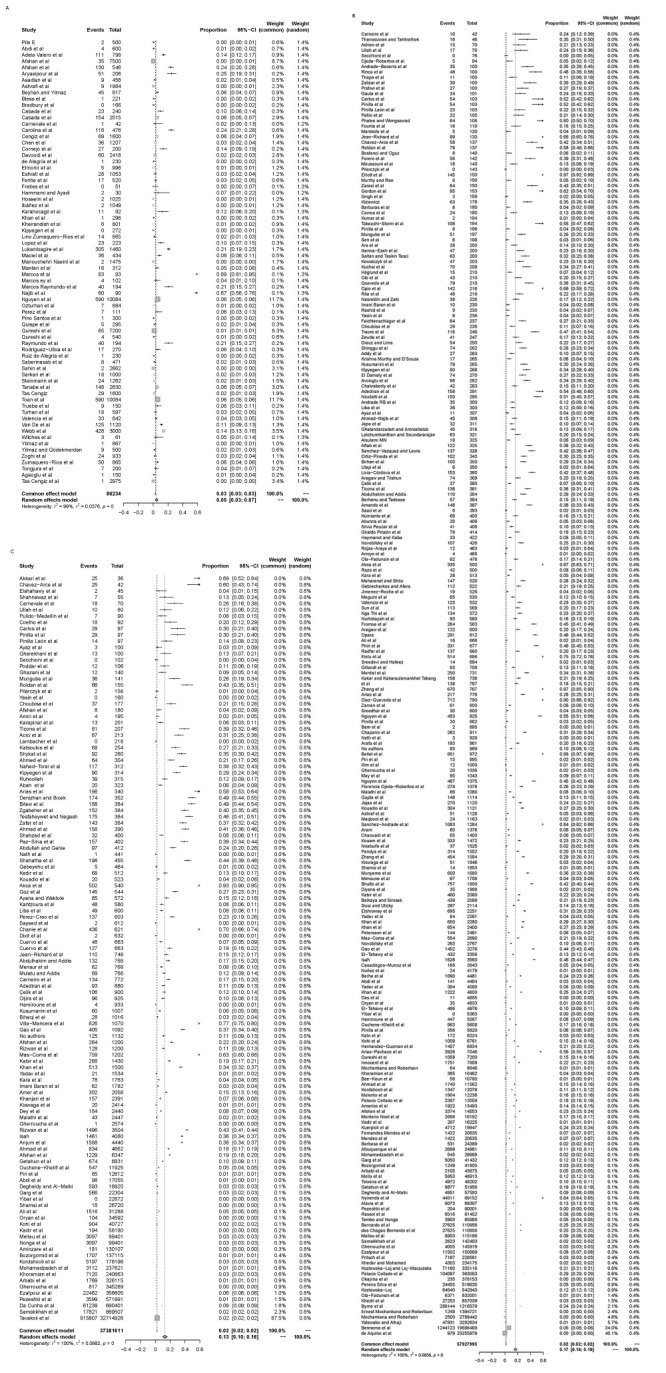
Forest plot of prevalence of fasciolosis in human and domestic ruminants among studies conducted in the world: **Panel A.** In humans. **Panel B.** In cattle. **Panel C.** In small ruminants.

We used a forest plot to measure and display the heterogeneity index and a random-effects model to estimate the prevalence for each subgroup. The overall infection rate in humans was 5.00% (n/N = 3846/86 234). Globally, the pooled prevalence of bovine fasciolosis was 17.00% (n/N = 2 143 908/57 927 995), and that of ovine fasciolosis was 13% (n/N = 968 397/37 381 611) (**Figure 2**, panels A–C).

**Figure 3:**
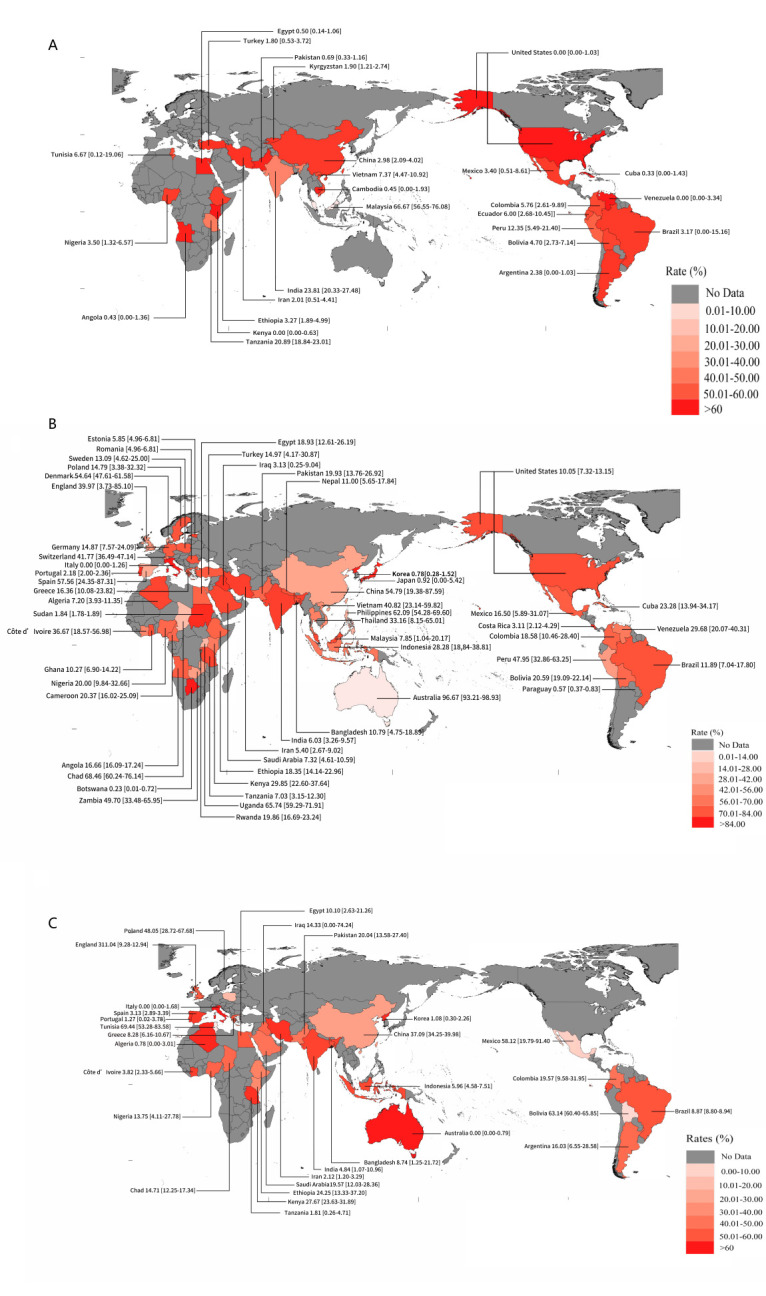
Map of fasciolosis in the world: **Panel A.** In humans. **Panel B.** In cattle. **Panel C.** In small ruminants.

The prevalence of fascioliasis varied significantly across different continents and hosts, such as in cattle and sheep (*P* = 0.0003) and in humans (*P* = 0.0082). For example, we found the highest prevalence in humans to be in South America (9.09%). Between continents, the prevalence in cattle ranged from 12.02% to 96.67%, while the prevalence in sheep ranged from 9.08% to 58.12% (**Table 1**). At the country level, we observed the highest prevalence among humans in Malaysia at 66.67%, among cattle in Australia at 96.67%, and among ovine fasciolosis in Tunisia at 69.44% (**Figure 3**, panels A–C).

**Table 1:** Pooled prevalence of fasciolosis (fascioliasis) infection in domestic ruminants and humans worldwide

					Heterogeneity			Univariate meta-regression	
	**Number of studies**	**Number examined**	**Number positive**	**% (95% CI）**	**χ^2^**	***P*-value**	**I*^2^* (%)**	***P*-value**	**Coefficient (95% CI)**
**Continent**									
Cattle									
*Asia*	96	2 220 922	79 150	12.02 (9.08, 15.31)	94 184.34	<0.001	99.9	<0.0001	−1.0330 (−1.5243, −0.5418)
*Africa*	52	8 960 344	149 715	18.03 (13.06, 23.60)	372 150.57	<0.001	100.0	0.0002	−0.9487 (−1.4421, −0.4553)
*South America*	42	44 091 663	1 354 073	20.59 (14.82, 27.02)	2 828 800.83	<0.001	100.0	0.0003	−0.9163 (−1.4107, −0.4218)
*North America*	8	385 515	108 246	12.90 (6.47, 21.12)	1692.15	<0.001	99.6	0.0001	−1.0213 (−1.5392, −0.5034)
*Europe*	9	22 67 656	452 035	20.63 (14.29, 27.80)	56 588.03	<0.001	99.9	0.0003	−0.9156 (−1.4105, −0.4207)
*Oceania*	1	150	145	96.67 (93.21, 98.93)	0.00	-	-	-	-
Sheep									
*Asia*	65	36 097 190	892177	9.08 (5.92, 12.84)	79 103.99	<0.001	99.9	0.0019	−0.5592 (−0.9118, −0.2067)
*Africa*	28	485 201	8851	14.70 (8.61, 22.06)	19 624.00	<0.001	99.9	0.0101	−0.4720 (−0.8315, 0.1125)
*South America*	11	693 629	62 376	25.61 (14.94, 37.97)	2133.24	<0.001	99.5	0.0003	−0.3337 (−0.7119, 0.0445)
*North America*	2	1382	943	58.12 (19.79, 91.40)	165.18	<0.01	99.4	-	-
*Europe*	10	3782	751	15.45 (4.86, 30.42)	754.53	<0.01	98.8	0.0178	−0.4605 (−0.8413, −0.0798)
*Human*									
*Asia*	35	65 318	2115	3.09 (1.39, 5.41)	2458.58	<0.001	98.6	0.0082	−0.1305 (−0.2273, − 0.0337)
*Africa*	8	3938	338	2.66 (0.19, 7.26)	535.66	<0.01	98.7	0.0766	−0.1365 (−0.2877, 0.0146)
*South America*	25	14 782	1328	9.09 (4.57, 14.90)	1131.59	<0.01	97.9	-	-
*North America*	4	2196	65	1.40 (0.02, 4.36)	47.14	<0.01	93.6	0.0622	−0.1889 (−0.3874, 0.0096)
***Fasciola* species**									
Cattle									
*Fasciola hepatica*	106	46 873 889	1 908 529	18.14 (14.40, 22.22)	4 091 463.16	<0.001	100.0	-	-
*Fasciola gigantica*	38	4 241 009	11 039	14.00 (8.11, 21.17)	28 518.45	<0.001	99.9	0.2751	−0.0567 (−0.1587, 0.0452)
Small ruminants									
*Fasciola Hepatica*	48	1 100 578	66 603	17.58 (12.13, 23.77)	70 114.00	<0.001	99.9	-	-
*Fasciola Gigantica*	12	22 872	2527	10.21 (4.01, 18.74)	6774.10	<0.001	99.8	0.2047	−0.1061 (−0.2702, 0.0579)
Human									
*Fasciola Hepatica*	42	31 195	1140	3.99 (1.92, 6.72)	1840.00	<0.001	97.8	-	-
*Fasciola Gigantica*	1	1207	36	2.98 (2.09, 4.02)	0.00	-	97.7	0.8701	−0.0322 (−0.4179, 0.3535)
**Altitude**									
Cattle									
*Low altitude (≤1200 m)*	20	205 561	20 166	13.07 (6.42, 21.63)	8414.19	<0.001	99.8	0.0145	−0.1519 (−0.2737, −0.0301)
*High altitude (>1200 m)*	38	117 077	15 771	24.86 (19.46, 30.68)	13 077.49	<0.001	99.7	-	-
Sheep									
*Low altitude*	8	312 267	64 297	11.48 (3.70, 22.74)	6004.43	<0.001	99.9	0.0694	−0.4252 (−0.2536, 0.0163)
*High altitude*	23	21 768	7673	27.30 (17.38, 38.50)	14 045.40	<0.001	99.8	-	-
Human									
*Low altitude*	5	9415	90	2.34 (0.30, 5.84)	95.02	<0.01	95.8	0.0724	−0.1167 (−0.2440, 0.0106)
*High altitude*	13	4017	482	7.55 (4.21, 11.72)	302.24	<0.01	96.0	-	-
**Rainfall**									
Cattle									
*<800*	21	491 524	20 058	19.21 (12.76, 26.63)	24 256.73	<0.001	99.9	0.8147	−0.0147 (−0.1381, 0.1086)
*≥800*	35	3 512 789	69 003	20.42 (14.41, 27.17)	36 121.23	<0.001	99.9	-	-
Sheep									
*<800*	15	1 094 090	12 705	18.86 (8.70, 31.76)	11 654.64	<0.001	99.9	-	-
*≥800*	18	245 654	6376	17.07 (9.08, 26.91)	4515.22	<0.001	99.6	0.8215	−0.0216 (−0.2093, 0.1661)
Human									
*<800*	6	2522	178	4.61 (0.26, 13.14)	336.28	<0.01	98.5	-	-
*≥800*	2	792	17	1.04 (0.00, 6.47)	16.97	<0.01	94.1	0.4299	−0.1145 (−0.3989, 0.1698)
**Age in years**									
Cattle									
*>2*	50	2 107 055	21 392	27.63 (21.30, 34.44)	23 803.57	<0.001	99.8	-	-
*≤2*	35	11 873	2215	22.66 (15.53, 30.70)	1920.33	<0.001	98.2	0.3357	−0.0573 (−0.1740, 0.0594)
Sheep									
*>2*	13	3302	1116	37.03 (25.22, 49.67)	434.02	<0.01	97.2	-	-
*≤2*	21	5278	1101	18.26 (11.21, 26.52)	1085.40	<0.01	98.2	0.0098	−0.2102 (−0.3696, −0.0507)
Human									
*0–17*	23	19 654	1181	4.14 (1.94, 6.99)	1428.59	<0.01	98.5	-	-
*18–65*	11	5861	188	3.29 (0.74, 7.44)	300.58	<0.01	96.7	0.5032	−0.0357 (−0.1401, 0.0688)
*>65*	3	137	4	2.33 (0.00, 10.39)	6.28	0.04	68.1	0.7312	−0.0336 (−0.2256, 0.1583)
**Sex**									
Cattle									
*Male*	53	101 903	11 024	18.88 (15.07, 23.02)	4791.63	<0.001	98.9	0.3011	−0.0399 (−0.1157, 0.0358)
*Female*	51	24 897	5211	22.16 (17.71, 26.97)	3654.45	<0.001	98.6	-	-
Sheep									
*Male*	27	19 259	4316	21.24 (14.26, 29.16)	3509.67	<0.001	99.3	0.4304	−0.0461 (−0.1608, 0.0685)
*Female*	27	40 586	4602	25.40 (19.44, 31.86)	4470.90	<0.001	99.4	-	-
Human									
*Men*	30	21 811	727	4.00 (1.77, 7.00)	899.98	<0.01	96.8	-	-
*Women*	30	22 118	835	3.96 (1.94, 6.60)	955.25	<0.01	97.0	0.9612	0.0022 (−0.0853, 0.0897)
**Feeding mode**									
Cattle									
*Feed*	5	1304	347	13.57 (4.90, 25.65)	149.84	<0.01	97.3	0.1757	−0.1756 (−0.4297, 0.0786)
*Graze*	11	18 516	3865	27.41 (14.94, 42.00)	2268.73	<0.001	99.6		
Sheep									
*Feed*	1	47	6	12.77 (4.49, 24.05)	0.00	-	-	0.3493	−0.1925 (−0.5958, 0.2107)
*Graze*	9	5071	1502	28.96 (18.66, 40.48)	404.62	<0.01	98.0		
**Residence**									
Human									
*Urban*	6	2798	13	0.45 (0.07, 1.07)	13.36	0.02	62.6	0.4644	−0.0194 (−0.0714, 0.0326)
*Rural*	12	12570	124	0.82 (0.25, 1.64)	50.52	<0.01	78.2	-	-

CI – confidence interval, χ^2^ – chi-squared

The prevalence of *Fasciola hepatica* was higher than that of *Fasciola gigantica* across all hosts, with the former being more common overall (although this difference was not statistically significant) (**Table 1**). In the subgroup analysis based on altitude, the prevalence of bovine fasciolosis was 24.86% at high altitudes and 13.07% at low altitudes, with a significant difference (*P* = 0.0145). While we found no significant difference in the other two hosts, they also showed higher prevalence at high altitudes. Subgroup analysis based on rainfall found that bovine prevalence was higher when annual rainfall was ≥800 mm (n/N = 69 003/3 512 789, 20.42%), whereas it was higher when rainfall was <800 mm in small ruminants (n/N = 12 705/1 094 090, 18.86%) and humans (n/N = 178/2522, 4.61%). However, the differences between the groups were not significant.

Regarding age, there was no significant difference in prevalence between different age groups in cattle and humans. Cattle older than 2 years had a higher infection rate (n/N = 21 392/2 107 055, 27.63%) than cattle younger than 2 years, while the highest infection rate in humans was among those younger than 17 years (n/N = 1181/19 654, 4.14%). The infection rate in sheep and goats older than 2 years was significantly higher than in those younger than 2 years, with a large difference observed in the prevalence of ovine fasciolosis across different age groups (*P* = 0.0098). The subgroup analysis by sex showed that the prevalence was higher in females than males in livestock, while we observed the opposite trend in humans. We also saw that grazing resulted in a higher infection rate in both cattle (n/N = 3865/18 516, 27.41%), and sheep and goats (n/N = 1502/5071, 28.96%) than feeding. Humans living in rural areas (n/N = 124/12 570, 0.82%) showed a higher infection rate than those in urban areas (n/N = 13/2798, 0.45%) (**Table 1**).

### Publication bias and sensitivity analysis

The funnel plot indicated possible publication bias in the included studies, as did the results of the trim-and-fill analysis. For the Egger’s test, the intercept values were not significant at 0.1593, 0.1564, and 0.1277 in the analysis for humans, sheep, and cattle, respectively. The sensitivity test showed that the combined pooled prevalence was not significantly affected by the omission of any study (Figures S1–4 in the **Online Supplementary Document**), indicating that the meta-analysis results were reliable.

## DISCUSSION

To the best of our knowledge, this is the first systematic review and meta-analysis to estimate the pooled prevalence of fascioliasis in both humans and domestic ruminants globally. Although our funnel plot analyses indicated that publication bias existed in the included studies, it is worth noting that such bias may arise in cases where a small number of studies report significant results, as the likelihood of obtaining significant findings increases with sample size. This suggests that publication bias predominantly came from the studies with small sample sizes; in fact, we can see that studies with small samples are located at the bottom of our study’s funnel plot, while large sample studies are at the top. These small sample studies represent a relatively small proportion of the total, so their impact on our findings is likely minimal. Additionally, Egger’s test results indicated no publication bias. Overall, the results of this study are reliable, and the influence of small sample studies on the findings is minimal. The results indicated that all seven continents, except Antarctica, reported cases of fascioliasis. Specifically, six (55 countries) reported bovine fasciolosis, five (30 countries) reported ovine fasciolosis, and four (26 countries) reported human fascioliasis. There were significant differences in the infection rates of fascioliasis among humans and domestic ruminants across different continents, with the highest prevalence for humans being observed in South America. Prior studies highlighted that South American countries such as Bolivia, Ecuador, and Peru bear a substantial burden of human fascioliasis, supporting our findings [16,17]. In mammal hosts, the data showed that Oceania had the highest prevalence of cattle fasciolosis, while North America had the highest prevalence of ovine fasciolosis; however, these two findings were based on one and two studies, respectively, meaning they may not accurately reflect the prevalence in these continents. Additionally, the prevalence of ovine and bovine fasciolosis was notably higher in South America. This may be due to the significant impact of the Andes on the area’s climate, which favours the adaptation of *Fasciola* and its intermediate snail hosts to high altitude environments. Conversely, the prevalence of fascioliasis was lower in North America, with no reported infection rate in humans, lower prevalence in cattle, and no data available for sheep. This discrepancy may be related to local dietary habits and living standards.

Overall, 106 studies reported the prevalence of *Fasciola hepatica* in cattle, while 38 focussed on *Fasciola gigantica*. The disparity in the number of studies was similarly observed in analyses of humans and small ruminants, indicating that *Fasciola hepatica* is more widely distributed globally. This finding is consistent with the results reported by Carmona and Tort [16]. Yet it is worth noting that the damage caused by both species is severe, making it crucial to strengthen the prevention and control measures for fascioliasis.

Grazing livestock also had a higher infection rate than those raised in captivity on feed. The distribution of *Fasciola hepatica* is known to be influenced by management factors [18]. To mitigate the impact of fasciolosis, improved management practices, including better sanitation and biosecurity measures, should be implemented to enhance the resistance of bovines to the disease, while increasing awareness and implementing effective prevention and control measures for fascioliasis in humans also remains essential in this context.

Howell et al. [19] demonstrated that higher rainfall, grazing in boggy areas, and other factors were associated with an increased risk of *Fasciola hepatica* exposure, which aligns with our results. However, we observed contrasting findings in the ovine and human analyses. One study found that prevalence was higher during warm, wet periods compared to cold, dry ones, with the lowest peak occurring during periods of lowest temperatures, despite a high rainfall curve [20]. This suggests that not only does rainfall affect infection rates, but temperature also plays a significant role. Regions with lower rainfall may experience higher temperatures, which could further influence the prevalence of infection in ovine and human analyses. Unfortunately, we were unable to conduct subanalyses by temperature here due to the scarcity of studies addressing this condition.

At higher altitudes, the prevalence of fascioliasis was significantly higher across all hosts, with a notable difference observed in bovine fasciolosis. Mas-Coma's study indicated that both the liver fluke and the lymnaeid snail host have adapted to the extreme environmental conditions of high altitudes, leading to high infection rates [21]. As the intermediate host, the snail provides living conditions that facilitate the survival and development of liver flukes, indirectly contributing to higher infection rates of *Fasciola*. 

A prior study reported that the longevity of infected snails in high-altitude areas is longer compared to the duration of infection in *Fasciola hepatica*-infected *Galba truncatula* in lowland regions [22]. This may explain why infection rates are higher at higher altitudes. Additionally, although high mountainous regions remain relatively remote and sparsely populated, the growing demand for land has led to increased human activity at high altitudes (such as animal husbandry and grazing), which could further contribute to the spread of the disease. This makes high altitude a significant factor in the prevalence of fascioliasis.

Our results further showed that older cattle have a higher infection rate of fasciolosis, which is consistent with the finding of Zewde et al. in Ethiopia [23]. This may be attributed to the long-term grazing of older cattle compared to the indoor feeding and management of younger cattle. Additionally, the immunity of older cattle is often weakened. Management strategies such as improved sanitation, biosecurity measures, and centralised management in intensive farming may improve the resistance of cattle to fasciolosis. We had similar findings for small ruminants, with a highly significant difference (*P* = 0.0098). However, we had different findings for humans, whereby individuals aged 0–17 had the highest prevalence. This is likely because children and teenagers are more likely to engage in activities near the water; put various sylvatic herbs into their mouths for eating, sucking, chewing, or stripping with their teeth; and may lack awareness about fascioliasis, which significantly increases the incidence of the disease. This finding is consistent with the study by Mas-Coma et al. [24].

In the subgroup analysis by sex among humans, men had a slightly higher prevalence than women, so both men and children under 17 years of age should be attentive to the prevention of fascioliasis. However, we had different findings for the animals in our analysis, whereby females appeared to have a slightly higher prevalence of fasciolosis than males in livestock – although this difference was not significant difference. A similar result was reported in the UK, where a higher infection rate of *Fasciola hepatica* was found in female cattle [25]. Several factors may contribute to this higher infection rate in females, such as increased stress during the parturition period, which can decrease individual immunity and increase susceptibility to infection.

This meta-analysis has some limitations. First, although we used four databases to retrieve relevant studies, we may have overlooked some studies. Second, countries such as Kyrgyzstan, Denmark, Australia, and 17 other countries in this review were represented by only one study, which prohibited any robust analyses. Third, although data were reported from 81 countries worldwide, we could only conduct analyses for 58. This discrepancy may be due to the removal of some studies which did not fit our inclusion criteria. Fourth, as there is no gold standard method for diagnosing *Fasciola* infection, the included studies used various diagnostic methods. Since eggs can only be detected in the faeces after the worms have matured into adults, this may reduce the number of positive samples. Identifying a gold standard method for diagnosing fascioliasis is essential for future research. Yet despite this limitation, Mazeri et al. [26] evaluated the performance of different diagnostic tests for *Fasciola* infection using a Bayesian approach and found that all methods were valuable. Although prior studies reported a low number of observed cases, their findings likely reflect the infection status correctly, as they were detected using multiple methods. This means that a low number of observed cases might indicate that the situation is not severe, rather than connote a deficiency in the diagnostic system. Finally, we were unable to conduct direct comparisons between different countries for several reasons, one such being that we lacked sufficient understanding of the local conditions. Nevertheless, the infection rates in different countries were clearly described in the studies.

## CONCLUSIONS

In this systematic review and meta-analysis, we determined that liver disease caused by *Fasciola* is a prevalent condition worldwide, with altitude and age being related to increased prevalence as risk factors. However, other possibly relevant factors should not be ignored. We suggest that efforts focus on understanding of the spread and impact of *Fasciola *at high altitudes, as well as improving animal husbandry management, reducing grazing, and shifting attention to the health of older populations. These data could be highly valuable for policymakers in designing and implementing prevention and control programmes, particularly in high infection areas.

## Additional material


Online Supplementary Document
