# Supplementary material for: Global prevalence of liver disease in human and domestic animals caused by Fasciola: A systematic review and meta-analysis
Source: J Glob Health. 2024 Sep 19;14:04223. doi: 10.7189/jogh.14.04223 (PMC11412093; doi:10.7189/jogh.14.04223)
Supplement: Online Supplementary Document [file jogh-14-04223-s001.pdf]

Table S1 Studies included in the analysis of fasciolosis in domestic ruminants and human worldwide.

| Study | Host | Sampling years | Country | *Fasciola* species | Altitude | Rainfall | Stage | Sex | Feeding mode | Residence | Number | Case | Score | Quality |
|---|---|---|---|---|---|---|---|---|---|---|---|---|---|---|
| Abah et al (2019) [1] | Sheep | 2015-2016 | Nigeria | / | N | N | N | Y | N | / | 323 | 20 | 4 | High |
| Abdi et al. (2013) [2] | Cattle | 2012 | Iran | *F.hepatica* | N | N | N | N | N | | 4484 | 141 | 3 | Middle |
| | Sheep | 2012 | Iran | *F.hepatica* | N | N | N | N | N | | 17055 | 98 | 3 | Middle |
| | Human | 2012 | Iran | *F.hepatica* | N | N | N | N | / | Y | 600 | 4 | 3 | Middle |
| Abdulhakim and Addis (2012) [3] | Sheep | 2010-2011 | Ethiopia | / | Y | N | Y | N | N | / | 384 | 80 | 4 | High |
| | Cattle | 2010-2011 | Ethiopia | / | Y | N | Y | N | N | / | 384 | 110 | 4 | High |
| Abdullah and Ganie (2020) [4] | Sheep | 2017-2018 | India | / | N | N | N | Y | N | / | 412 | 97 | 3 | Middle |
| Abunna et al (2010) [5] | Cattle | 2008 | Ethiopia | *Fasciola* | Y | Y | N | N | N | / | 406 | 20 | 4 | High |
| Acici et al (2017) [6] | Sheep | 2005-2007 | Turkey | *F.hepatica* | N | N | N | N | Y | / | 213 | 67 | 4 | High |
| Addy et al (2020) [7] | Cattle | 2018 | Ghana | / | N | N | Y | Y | N | / | 70 | 15 | 3 | Middle |
| Adediran et al (2014) [8] | Sheep | 2011 | Nigeria | / | N | N | N | N | N | / | 880 | 93 | 4 | High |
| | Cattle | 2011 | Nigeria | / | N | N | N | N | N | / | 291 | 158 | 4 | High |
| Adela et al (2012) [9] | Human | 2008 | Peru | *F.hepatica* | N | N | Y | N | / | N | 436 | 94 | 3 | Middle |
| Adrien et al (2013) [10] | Cattle | 2011 | Brazil | *F.hepatica* | N | N | N | N | N | / | 70 | 15 | 3 | Middle |
| Afshan et al (2020) [11] | Human | 2014-2016 | India | / | N | N | N | N | N | / | 546 | 130 | 4 | High |
| Afshan et al (2014) [12] | Human | 2003-2005 | Pakistan | / | Y | N | N | N | / | N | 7500 | 35 | 3 | Middle |
| | Cattle | 2001-2011 | Pakistan | *Fasciola* | Y | N | N | N | N | / | 14553 | 3374 | 3 | Middle |
| Afshan et al (2013) [13] | Sheep | 2008-2009 | Pakistan | *F.hepatica* | N | N | N | N | Y | / | 612 | 240 | 4 | High |
| Aftab et al (2020) [14] | Cattle | / | India | *F.giganticaa* | N | N | N | N | N | / | 325 | 122 | 2 | Middle |
| Agaoglu et al (2004) [15] | Human | / | Turkey | *F.hepatica* | N | N | N | N | / | N | 150 | 1 | 2 | Middle |
| Ahmad et al (2021) [16] | Cattle | 2017-2018 | Malaysia | / | N | N | Y | Y | Y | / | 308 | 45 | 5 | High |
| Ahmad et al (2020) [17] | Cattle | 2016-2017 | Nigeria | *F.giganticaa* | N | Y | N | N | Y | / | 11562 | 1740 | 4 | High |

| | | | | | | | | | | | | | | |
|---|---|---|---|---|---|---|---|---|---|---|---|---|---|---|
| Ahmad et al (2017) [18] | Sheep | 2012 | Pakistan | / | N | Y | N | N | N | / | 390 | 158 | 4 | High |
| Ahmad et al (2017) [19] | Sheep | 2012 | Pakistan | / | Y | Y | Y | Y | N | / | 2242 | 594 | 5 | High |
| Ahmed et al (2005) [20] | Sheep | 2001-2002 | Pakistan | *Fasciola* | N | N | N | Y | N | / | 261 | 52 | 4 | High |
| Akca et al (2014) [21] | Cattle | / | Turkey | *F.hepatica* | Y | N | N | N | N | / | 500 | 335 | 4 | High |
| | Sheep | / | Turkey | *F.hepatica* | Y | N | N | N | N | / | 540 | 502 | 4 | High |
| Akkari et al (2011) [22] | Sheep | 2004-2005 | Tunisia | *F.hepatica* | Y | N | N | N | N | / | 36 | 25 | 4 | High |
| Alawa et al (2011) [23] | Cattle | 200-2005 | Nigeria | / | N | N | N | N | N | / | 69307 | 9073 | 3 | Middle |
| Albuquerque et al. (2022) [24] | Cattle | 2015-2017 | Brazil | / | N | N | N | N | N | / | 24961 | 2699 | 3 | Middle |
| Ali et al (2011) [25] | Cattle | 2007-2009 | Iran | / | N | N | N | Y | N | / | 666 | 16 | 3 | Middle |
| | Sheep | 2007-2009 | Iran | / | N | N | N | Y | N | / | 31288 | 1516 | 3 | Middle |
| Alsulami et al (2022) [26] | Cow | 2020 | Saudi Arabia | *F.hepatica* | N | N | Y | Y | N | / | 325 | 18 | 4 | High |
| Amer et al (2016) [27] | Sheep | 2012-2014 | Egypt | / | N | N | N | N | N | / | 2058 | 302 | 3 | Middle |
| Americo et al. (2022) [28] | Cattle | 2018-2021 | Brazil | *F.hepatica* | N | N | N | N | N | / | 13493 | 1922 | 4 | High |
| Aminzare et al (2018) [29] | Sheep | 2016-2017 | Iran | / | N | N | N | N | N | / | 130107 | 181 | 3 | Middle |
| Amiri et al (2021) [30] | Sheep | / | Iran | *F.hepatica* | | N | N | N | N | / | 195 | 4 | 3 | Middle |
| Andrade et al (2020) [31] | Cattle | / | Colombia | / | N | Y | Y | Y | Y | / | 300 | 35 | 4 | High |
| Andrade et al (2020) [32] | Cattle | 2020 | Colombia | / | Y | Y | Y | N | N | / | 100 | 35 | 4 | High |
| Anjum et al. (2014) [33] | Sheep | 2011-2012 | Pakistan | / | Y | N | N | Y | N | / | 2172 | 689 | 4 | High |
| Ara et al (2021) [34] | Cattle | 2018-2019 | Bangladesh | *F.giganticaa* | N | N | Y | Y | N | / | 200 | 28 | 5 | High |
| Arafa et al (2018) [35] | Cattle | 2014 | Egypt | *F.hepatica* | N | Y | Y | N | N | / | 961 | 193 | 4 | High |
| Aragaw and Tilahun (2019) [36] | Cattle | 2011-2012 | Ethiopia | / | Y | Y | Y | Y | N | / | 369 | 74 | 5 | High |
| Aragaw et al (2012) [37] | Cattle | 2010-2011 | Ethiopia | *Fasciola* | Y | N | N | N | N | / | 600 | 122 | 5 | High |
| Aram (2020) [38] | Cattle | 2018 | Iraq | / | N | N | N | N | N | / | 1376 | 80 | 4 | High |
| Arbabi et al (2018) [39] | Sheep | 2013-2016 | Iran | / | Y | Y | N | N | N | / | 118463 | 895 | 4 | High |

| | | | | | | | | | | | | | | |
|---|---|---|---|---|---|---|---|---|---|---|---|---|---|---|
| | Sheep | 2013-2016 | Iran | / | Y | Y | N | N | N | / | 43675 | 2105 | 4 | High |
| Arias et al (2011) [40] | Cattle | 2007-2009 | Spain | *F.hepatica* | Y | N | Y | Y | Y | / | 217 | 28 | 4 | High |
| Arias et al (2007) [41] | Sheep | / | Spain | *F.hepatica* | N | N | N | N | Y | / | 340 | 198 | 2 | Middle |
| Arias et al (2020) [42] | Cattle | 2013-2014 | Peru | *F.hepatica* | Y | N | N | N | N | / | 7046 | 3926 | 4 | High |
| Arroyo et al (2022) [43] | Cattle | 2018-2020 | Colombia | *F.hepatica* | Y | Y | N | N | N | / | 466 | 4 | 4 | High |
| Aryaeipour et al (2015) [44] | Human | 2008-2014 | Iran | / | N | N | Y | Y | / | N | 206 | 51 | 3 | Middle |
| Asadian et al (2013) [45] | Human | 2012 | Iran | / | N | N | Y | N | / | N | 458 | 9 | 3 | Middle |
| Ashraf et al (2017) [46] | Cattle | / | India | / | Y | Y | Y | N | N | / | 1126 | 51 | 3 | Middle |
| Ashrafi et al (2015) [47] | Human | 2008-2011 | Iran | *F.hepatica* | N | N | N | Y | / | Y | 1984 | 9 | 5 | High |
| Avcioglu et al (2014) [48] | Cattle | 2011 | Turkey | / | Y | Y | Y | Y | Y | / | 282 | 96 | 4 | High |
| Ayana and Waktole (2013) [49] | Sheep | 2010-2011 | Ethiopia | / | Y | Y | N | Y | N | / | 572 | 85 | 4 | High |
| Ayaz et al (2013) [50] | Sheep | 2012 | Pakistan | *F.hepatica* | N | N | Y | Y | N | / | 100 | 3 | 5 | High |
| Ayaz et al (2014) [51] | Cattle | / | Pakistan | *F.hepatica* | N | N | N | N | N | / | 307 | 11 | 2 | Middle |
| Bae et al (2015) [52] | Cattle | 2014 | Korea | / | N | N | Y | N | N | / | 10780 | 58 | 4 | High |
| Balkaya and Simsek (2010) [53] | Cattle | 2008-2009 | Turkey | *F.hepatica* | N | N | N | N | N | / | 2088 | 439 | 3 | Middle |
| Bam et al (2012) [54] | Cattle | 2010-2011 | India | *F.giganticaa* | N | N | N | N | N | / | 895 | 2 | 3 | Middle |
| Barbosa et al (2019) [55] | Cattle | 2015-2016 | Portugal | / | N | N | N | N | N | / | 24389 | 531 | 3 | Middle |
| Barburas et al (2021) [56] | Cattle | / | Romania | *F.hepatica* | N | N | Y | Y | N | / | 180 | 8 | 4 | High |
| Bellet et al (2016) [57] | Cattle | 2014-2015 | England | *F.hepatica* | N | N | N | N | N | / | 972 | 951 | 3 | Middle |
| Bennema et al (2014) [58] | Cattle | 2002-2011 | Brazil | *F.hepatica* | N | N | N | N | N | / | 19696469 | 1244123 | 3 | Middle |
| Berhanu and Tadesse (2018) [59] | Cattle | 2015 | Ethiopia | / | Y | Y | N | Y | N | / | 384 | 57 | 5 | High |

| | | | | | | | | | | | | | | |
|---|---|---|---|---|---|---|---|---|---|---|---|---|---|---|
| Berhe et al (2009) [60] | Cattle | 2006-2007 | Ethiopia | *Fasciola* | N | N | N | N | N | / | 4481 | 1090 | 3 | Middle |
| Bernardo et al (2011) [61] | Cattle | 2006-2009 | Brazil | *F.hepatica* | N | N | N | N | N | / | 110956 | 27625 | 3 | Middle |
| Beyhan and Yilmaz (2020) [62] | Human | 2011-2018 | Turkey | *F.hepatica* | N | N | N | Y | / | N | 817 | 45 | 3 | Middle |
| Bhutto et al (2012) [63] | Cattle | 2004-2005 | Pakistan | *F.giganticaa* | N | N | N | N | N | / | 1800 | 757 | 4 | High |
| Bihaqi et al (2017) [64] | Sheep | 2011-2012 | India | / | Y | Y | Y | Y | N | / | 1016 | 28 | 5 | High |
| Birhan et al (2019) [65] | Cattle | 2018 | Ethiopia | / | N | Y | N | Y | N | / | 350 | 100 | 4 | High |
| Bitew et al (2010) [66] | Sheep | 2009-2010 | Ethiopia | / | Y | Y | Y | Y | Y | / | 384 | 188 | 4 | High |
| Bless et al (2015) [67] | Human | 2011 | Cambodia | / | N | N | N | N | / | Y | 221 | 1 | 3 | Middle |
| Bostanci and Oguz (2017) [68] | Cattle | / | Turkey | *F.hepatica* | Y | N | Y | Y | Y | / | 140 | 8 | 3 | Middle |
| Bozorgomid et al (2018) [69] | Cattle | 2014-2016 | Iran | / | N | Y | N | N | N | / | 41805 | 1249 | 4 | High |
| | Sheep | 2014-2016 | Iran | / | N | Y | N | N | N | / | 137155 | 1707 | 4 | High |
| Bradbury et al (2020) [70] | Human | 2016-2017 | America | *F.hepatica* | N | N | Y | N | / | N | 166 | 0 | 3 | Middle |
| Byrne et al (2016) [71] | Cattle | 2011-2013 | England | *F.hepatica* | N | N | N | N | N | / | 1216579 | 288144 | 3 | Middle |
| Cabada et al (2015) [72] | Human | / | Peru | *F.hepatica* | N | Y | Y | N | / | N | 240 | 23 | 3 | Middle |
| Cabada et al (2018) [73] | Human | 2013-2017 | Peru | *F.hepatica* | Y | N | Y | N | / | N | 2515 | 154 | 4 | High |
| Carlos et al (2020) [74] | Cattle | 2017-2018 | Colombia | *F.hepatica* | Y | Y | Y | Y | N | / | 103 | 54 | 5 | High |
| | Sheep | 2017-2018 | Colombia | *F.hepatica* | Y | Y | Y | Y | N | / | 97 | 29 | 5 | High |
| Carneiro et al (2013) [75] | Sheep | / | / | *F.hepatica* | N | N | N | N | N | / | 424 | 58 | 2 | Low |
| | Cattle | / | / | *F.hepatica* | N | N | N | N | N | / | 42 | 10 | 2 | Low |
| Carnevale et al (2013) [76] | Human | / | Argentina | *F.hepatica* | Y | N | N | Y | / | Y | 42 | 1 | 3 | Middle |
| Carnevale et al (2015) [77] | Sheep | / | Argentina | *F.hepatica* | N | N | N | N | N | / | 70 | 18 | 2 | Low |
| Carolina et al (2011) [78] | Human | / | Peru | *F.hepatica* | Y | Y | Y | Y | / | N | 476 | 116 | 4 | High |
| Casadiegos et al (2022) [79] | Cattle | 2016 | Colombia | *F.hepatica* | Y | N | N | N | N | / | 3943 | 188 | 5 | High |

| | | | | | | | | | | | | | | |
|---|---|---|---|---|---|---|---|---|---|---|---|---|---|---|
| Çelik et al (2018) [80] | Sheep | 2018 | Turkey | *F.hepatica* | N | Y | N | N | N | / | 320 | 24 | 4 | High |
| Çelik et al (2019) [81] | Cattle | 2017 | Turkey | *F.hepatica* | N | N | Y | N | N | / | 380 | 27 | 4 | High |
| Cengiz et al (2015) [82] | Human | 2008-2010 | Turkey | *F.hepatica* | N | N | Y | Y | / | N | 1600 | 89 | 5 | High |
| Chakraborty et al (2015) [83] | Cattle | 2011 | Bangladesh | / | N | N | Y | Y | N | / | 283 | 42 | 3 | Middle |
| Chanie et al (2012) [84] | Sheep | 2010-2011 | Ethiopia | / | Y | Y | N | Y | N | / | 621 | 436 | 5 | High |
| Chaouadi et al (2019) [85] | Cattle | 2016-2017 | Algeria | *F.hepatica* | N | N | Y | Y | N | / | 1400 | 85 | 5 | High |
| Chaparro et al (2016) [86] | Cattle | 2014 | Colombia | *F.hepatica* | Y | N | N | N | N | / | 911 | 282 | 3 | Middle |
| Chavez-Arce et al (2021) [87] | Cattle | 2018 | Peru | *F.hepatica* | Y | Y | N | N | N | / | 137 | 58 | 4 | High |
| Chen et al (2015) [88] | Human | 2014 | China | *F.giganticaa* | N | N | Y | Y | / | N | 1207 | 36 | 5 | High |
| Choubisa et al (2013) [89] | Cattle | 2010-2011 | India | *F.hepatica* | N | N | N | N | N | / | 130 | 24 | 3 | Middle |
| | Sheep | 2010-2011 | India | *F.hepatica* | N | N | N | N | N | / | 177 | 37 | 3 | Middle |
| Coelho et al (2021) [90] | Sheep | 2015-2016 | Portugal | *F.hepatica* | N | Y | N | N | N | / | 92 | 18 | 3 | Middle |
| Cornejo et al (2003) [91] | Human | / | Peru | / | N | N | N | N | / | N | 200 | 27 | 2 | Low |
| Correa et al (2016) [92] | Cattle | 2013 | Colombia | *F.hepatica* | N | N | N | N | N | / | 180 | 24 | 3 | Middle |
| Cuervo et al (2015) [93] | Sheep | 2006-2011 | Argentina | *F.hepatica* | N | N | N | N | N | / | 663 | 48 | 3 | Middle |
| Cuervo et al (2012) [94] | Sheep | 2006-2011 | Argentina | *F.hepatica* | N | N | N | N | N | / | 663 | 127 | 3 | Middle |
| Curtale et al (2003) [95] | Human | 2000 | Egypt | / | N | N | N | N | / | N | 1331 | 72 | 3 | Middle |
| Da Cunha et al (2007) [96] | Sheep | 2000-2005 | Brazil | *F.hepatica* | N | N | N | N | N | / | 690401 | 61239 | 3 | Middle |
| das Chagas Bernardo et al (2011) [97] | Cattle | 2006-2009 | Brazil | *F.hepatica* | N | N | N | N | N | / | 110956 | 27625 | 3 | Middle |
| Das et al (2018) [98] | Cattle | 2012-2013 | India | / | Y | N | N | N | N | / | 2339 | 11 | 2 | Low |
| Davoodi et al (2022) [99] | Human | 2018-2019 | Iran | *F.hepatica* | N | N | Y | Y | / | N | 2418 | 60 | 4 | High |
| de Alegría et al (2017) [100] | Human | 2015 | Angola | *F.hepatica* | N | N | N | N | / | N | 230 | 1 | 3 | Middle |
| de Aquino et al (2018) [101] | Cattle | 2007-2014 | Brazil | *F.hepatica* | N | N | N | N | N | / | 23255979 | 979 | 3 | Middle |

| | | | | | | | | | | | | | | |
|---|---|---|---|---|---|---|---|---|---|---|---|---|---|---|
| Degheidy and Al-Malki (2012) [102] | Cattle | 2009-2011 | Saudi Arabia | / | N | N | N | N | N | / | 57593 | 4951 | 4 | High |
| Degheidy and Al-Malki (2013) [103] | Sheep | 2010-2012 | Saudi Arabia | / | N | N | N | N | N | / | 18925 | 593 | 4 | High |
| Denizhan and Bicek (2018) [104] | Sheep | 2009 | Turkey | *F.hepatica* | N | N | Y | N | N | / | 352 | 174 | 3 | Middle |
| Dey et al (2022) [105] | Sheep | 2017-2018 | Bangladesh | / | Y | N | N | N | N | / | 2440 | 184 | 4 | High |
| Diaz-Quevedo et al (2021) [106] | Cattle | 2020 | Peru | *F.hepatica* | N | N | N | N | N | / | 790 | 712 | 3 | Middle |
| Dixit et al (2017) [107] | Sheep | 2012-2015 | India | *F.giganticaa* | N | Y | Y | N | N | / | 632 | 2 | 4 | High |
| Diyana et al (2019) [108] | Cattle | 2007-2017 | Malaysia | / | N | N | N | N | N | / | 1988 | 35 | 3 | Middle |
| Dracz and Lima (2014) [109] | Cattle | / | Brazil | *F.hepatica* | N | N | N | N | N | / | 74 | 21 | 1 | Low |
| El Damaty et al (2018) [110] | Cattle | 2016-2017 | Egypt | / | N | N | Y | Y | Y | / | 120 | 25 | 4 | High |
| Elelu et al (2016) [111] | Cattle | 2013 | Nigeria | *F.giganticaa* | Y | Y | N | N | N | / | 686 | 514 | 4 | High |
| Elliott et al (2015) [112] | dairy Cattle | 2013 | Australia | *F.hepatica* | N | N | N | N | N | / | 150 | 145 | 3 | Middle |
| Elmonir et al (2021) [113] | Human | 2018 | Egypt | *F.hepatica* | N | N | Y | Y | / | Y | 996 | 5 | 5 | High |
| Elshahawy et al (2014) [114] | Sheep | 2010-2011 | Egypt | *F.giganticaa* | N | N | N | N | N | / | 45 | 2 | 3 | Middle |
| Elshraway et al (2017) [115] | Cattle | 2016 | Egypt | / | N | N | N | Y | N | / | 2212 | 682 | 3 | Middle |
| El-Tahawy et al (2018) [116] | Cattle | 2015-2016 | Egypt | *F.hepatica* | N | N | Y | Y | N | / | 3356 | 432 | 5 | High |
| El-Tahawy et al (2017) [117] | Cattle | 2015 | Egypt | / | N | N | Y | Y | N | / | 4976 | 486 | 5 | High |
| Ernest and Robertson (2016) | Cattle | 2001-2010 | Botswana | *F.giganticaa* | N | N | N | N | N | / | 1394721 | 1249 | 3 | Middle |

| | | | | | | | | | | | | | | |
|---|---|---|---|---|---|---|---|---|---|---|---|---|---|---|
| [118] | | | | | | | | | | | | | | |
| Eshrati et al (2020) [119] | Human | 2016-2017 | Iran | / | N | N | N | Y | / | N | 1053 | 28 | 4 | High |
| Esteban et al (2003) [120] | Human | 2000 | Egypt | *F.hepatica* | N | N | Y | Y | / | N | 678 | 87 | 4 | High |
| Ezatpour et al (2015) [121] | Sheep | 2010-2013 | Iran | / | N | N | N | N | N | / | 265692 | 18931 | 3 | Middle |
| Ezatpour et al (2014) [122] | Cattle | 2010-2013 | Iran | *F.hepatica* | Y | Y | N | N | N | / | 150869 | 11502 | 4 | High |
| Feichtenschlager et al (2014) [123] | Cattle | 2012-2013 | Pakistan | *F.hepatica* | N | N | Y | Y | N | / | 237 | 64 | 5 | High |
| Fentie et al (2013) [124] | Human | 2007-2008 | Ethiopia | / | Y | Y | N | Y | / | N | 520 | 17 | 5 | High |
| Fernandes et al (2019) [125] | Cattle | 2008-2011 | Brazil | *F.hepatica* | N | N | N | N | N | / | 20635 | 1422 | 3 | Middle |
| Florencia Ojeda et al (2020) [126] | Cattle | 2014 | Mexico | / | N | Y | N | N | N | / | 1078 | 278 | 4 | High |
| Forero et al (2016) [127] | Cattle | 2013 | Colombia | *F.hepatica* | Y | N | N | N | N | / | 142 | 56 | 4 | High |
| Founta et al (2018) [128] | Cattle | 2014 | Greece | *F.hepatica* | N | N | N | N | N | / | 110 | 18 | 4 | High |
| Freites et al (2009) [129] | Human | / | Venezuela | *F.hepatica* | N | Y | N | N | / | N | 51 | 0 | 2 | Low |
| Fromsa et al (2011) [130] | Cattle | / | / | / | Y | Y | N | N | N | / | 583 | 264 | 2 | Low |
| Gao et al (2020) [131] | Sheep | 2012-2017 | China | *F.hepatica* | N | N | Y | Y | N | / | 1092 | 405 | 4 | High |
| | Cattle | 2012-2017 | China | *F.hepatica* | N | N | Y | Y | N | / | 3276 | 1452 | 3 | Middle |
| Garg et al (2009) [132] | Cattle | 2001-2004 | India | / | N | Y | N | N | N | / | 22835 | 2465 | 3 | Middle |
| | Sheep | 2001-2004 | India | / | N | Y | N | N | N | / | 22304 | 566 | 3 | Middle |
| Gauta et al (2011) [133] | Cattle | 2006 | Venezuela | *F.hepatica* | Y | N | N | N | N | / | 101 | 24 | 4 | High |
| Gebeyehu et al (2013) [134] | Sheep | 2009-2011 | Korea | *F.hepatica* | N | Y | Y | N | N | / | 464 | 5 | 4 | High |
| Gebrecherkos and Afera (2012) [135] | Cattle | / | Ethiopia | *Fasciola* | Y | Y | N | Y | N | / | 522 | 112 | 4 | High |
| Getahun et al (2020) [136] | Cattle | 2005-2018 | Ethiopia | / | N | N | N | N | N | / | 51956 | 9877 | 3 | Middle |
| | Sheep | 2005-2018 | Ethiopia | / | N | N | N | N | N | / | 6831 | 674 | 2 | Middle |
| Ghalandarzadeh and | Cattle | 2016 | Iran | / | N | N | N | Y | N | / | 316 | 40 | 3 | Middle |

| | | | | | | | | | | | | | | |
|---|---|---|---|---|---|---|---|---|---|---|---|---|---|---|
| Amniattalab (2019) [137] | | | | | | | | | | | | | | |
| Gharekhani et al (2015) [138] | Sheep | 2010-2011 | Iran | *F.hepatica* | N | Y | N | N | N | / | 100 | 13 | 4 | High |
| Ghazani et al (2008) [139] | Sheep | 2004 | Iran | *F.hepatica* | N | N | Y | N | N | / | 140 | 12 | 4 | High |
| Gherroucha et al (2021) [140] | Cattle | 2009-2018 | Algeria | *F.hepatica* | N | N | N | N | N | / | 145919 | 4005 | 3 | Middle |
| | Sheep | 2009-2018 | Algeria | *F.hepatica* | N | N | N | N | N | / | 345289 | 817 | 3 | Middle |
| Gherroucha et al (2022) [141] | Cattle | 2018-2019 | Algeria | *F.hepatica* | N | N | N | N | N | / | 1036 | 20 | 4 | High |
| | Sheep | 2018-2019 | Algeria | *F.hepatica* | N | N | N | N | N | / | 2574 | 1 | 4 | High |
| Gillandt et al (2018) [142] | Cattle | 2015-2016 | Germany | *F.hepatica* | N | N | N | N | N | / | 708 | 93 | 3 | Middle |
| Giraldo Pinzón et al (2016) [143] | Cattle | 2012 | Colombia | *F.hepatica* | N | N | N | N | N | / | 414 | 79 | 4 | High |
| Gordon et al (2015) [144] | Cattle | 2011 | Philippines | *F.giganticaa* | N | N | Y | Y | N | / | 153 | 95 | 4 | High |
| Goz et al (2014) [145] | Sheep | 2010 | Turkey | *F.hepatica* | N | N | N | N | N | / | 544 | 145 | 2 | Low |
| Gupta et al (2008) [145] | Cattle | 2000-2004 | India | *F.giganticaa* | N | N | N | N | N | / | 370 | 40 | 3 | Middle |
| Hamiroune et al (2020) [146] | Cattle | 2017-2018 | Algeria | *F.hepatica* | N | N | N | N | N | / | 5587 | 447 | 3 | Middle |
| | Sheep | 2017-2018 | Algeria | *F.hepatica* | N | N | N | N | N | / | 933 | 4 | 3 | Middle |
| Hammami and Ayadi (2007) [147] | Human | 2004-2005 | Tunisia | *F.hepatica* | N | N | N | N | / | N | 30 | 2 | 2 | Low |
| Haymanot and Kaba (2022) [148] | cow | / | Ethiopia | / | N | N | N | N | N | / | 422 | 33 | 3 | Middle |
| Hernandez-Guzman et al (2021) [149] | Cattle | 2017 | Mexico | *F.hepatica* | N | N | N | N | N | / | 6834 | 1407 | 3 | Middle |
| Höglund et al (2010) [150] | cow | 2008 | Sweden | *F.hepatica* | N | N | N | N | N | / | 210 | 15 | 4 | High |

| | | | | | | | | | | | | | | |
|---|---|---|---|---|---|---|---|---|---|---|---|---|---|---|
| Hosseini et al (2015) [151] | Human | 2014 | Iran | / | N | Y | N | N | / | Y | 1025 | 2 | 4 | High |
| Ibáñez et al (2004) [152] | Human | 2001-2002 | Peru | *F.hepatica* | N | N | N | N | / | N | 1049 | 2 | 3 | Middle |
| Ikenna-Ezeh et al (2019) [153] | Cattle | 2008 | Nigeria | *F.giganticaa* | N | N | N | N | N | / | 200 | 47 | 4 | High |
| Imani Baran et al (2017) [154] | Cattle | 2014-2015 | Iran | *F.hepatica* | N | N | N | N | N | / | 230 | 10 | 3 | Middle |
| | Sheep | 2014-2015 | Iran | *F.hepatica* | N | N | N | N | N | / | 1740 | 752 | 3 | Middle |
| Innocent et al (2017) [155] | Cattle | 2007-2009 | Scotland | / | N | N | N | N | N | / | 7858 | 1751 | 3 | Middle |
| Isah (2019) [156] | Cattle | 2017-2018 | Nigeria | / | N | Y | N | Y | N | / | 3560 | 1628 | 3 | Middle |
| | Sheep | 2017-2018 | Nigeria | / | N | Y | N | Y | N | / | 4080 | 1461 | 3 | Middle |
| Jajaa et al (2020) [157] | Cattle | 2013-2014 | / | / | N | N | N | N | N | / | 1120 | 270 | 4 | High |
| Japa et al (2020) [158] | Cattle | 2019 | Thailand | / | N | N | Y | Y | N | / | 311 | 32 | 3 | Middle |
| Jean-Richard et al (2014) [159] | Sheep | 2011 | Chad | *F.giganticaa* | N | N | N | N | Y | / | 616 | 80 | 3 | Middle |
| | Cattle | 2011 | Chad | *F.giganticaa* | N | N | N | N | Y | / | 130 | 89 | 3 | Middle |
| Jimenez-Rocha et al (2017) [160] | Cattle | 2008 | Costa Rica | *F.hepatica* | Y | Y | N | N | N | / | 526 | 19 | 4 | High |
| Kabir et al (2009) [161] | Cattle | 2008 | Bangladesh | / | N | N | Y | Y | N | / | 1460 | 398 | 3 | Middle |
| | Sheep | 2008 | Bangladesh | / | N | N | Y | Y | N | / | 1430 | 268 | 3 | Middle |
| Kadir et al (2012) [162] | Sheep | 2009-2010 | Iraq | / | N | N | N | N | N | / | 50518 | 183 | 3 | Middle |
| | Cattle | 2009-2010 | Iraq | / | N | N | N | N | N | / | 16225 | 207 | 3 | Middle |
| Kakar and Kakarsulemankhel (2008) [163] | Cattle | 2005-2006 | Pakistan | *Fasciola* | N | N | N | N | N | / | 736 | 158 | 4 | High |
| Kantzoura et al (2011) [164] | Sheep | 2006-2007 | Greece | / | N | Y | N | N | N | / | 346 | 39 | 3 | Middle |
| Kara et al (2009) [165] | Sheep | 2008 | Turkey | *F.hepatica* | N | N | Y | N | N | / | 1763 | 78 | 3 | Middle |

| | | | | | | | | | | | | | | |
|---|---|---|---|---|---|---|---|---|---|---|---|---|---|---|
| | Sheep | 2008 | Turkey | *F.hepatica* | N | N | Y | Y | N | / | 513 | 28 | 3 | Middle |
| Karahocagil et al (2011) [166] | Human | / | Turkey | *F.hepatica* | N | N | N | N | / | N | 92 | 11 | 1 | Low |
| Karapinar et al (2012) [167] | Sheep | 2008-2009 | Turkey | / | Y | N | Y | N | N | / | 201 | 13 | 4 | High |
| Kato et al (2005) [168] | Cattle | 2002 | Japan | / | N | N | N | N | N | / | 6224 | 172 | 3 | Middle |
| Katsoulos et al (2011) [169] | Sheep | / | / | *F.hepatica* | N | N | N | N | N | / | 254 | 68 | 3 | Middle |
| Kedir et al (2012) [170] | Sheep | 2011-2012 | Ethiopia | / | N | Y | Y | Y | N | / | 384 | 56 | 4 | High |
| Khan et al (2022) [171] | Human | 2016 | Pakistan | / | N | Y | N | N | / | N | 296 | 1 | 3 | Middle |
| Khan et al (2009) [172] | Cattle | 2006-2007 | Pakistan | / | N | N | N | N | Y | / | 4800 | 1222 | 4 | High |
| Khan et al (2011) [173] | Cattle | 2008-2009 | Pakistan | *F.hepatica* | N | Y | N | N | N | / | 1200 | 239 | 5 | High |
| Khan et al (2010) [174] | Cattle | 2008 | Pakistan | *Fasciola* | N | Y | Y | Y | Y | / | 1140 | 281 | 5 | High |
| | Sheep | 2008 | Pakistan | *Fasciola* | N | Y | Y | Y | Y | / | 1500 | 513 | 5 | High |
| Khanjari et al (2014) [175] | Sheep | 2011 | Iran | / | N | N | N | Y | N | / | 2391 | 157 | 3 | Middle |
| Kheder and Mohamed (2021) [176] | Cattle | 2009-2011 | Sudan | / | N | Y | N | N | N | / | 234175 | 4303 | 3 | Middle |
| Khedri et al (2021) [177] | Cattle | 2008-2016 | Iran | / | N | N | N | N | N | / | 857039 | 27253 | 3 | Middle |
| Kheirandish et al (2016) [178] | Human | 2014 | Iran | / | N | N | Y | Y | / | Y | 801 | 6 | 4 | High |
| Khoramian et al (2014) [179] | Cattle | 2012-2013 | Iran | / | N | N | N | N | N | / | 10462 | 385 | 3 | Middle |
| | Sheep | 2012-2013 | Iran | / | N | N | N | N | N | / | 240863 | 7120 | 3 | Middle |
| Kim et al (2008) [180] | Cattle | 2007 | Korea | / | N | N | N | N | N | / | 1000 | 12 | 3 | Middle |
| Kipyegen et al (2022) [181] | Human | 2016-2017 | Kenya | / | N | Y | N | N | / | N | 272 | 0 | 3 | Middle |
| | Cattle | 2016-2017 | Kenya | / | N | Y | N | N | N | / | 268 | 90 | 3 | Middle |
| | Sheep | 2016-2017 | Kenya | / | N | Y | N | N | N | / | 268 | 90 | 3 | Middle |
| Kiswaga et al (2016) [182] | Cattle | 2015-2016 | Tanzania | / | N | N | N | N | N | / | 1648 | 51 | 2 | Middl |

| | | | | | | | | | | | | | | |
|---|---|---|---|---|---|---|---|---|---|---|---|---|---|---|
| | | | | | | | | | | | | | | e |
| | | | | | | | | | | | | | | Middl |
| | Sheep | 2015-2016 | Tanzania | / | N | N | N | N | N | / | 2414 | 20 | 2 | e |
| | | | | | | | | | | | | | | Middl |
| Kiziewicz(2013) [183] | Cattle | 2001-2006 | Poland | *F.hepatica* | N | N | N | N | N | / | 178 | 63 | 3 | e |
| Kohi et al (2017) [184] | Cattle | 2015 | Iran | / | N | N | N | N | N | / | 4474 | 642 | 3 | Middle |
| | Sheep | 2015 | Iran | / | N | N | N | N | N | / | 40727 | 904 | 3 | Middle |
| Kordshooli et al (2017) [185] | Sheep | 2011-2015 | Iran | / | Y | Y | N | N | N | / | 44191 | 2310 | 4 | High |
| | Cattle | 2011-2015 | Iran | / | Y | Y | N | N | N | / | 12079 | 1347 | 4 | High |
| Kouadio et al (2020) [186] | Cattle | 2018 | Côte d'Ivoire | *F.giganticaa* | N | N | Y | Y | N | / | 1121 | 304 | 4 | High |
| | Sheep | 2018 | Côte d'Ivoire | *F.giganticaa* | N | N | Y | Y | N | / | 523 | 20 | | |
| Kouam et al (2019) [187] | Cattle | 2016 | Cameroon | / | N | Y | N | N | N | / | 1472 | 333 | 4 | High |
| Kowalczyk et al (2018) [188] | Cattle | 2013 | Poland | *F.hepatica* | N | N | N | N | N | / | 203 | 47 | 3 | Middle |
| Kozlowska-Loj and Loj-Maczulska (2013) [189] | Cattle | 2009-2012 | Poland | *F.hepatica* | N | N | N | N | N | / | 542943 | 64540 | 3 | Middle |
| Kozlowska-Loj (2011) [190] | Cattle | 2005-2008 | Poland | *F.hepatica* | N | N | N | N | N | / | 335116 | 71180 | 3 | Middle |
| Krishna and D'Souza (2015) [191] | Cattle | / | India | / | N | N | N | N | N | / | 130 | 7 | 3 | Middle |
| Kuchai et al (2011) [192] | Cattle | 2011 | Egypt | / | N | N | Y | Y | N | / | 208 | 70 | 4 | High |
| Kuerpick et al (2013) [193] | cow | 2008 | Germany | *F.hepatica* | N | Y | N | N | N | / | 19947 | 4712 | 4 | High |
| Kumar et al (2016) [194] | Cattle | 2014 | India | / | N | N | N | N | N | / | 88 | 1 | 2 | Low |
| Kurnianto et al (2022) [195] | Cattle | 2020 | Indonesia | / | Y | Y | Y | Y | Y | / | 400 | 66 | 5 | High |
| Kusumarini et al (2020) [196] | Cattle | 2019 | Indonesia | / | N | N | N | N | N | / | 265 | 79 | 3 | Middle |

| | | | | | | | | | | | | | | |
|---|---|---|---|---|---|---|---|---|---|---|---|---|---|---|
| | Sheep | 2019 | Indonesia | / | N | N | N | N | N | / | 1007 | 60 | | |
| Lambacher et al (2020) [197] | Sheep | 2017 | Austria | / | N | N | N | N | N | / | 218 | 0 | 2 | Low |
| Latchumikanthan and Soundararajan (2013) [198] | Cattle | 2009-2010 | India | *F.giganticaa* | N | N | N | N | N | / | 168 | 25 | 3 | Middle |
| Liba et al (2017) [199] | Cattle | 2015-2016 | Nigeria | / | Y | N | N | N | N | / | 300 | 36 | 4 | High |
| | Sheep | 2015-2016 | Nigeria | / | Y | N | N | N | N | / | 600 | 49 | 4 | High |
| Lino Zumaquero-Rios et al (2013) [200] | Human | / | Mexico | / | Y | N | Y | Y | / | N | 865 | 14 | 4 | High |
| Livia-Córdova et al (2021) [201] | Cattle | 2015-2016 | Peru | *F.hepatica* | Y | Y | Y | N | N | / | 360 | 153 | 4 | High |
| Lopez et al (2012) [202] | Human | 2010 | Peru | *F.hepatica* | Y | N | N | N | / | N | 223 | 23 | 3 | Middle |
| Lukambagire et al (2015) [203] | Human | 2013 | Tanzania | / | N | N | N | N | / | N | 1460 | 305 | 3 | Middle |
| Maciel et al (2018) [204] | Human | 2013 | Brazil | / | Y | Y | Y | Y | / | N | 434 | 36 | 4 | High |
| Malathi et al (2021) [205] | Cattle | 2019 | India | / | N | N | N | N | N | / | 595 | 42 | 3 | Middle |
| | Sheep | 2019 | India | / | N | N | N | N | N | / | 2447 | 43 | 3 | Middle |
| Manouchehri Naeini et al (2016) [206] | Human | 2013-2015 | Iran | *F.hepatica* | N | N | Y | Y | / | Y | 1475 | 2 | 4 | High |
| Mantari et al (2012) [207] | Human | 2009 | Peru | *F.hepatica* | N | N | Y | Y | / | N | 312 | 16 | 3 | Middle |
| Maqbool et al (2018) [208] | Cattle | 2014-2015 | India | / | N | N | N | N | N | / | 1163 | 24 | 3 | Middle |
| Marcos et al (2005) [209] | Human | / | Peru | *F.hepatica* | N | N | Y | N | / | N | 93 | 83 | 1 | Low |
| Marcos et al (2007) [210] | Human | 2002-2003 | Peru | *F.hepatica* | Y | N | N | N | / | N | 102 | 4 | 4 | High |
| Marcos Raymundo et al (2004) [211] | Human | / | Peru | *F.hepatica* | Y | N | Y | N | / | N | 194 | 40 | 3 | Middle |
| Marskole et al (2016) [212] | Cattle | 2014-2015 | India | *F.giganticaa* | Y | Y | N | N | N | / | 76 | 5 | 3 | Middle |

| | | | | | | | | | | | | | | |
|---|---|---|---|---|---|---|---|---|---|---|---|---|---|---|
| Mas-Coma et al (2020) [213] | Sheep | / | Bolivia | *F.hepatica* | Y | N | N | N | N | / | 1202 | 759 | 3 | Middle |
| | Cattle | / | Bolivia | *F.hepatica* | Y | N | N | N | N | / | 2690 | 554 | 3 | Middle |
| May et al (2019) [214] | cow | 2005-2006 | Germany | *F.hepatica* | N | N | N | N | N | / | 1043 | 95 | 3 | Middle |
| Meguini et al (2021) [215] | Cattle | / | Algeria | *F.hepatica* | N | N | N | N | N | / | 530 | 65 | 2 | Middle |
| Meharenet and Shitu (2021) [216] | Cattle | 2016-2017 | Ethiopia | / | Y | Y | N | Y | N | / | 520 | 147 | 5 | High |
| Mellau et al (2011) [217] | Cattle | 2005-2007 | Tanzania | / | N | N | Y | N | N | / | 115186 | 9903 | 3 | Middle |
| | Sheep | 2005-2007 | Tanzania | / | N | N | Y | N | N | / | 99401 | 3097 | 3 | Middle |
| Mendes et al (2019) [218] | Cattle | 2008-2011 | Brazil | *F.hepatica* | N | N | N | N | N | / | 20635 | 1422 | 3 | Middle |
| Mensur et al (2016) [219] | Sheep | 2013-2014 | Ethiopia | / | Y | Y | Y | Y | Y | / | 384 | 46 | 4 | High |
| Mimoune et al (2022) [220] | Cattle | / | Algeria | / | N | N | N | N | N | / | 1756 | 67 | 2 | Middle |
| Mochankana and Robertson (2016) [221] | Cattle | 2001-2010 | Botswana | *F.giganticaa* | N | N | N | N | N | / | 2789442 | 2500 | 3 | Middle |
| Mochankana and Robertson (2018) [222] | Cattle | 2011-2013 | Botswana | *F.giganticaa* | N | N | N | Y | N | / | 8646 | 64 | 4 | High |
| Mohamadzadeh et al (2016) [223] | Sheep | 2011-2013 | Iran | / | N | N | N | N | N | / | 104140 | 56 | 3 | Middle |
| | Cattle | 2011-2013 | Iran | / | N | N | N | N | N | / | 29988 | 545 | 3 | Middle |
| Molento et al (2020) [224] | Cattle | / | Brazil | *F.hepatica* | Y | N | N | N | N | / | 12236 | 1904 | 2 | Middle |
| Molla et al (2019) [225] | Cattle | 2005-2014 | Ethiopia | / | Y | Y | N | N | N | / | 46913 | 5853 | 4 | High |
| Monteiro et al (2013) [226] | Cattle | 2008-2011 | Angola | / | N | N | N | N | N | / | 16192 | 2698 | 3 | Middle |
| Montiel et al (2001) [227] | Cattle | / | Venezuela | *F.hepatica* | N | Y | N | N | N | / | 731 | 250 | 4 | High |
| Moussouni et al (2018) [228] | Cattle | 2013-2014 | Algeria | *F.hepatica* | Y | Y | N | N | N | / | 143 | 18 | 4 | High |
| Mulatu and Addis (2011) | Sheep | 2010-2011 | Ethiopia | / | Y | Y | N | N | N | / | 768 | 89 | 4 | High |

| | | | | | | | | | | | | | | |
|---|---|---|---|---|---|---|---|---|---|---|---|---|---|---|
| [229] | and Sheep | | | | | | | | | | | | | |
| Mungube et al (2012) [230] | Cattle | 2009 | Kenya | / | N | Y | Y | Y | N | / | 197 | 51 | 4 | High |
| | Sheep | 2009 | Kenya | / | N | Y | Y | N | N | / | 141 | 36 | 4 | High |
| Munyeme et al (2012) [231] | Cattle | 2008 | Zambia | / | N | Y | N | N | N | / | 1680 | 600 | 3 | Middle |
| Murthy and Rao (2014) [232] | Cattle | / | India | / | N | N | N | N | N | / | 150 | 8 | 2 | Middle |
| Nahed-Toral et al (2003) [233] | Sheep | / | Mexico | *F.hepatica* | Y | Y | N | N | Y | / | 312 | 117 | 3 | Middle |
| Najib et al (2020) [234] | Human | 2017-2018 | Malaysia | / | N | N | Y | Y | / | N | 90 | 60 | 2 | Middle |
| Nasreldin and Zaki (2020) [235] | Cattle | 2018 | Egypt | *F.hepatica* | N | N | N | N | N | / | 226 | 38 | 3 | Middle |
| Nath et al (2015) [236] | Cattle | 2012-2013 | India | *F.giganticaa* | Y | N | N | N | N | / | 459 | 1 | 3 | Middle |
| | Sheep | 2012-2013 | India | *F.giganticaa* | Y | N | N | N | N | / | 441 | 1 | 3 | Middle |
| Nga Thi et al (2017) [237] | Cattle | 2014-2015 | Vietnam | / | N | N | N | Y | N | / | 572 | 134 | 4 | High |
| Nguyen et al (2016) [238] | Human | 2012 | Vietnam | / | N | N | Y | Y | / | N | 10084 | 590 | 3 | Middle |
| Nguyen et al (2012) [239] | Cattle | 2008-2009 | Vietnam | / | Y | N | Y | N | N | / | 1075 | 487 | 5 | High |
| Nguyen et al (2011) [240] | Cattle | 2008 | Vietnam | / | Y | Y | Y | N | N | / | 825 | 453 | 5 | High |
| Nicoletti et al (2022) [241] | Cattle | 2018 | Peru | *F.hepatica* | Y | Y | Y | N | N | / | 295 | 150 | 3 | Middle |
| Nnabuife et al (2013) [242] | Cattle | 2011 | Nigeria | *F.gigantica* | N | N | N | N | N | / | 1525 | 37 | 4 | High |
| Novobilsky et al (2015) [243] | Cattle | 2006-2007 | Sweden | *F.hepatica* | N | N | N | N | N | / | 2767 | 263 | 3 | Middle |
| Novobilsky et al (2015) [244] | Cattle | 2012 | Sweden | *F.hepatica* | N | N | N | N | N | / | 426 | 107 | 4 | High |
| Nuñez et al (2017) [245] | Cattle | 2017 | Paraguay | *F.hepatica* | N | N | N | | | / | 4179 | 24 | 3 | Middle |

| | | | | | | | | | | | | | | |
|---|---|---|---|---|---|---|---|---|---|---|---|---|---|---|
| Nurhidayah et al (2020) [246] | Cattle | 2017-2018 | Indonesia | / | N | N | Y | Y | N | / | 580 | 93 | 4 | High |
| Nyirenda et al (2019) [247] | Cattle | 2013-2017 | Zambia | / | N | N | N | N | N | / | 69152 | 44511 | 3 | Middle |
| Obi et al (2020) [248] | Cattle | 2017 | Nigeria | / | N | N | N | N | | / | 210 | 43 | 4 | High |
| Ojeda-Robertos et al (2016) [249] | Cattle | 2019 | Mexico | *F.hepatica* | Y | Y | Y | Y | Y | / | 94 | 5 | 3 | Middle |
| Okajima et al (2016) [250] | Cattle | 2009-2012 | Japan | / | N | N | N | N | N | / | 378153 | 235 | 3 | Middle |
| Ola-Fadunsin et al (2020) [251] | Cattle | 2003-2018 | Nigeria | / | N | N | N | N | N | / | 832001 | 6371 | 3 | Middle |
| Ola-Fadunsin et al (2020) [252] | Cattle | 2018-2019 | Nigeria | *Fasciola* | N | Y | N | N | N | / | 478 | 82 | 5 | High |
| Oljira et al (2022) [253] | Sheep | 2020 | Ethiopia | / | Y | N | Y | Y | N | / | 424 | 88 | 5 | High |
| Opara (2005) [254] | Cattle | 2001 | Nigeria | *F.gigantica* | N | N | N | Y | N | / | 612 | 291 | 3 | Middle |
| Opio et al (2021) [255] | Cattle | 2016 | Uganda | / | N | N | Y | Y | N | / | 216 | 142 | 4 | High |
| Ortiz-Pineda et al (2021) [256] | Cattle | / | Colombia | *F.hepatica* | Y | N | N | Y | N | / | 343 | 102 | 3 | Middle |
| Oryan et al (2011) [257] | Cattle | 2008-2009 | Iran | / | N | N | N | N | N | / | 4933 | 35 | 3 | Middle |
| | Sheep | 2008-2009 | Iran | / | N | N | N | N | N | / | 34592 | 104 | 3 | Middle |
| Ouchene-Khelifi et al (2018) [258] | Cattle | 2008-2009 | Algeria | *F.hepatica* | N | Y | N | N | N | / | 5608 | 963 | 4 | High |
| | Sheep | 2008-2009 | Algeria | *F.hepatica* | N | Y | N | N | N | / | 11925 | 547 | 4 | High |
| Ozturhan et al (2009) [259] | Human | / | Turkey | *F.hepatica* | N | N | N | Y | / | N | 884 | 7 | 3 | Middle |
| Palacio Collado et al (2020) [260] | Cattle | 2012-2018 | Cuba | *F.hepatica* | N | N | N | N | N | / | 363053 | 104097 | 3 | Middle |
| Palacio Collado et al (2017) [261] | Cattle | 2008-2014 | Cuba | *F.hepatica* | N | N | N | N | N | / | 13059 | 2387 | 3 | Middle |

| | | | | | | | | | | | | | | |
|---|---|---|---|---|---|---|---|---|---|---|---|---|---|---|
| Paltin et al (2020) [262] | Cattle | 2018 | Romania | *F.hepatica* | Y | Y | Y | N | N | / | 105 | 22 | 5 | High |
| Pandya et al (2015) [263] | Cattle | 2013-2014 | India | / | N | N | N | N | N | / | 1552 | 314 | 3 | Middle |
| Paz-Silva et al (2003) [264] | Sheep | 2001 | Spain | *F.hepatica* | N | N | N | N | N | / | 402 | 157 | 4 | High |
| Pereira et al (2020) [265] | Cattle | 2004-2008,2010 | Brazil | *F.hepatica* | N | N | N | N | N | / | 518635 | 24455 | 3 | Middle |
| Perez et al (2016) [266] | Human | / | Colombia | *F.hepatica* | Y | N | N | N | / | N | 111 | 7 | 2 | Middle |
| Perez-Creo et al (2016) [267] | Sheep | 2015 | Spain | *F.hepatica* | N | N | Y | Y | N | / | 603 | 137 | 4 | High |
| Petersson et al (2017) [268] | Cattle | 2012-2013 | Estonia | *F.hepatica* | N | N | Y | N | N | / | 2461 | 144 | 3 | Middle |
| Pezeshki et al (2018) [269] | Sheep | 2015-2018 | Iran | / | N | Y | Y | Y | Y | / | 571991 | 3599 | 3 | Middle |
| | Cattle | 2015-2018 | Iran | / | N | Y | Y | Y | Y | / | 80001 | 204 | 3 | Middle |
| Phalee and Wongsawad (2014) [270] | Cattle | 2010-2012 | Thailand | *F.gigantica* | N | N | N | N | N | / | 106 | 64 | 3 | Middle |
| Phiri et al (2005) [271] | Cattle | 2002 | Zambia | *F.gigantica* | N | N | N | N | N | / | 677 | 331 | 3 | Middle |
| Pilarczyk et al (2019) [272] | cow | / | Poland | *F.hepatica* | N | N | N | N | N | / | 143 | 0 | 2 | Middle |
| Pilarczyk et al (2021) [273] | Sheep | 2019 | Poland | *F.hepatica* | N | N | N | N | N | / | 158 | 2 | 3 | Middle |
| Pile E (2000) [274] | Human | / | Brazil | *F.hepatica* | N | N | N | N | / | N | 500 | 2 | 2 | Middle |
| Pinilla et al (2019) [275] | Cattle | 2018 | Colombia | *F.hepatica* | Y | Y | N | N | N | / | 196 | 8 | 4 | High |
| Pinilla et al (2020) [276] | Cattle | 2017-2019 | Colombia | *F.hepatica* | Y | Y | Y | Y | N | / | 103 | 54 | 5 | High |
| Pinilla et al (2018) [277] | Cattle | 2015-2016 | Colombia | *F.hepatica* | N | Y | Y | N | N | / | 862 | 30 | 4 | High |
| Pinilla et al (2020) [278] | Cattle | 2017-2018 | Colombia | *F.hepatica* | Y | N | Y | Y | N | / | 5620 | 358 | 4 | High |
| | Sheep | 2017-2018 | Colombia | *F.hepatica* | Y | N | Y | Y | N | / | 97 | 29 | 4 | High |
| Pinilla León et al (2019) [279] | Cattle | 2017-2018 | Colombia | *F.hepatica* | Y | Y | N | N | N | / | 103 | 23 | 5 | High |
| | Sheep | 2017-2018 | Colombia | *F.hepatica* | Y | Y | N | N | N | / | 97 | 14 | 5 | High |
| Pino Santos et al (2014) | Human | 2011-2012 | Cuba | *F.hepatica* | N | N | N | N | / | N | 300 | 1 | 4 | High |

| | | | | | | | | | | | | | | |
|---|---|---|---|---|---|---|---|---|---|---|---|---|---|---|
| [280] | | | | | | | | | | | | | | |
| Piri et al (2018) [281] | Cattle | 2015 | Iran | *Fasciola* | N | N | N | N | N | / | 995 | 15 | 3 | Middle |
| | Sheep | 2015 | Iran | *Fasciola* | N | N | N | N | N | / | 12612 | 85 | 3 | Middle |
| Poddar et al (2017) [282] | Sheep | 2016 | Bangladesh | *F.gigantica* | N | N | Y | Y | Y | / | 106 | 12 | 5 | High |
| Pratiwi et al (2019) [283] | Cattle | / | Indonesia | / | N | N | N | N | N | / | 100 | 27 | 2 | Middle |
| Pritsch et al (2019) [284] | Cattle | 2003-2017 | Brazil | *F.hepatica* | N | N | N | N | N | / | 226561 | 7187 | 3 | Middle |
| Pulido-Medellín et al (2014) [285] | Sheep | / | Colombia | *F.hepatica* | Y | N | N | N | N | / | 90 | 7 | 1 | Low |
| Quevedo et al (2018) [286] | Cattle | 2016-2017 | Brazil | *F.hepatica* | N | N | N | N | N | / | 210 | 79 | 3 | Middle |
| Quispe et al (2021) [287] | Human | 2013-2014 | Peru | / | Y | N | Y | Y | / | N | 295 | 5 | 4 | High |
| Qureshi et al (2012) [288] | Cattle | 2003-2005 | Pakistan | / | N | N | N | N | N | / | 7200 | 1058 | 4 | High |
| Qureshi et al (2016) [289] | Human | 2003-2005 | Pakistan | / | N | N | Y | Y | / | Y | 7200 | 85 | 4 | High |
| Qureshi et al (2019) [290] | Human | / | Pakistan | *F.hepatica* | Y | N | N | Y | / | Y | 540 | 4 | 4 | High |
| Radfar et al (2015) [291] | Cattle | 2011-2012 | Iran | *F.hepatica* | N | Y | Y | Y | Y | / | 680 | 137 | 4 | High |
| Rashid et al (2015) [292] | Cattle | 2013 | Bangladesh | / | N | N | Y | Y | N | / | 230 | 9 | 4 | High |
| Rassol et al (2020) [293] | Cattle | 2018-2019 | Egypt | / | N | N | N | Y | N | / | 81452 | 6516 | 3 | Middle |
| Raymundo et al (2004) [294] | Human | / | Peru | / | Y | N | N | N | / | N | 194 | 40 | 2 | Middle |
| Raza et al (2016) [295] | Cattle | 2009-2013 | Pakistan | *F.hepatica* | N | N | N | Y | N | / | 500 | 42 | 4 | High |
| Rinca et al (2019) [296] | Cattle | 2018 | Indonesia | / | N | N | N | N | | / | 100 | 48 | 3 | Middle |
| Rita et al (2017) [297] | Cattle | 2015-2016 | Malaysia | / | N | N | N | N | N | / | 219 | 49 | 4 | High |
| Rizwan et al (2016) [298] | Sheep | 2014 | Pakistan | *F.gigantica* | N | N | N | N | N | / | 3504 | 1496 | 4 | High |
| Rizwan et al (2021) [299] | Sheep | / | Pakistan | / | N | N | N | Y | N | / | 1200 | 128 | 2 | Middle |
| Rodriguez-Ulloa et al (2018) [300] | Human | 2014 | Peru | *F.hepatica* | Y | N | N | N | / | Y | 270 | 17 | 5 | High |
| Rojas-Araya et al (2016) | Cattle | 2014 | Costa Rica | / | N | N | N | N | N | / | 463 | 12 | 3 | Middle |

| | | | | | | | | | | | | | | |
|---|---|---|---|---|---|---|---|---|---|---|---|---|---|---|
| [301] | | | | | | | | | | | | | | |
| Roldan et al (2021) [302] | Cattle | 2008-2019 | Spain | *F.hepatica* | Y | Y | N | N | N | / | 137 | 79 | 4 | High |
| | Sheep | 2008-2019 | Spain | *F.hepatica* | Y | Y | N | N | N | / | 155 | 66 | 4 | High |
| Ruhoollah (2021) [303] | Sheep | / | Pakistan | *F.hepatica* | N | N | N | N | Y | / | 131 | 14 | 2 | Middle |
| Ruiz de Alegria et al (2017) [304] | Human | 2015 | Angola | *F.hepatica* | N | N | N | N | / | Y | 230 | 1 | 3 | Middle |
| Saad et al (2019) [305] | Cattle | / | Malaysia | / | N | N | N | N | N | / | 393 | 6 | 2 | Middle |
| Saberinasab et al (2014) [306] | Human | 2014 | Iran | / | N | N | Y | N | / | N | 471 | 8 | 4 | High |
| Sahin et al (2014) [307] | Human | 2005-2011 | Turkey | / | N | N | N | N | / | N | 2662 | 2 | 3 | Middle |
| Saltan and Taskin (2020) [308] | Cattle | 2018 | Turkey | *F.hepatica* | N | N | N | N | N | / | 200 | 63 | 3 | Middle |
| Samakkhah et al (2021) [309] | Cattle | 2018-2019 | Iran | / | N | N | N | N | N | / | 142403 | 2623 | 3 | Middle |
| | Sheep | 2018-2019 | Iran | / | N | N | N | N | N | / | 869507 | 17821 | 3 | Middle |
| Sanchez et al (2002) [310] | Cattle | / | Spain | / | N | N | Y | N | N | / | 1284 | 1083 | 3 | Middle |
| Sanchez and Lewis (2013) [311] | Cattle | 2005-2010 | Switzerland | / | N | N | N | N | N | / | 328 | 137 | 3 | Middle |
| Sarkari et al (2012) [312] | Human | / | Iran | / | N | N | Y | Y | / | N | 1000 | 18 | 2 | Middle |
| Sayeed et al (2020) [313] | Sheep | 2018-2019 | Bangladesh | / | N | Y | N | N | N | / | 612 | 2 | 3 | Middle |
| Secchioni et al (2016) [314] | Cattle | 2012-2014 | Italy | *F.hepatica* | N | N | N | N | N | / | 76 | 0 | 2 | Middle |
| | Sheep | 2012-2014 | Italy | *F.hepatica* | N | N | N | N | N | / | 102 | 0 | 2 | Middle |
| Sen et al (2011) [315] | Cattle | 2008-2009 | Turkey | *F.hepatica* | N | N | N | N | N | / | 198 | 6 | 3 | Middle |
| Shahatha et al (2021) [316] | Sheep | / | Iraq | *F.hepatica* | N | N | Y | Y | N | / | 455 | 198 | 3 | Middle |
| Shahnawaz et al (2011) [317] | Sheep | / | India | *F.giganticaa* | N | N | N | N | N | / | 55 | 7 | 1 | Low |

| | | | | | | | | | | | | | | |
|---|---|---|---|---|---|---|---|---|---|---|---|---|---|---|
| Shahzad et al (2012) [318] | Sheep | 2021 | Pakistan | *F.hepatica* | N | N | N | N | N | / | 400 | 32 | 3 | Middle |
| Shamsi et al (2020) [319] | Cattle | 2014-2016 | Iran | / | N | N | N | N | N | / | 1653 | 14 | 3 | Middle |
| | Sheep | 2014-2016 | Iran | / | N | N | N | N | N | / | 26720 | 18 | 3 | Middle |
| Shinggu et al (2019) [320] | Cattle | 2015 | Nigeria | / | N | Y | Y | Y | N | / | 262 | 74 | 4 | High |
| Shykat et al (2022) [321] | Sheep | 2018-2019 | Bangladesh | / | N | N | Y | Y | N | / | 260 | 92 | 3 | Middle |
| Silvia Paucar et al (2010) [322] | Cattle | 2006 | United states | *F.hepatica* | Y | Y | Y | N | N | / | 408 | 41 | 4 | High |
| Singh et al (2012) [323] | Cattle | 2004-2009 | India | / | N | N | N | N | N | / | 159 | 3 | 3 | Middle |
| Sreedevi and Hafeez (2014) [324] | Cattle | / | India | / | N | N | N | N | N | / | 694 | 14 | 2 | Middle |
| Sreedhar et al (2009) [325] | Cattle | / | India | / | N | | N | N | N | / | 800 | 30 | 2 | Middle |
| Steinmann et al (2010) [326] | Human | 2009 | Kyrgyzstan | *F.hepatica* | N | N | N | N | / | N | 1262 | 24 | 3 | Middle |
| Sun et al (2020) 327] | Cattle | 2016 | Rwanda | / | N | Y | N | N | Y | / | 569 | 113 | 4 | High |
| Swai and Ulicky (2009) [328] | Cattle | 2004-2005 | Tanzania | / | Y | Y | N | N | N | / | 2114 | 297 | 4 | High |
| Takang et al (2019) [329] | Cattle | 2010 | Cameroon | / | N | N | N | N | N | / | 767 | 138 | 4 | High |
| Takeuchi-Storm et al (2017) [330] | Cattle | 2013 | Denmark | *F.hepatica* | N | N | N | N | N | / | 194 | 106 | 3 | Middle |
| Tanabe et al (2021) [331] | Human | / | Peru | *F.hepatica* | N | N | Y | Y | / | N | 2630 | 148 | 2 | Middle |
| Taş Cengiz (2015) [332] | Human | 2008-2010 | Turkey | *F.hepatica* | N | N | Y | Y | / | N | 1600 | 29 | 5 | High |
| Tas Cengiz et al (2009) [333] | Human | 2003-2005 | Turkey | *F.hepatica* | N | N | Y | Y | / | N | 2975 | 1 | 4 | High |
| Tavakoli et al (2008) [334] | Sheep | 2003-2007 | Iran | / | N | N | N | N | N | / | 32714926 | 815807 | 3 | Middle |
| Teixeira et al (2023) [335] | Cattle | 2018-2021 | Brazil | / | N | N | N | N | N | / | 48202 | 4972 | 3 | Middle |
| Tembo and Nonga (2015) [336] | Cattle | 2010-2012 | Tanzania | / | N | Y | N | N | N | / | 85980 | 3903 | 3 | Middle |

| | | | | | | | | | | | | | | |
|---|---|---|---|---|---|---|---|---|---|---|---|---|---|---|
| Tesfaheywet and Negash (2012) [337] | Sheep | 2010-2011 | Ethiopia | / | Y | Y | Y | Y | N | / | 384 | 175 | 5 | High |
| Thanasuwan and Tankrathok (2021) [338] | Cattle | 2020 | Thailand | / | N | N | N | N | N | / | 46 | 16 | 2 | Middle |
| Thapa et al (2022) [339] | Cattle | 2019 | Nepal | / | N | N | N | N | N | / | 100 | 11 | 3 | Middle |
| Ticona et al (2010) [340] | Cattle | 2004 | Peru | *F.hepatica* | Y | Y | Y | Y | N | / | 381 | 136 | 4 | High |
| | Sheep | 2004 | Peru | *F.hepatica* | Y | Y | Y | Y | N | / | 207 | 81 | 4 | High |
| Toan et al (2016) [341] | Human | 2012 | Vietnam | / | N | N | N | N | / | N | 10084 | 590 | 3 | Middle |
| Tongjura et al (2019) [342] | Human | 2006 | Nigera | *F.hepatica* | Y | N | Y | N | / | N | 200 | 7 | 4 | High |
| Traore et al (2021) [343] | Cattle | 2014 | Côte d'Ivoire | *F.gigantica* | N | N | Y | Y | N | / | 246 | 116 | 4 | High |
| Trueba et al (2000) [344] | Human | / | Ecuador | *F.hepatica* | N | N | N | N | / | N | 150 | 9 | 2 | Middle |
| Turhan et al (2006) [345] | Human | / | Turkey | *F.hepatica* | N | N | N | N | / | N | 597 | 18 | 2 | Middle |
| Ulayi et al (2007) [346] | Cattle | / | Nigeria | *F.gigantica* | N | N | N | N | N | / | 350 | 6 | 3 | Middle |
| Ullah et al (2016) [347] | Cattle | 2013 | Pakistan | *F.hepatica* | Y | Y | N | N | N | / | 70 | 17 | 3 | Middle |
| Valencia et al (2005) [348] | Human | 2004 | Peru | / | Y | N | N | N | / | N | 842 | 33 | 4 | High |
| | Cattle | 2004 | Peru | / | Y | N | N | N | / | N | 532 | 123 | 4 | High |
| Van De et al (2020) [349] | Human | 2018 | Vietnam | / | N | N | Y | Y | / | N | 1120 | 125 | 3 | Middle |
| Villa-Mancera et al (2016) [350] | Sheep | 2014 | Mexico | / | N | N | Y | N | N | / | 1070 | 826 | 3 | Middle |
| Webb et al (2021) [351] | Human | 2013-2018 | Peru | / | N | N | Y | N | / | N | 3000 | 428 | 3 | Middle |
| Wilches et al (2009) [352] | Human | / | Colombia | *F.hepatica* | N | N | N | N | / | N | 61 | 3 | 1 | LOW |
| Yadav et al (2008) [353] | Cattle | 2002-2004 | India | *F.gigantica* | N | N | N | N | N | / | 1091 | 34 | 3 | Middle |
| | Sheep | 2002-2004 | India | *F.gigantica* | N | N | N | N | N | / | 1534 | 21 | 3 | Middle |
| Yadav et al (2009) [354] | Cattle | / | India | *F.gigantica* | N | N | N | N | N | / | 4566 | 384 | 2 | Middle |
| Yasin et al (2018) [355] | Cattle | / | Bangladesh | / | N | N | N | N | N | / | 206 | 4 | 2 | Middle |
| | Sheep | / | Bangladesh | / | N | N | N | N | N | / | 160 | 0 | 2 | Middle |

| | | | | | | | | | | | | | | |
|---|---|---|---|---|---|---|---|---|---|---|---|---|---|---|
| Yatswako and Alhaji (2017) [356] | Cattle | 2004-2014 | Nigeria | / | N | N | N | N | N | / | 3292634 | 47931 | 4 | High |
| Yibar et al (2015) [357] | Cattle | / | Turkey | / | N | N | N | N | | / | 5363 | 0 | 3 | Middle |
| | Sheep | / | Turkey | / | N | N | N | N | | / | 22872 | 0 | 3 | Middle |
| Yilmaz and Gödekmerdan (2004) [358] | Human | / | Turkey | *F.hepatica* | N | N | N | Y | / | N | 500 | 9 | 2 | Middle |
| Yilmaz et al (2007) [359] | Human | / | Turkey | *F.hepatica* | N | N | N | Y | / | N | 867 | 1 | 2 | Middle |
| Zafar et al (2019) [360] | Sheep | / | Pakistan | / | Y | N | N | N | N | / | 384 | 143 | 3 | Middle |
| Zalizar et al (2021) [361] | Cattle | 2019 | Indonesia | / | N | N | N | N | N | / | 100 | 39 | 3 | Middle |
| Zaman et al (2014) [362] | Cattle | 2009-2012 | Pakistan | *F.gigantica* | N | Y | N | Y | N | / | 800 | 61 | 5 | High |
| Zaraei et al (2019) [363] | Cattle | 2018-2019 | Iran | / | N | Y | Y | Y | N | / | 150 | 64 | 4 | High |
| Zewde et al (2019) [364] | Cattle | 2018-2019 | Ethiopia | *Fasciola* | Y | Y | Y | Y | N | / | 247 | 41 | 5 | High |
| Zgabeher et al (2012) [365] | Sheep | 2009-2010 | Ethiopia | / | Y | Y | N | Y | N | / | 384 | 152 | 4 | High |
| Zhang et al (2017) [366] | Cattle | 2013-2014 | China | *F.hepatica* | N | N | Y | Y | N | / | 1584 | 454 | 5 | High |
| Zhang et al (2019) [367] | Cattle | / | China | *F.hepatica* | N | N | N | N | N | / | 767 | 670 | 3 | Middle |
| Zoghi et al (2019) [368] | Human | 2016 | Iran | / | N | N | Y | Y | / | N | 933 | 24 | 3 | Middle |
| Zumaquero-Ríos et al (2013) [369] | Human | / | Mexico | *F.hepatica* | Y | N | Y | N | / | N | 865 | 50 | 3 | Middle |
| / (2010) [370] | Cattle | 2010 | England | / | N | N | N | N | N | / | 969 | 93 | 3 | Middle |
| [371] | Sheep | 2010 | England | / | N | N | N | N | N | / | 1132 | 125 | 3 | Middle |

Footnote: Reference numbers in the Appendix were preceded by "A"; Y, Yes which refers to that there are relevant data in the subgroup; *Fasciola* refers to the data including *F. hepatica* and *F. gigantica*; N, no data; Mic, microscopy; S, slaughter; I, immunological test; MB, molecular biological assay; R, random; NR, not random

1 Abah AE, Wokem GN, Sounyo II. *Fasciola* infection in goats slaughtered from Port Harcourt metropolis Rivers State, Nigeria. Int J One Health. 2019;5: 76-80.

2 Abdi J, Naserifar R, Nejad MR, Mansouri V. New features of fascioliasis in human and animal infections in Ilam province, Western Iran. Gastroenterol Hepatol Bed Bench. 2013;6(3):152-5.

3 Abdulhakim Y, Addis M. An abattoir study on the prevalence of fasciolosis in cattle, sheep and goats in Debre Zeit town, Ethiopia. Glob Vet. 2012; 8(3):308-14.

4 Abdullah I, Tak H, Ganie S A. Prevalence of fasciolosis and dicroceliosis in sheep slaughtered in Kashmir valley. Int J of Sci and Technol Res. 2020;9(1):570-3.

5 Abunna F, Asfaw L, Megersa B, Regassa A. Bovine fasciolosis: coprological, abattoir survey and its economic impact due to liver condemnation at Soddo municipal abattoir, Southern Ethiopia. Trop Anim Health Prod. 2010;42(2):289-92.

6 Acici M, Buyuktanir O, Bolukbas CS, Pekmezci GZ, Gurler AT, Umur S. Serologic detection of antibodies against *Fasciola hepatica* in sheep in the middle Black Sea region of Turkey. J Microbiol Immunol Infect. 2017;50(3):377-81.

7 Addy F, Gyan K, Arhin E, Wassermann M. Prevalence of bovine fasciolosis from the Bolgatanga abattoir, Ghana. Scientific African. 2020; e00469.

8 Adediran OA, Adebiyi AI, Uwalaka EC. Prevalence of *Fasciola* species in ruminants under extensive management system in Ibadan southwestern Nigeria. Afr J Med Med Sci. 2014;43 Suppl:137-41.

9 Adela-Valero M, Victoria-Periago M, Perez-Crespo I, Angles R, Villegas F, Aguirre C, Mas-Coma S. Field evaluation of a coproantigen detection test for fascioliasis diagnosis and surveillance in human hyperendemic areas of Andean countries. PLoS Negl Trop Dis.2012;6(9).

10 Adrien MDL, Schild AL, Marcolongo PC, Fiss L, Ruas JL, Grecco FB, Raffi MB. Acute fasciolosis in cattle in southern Brazil. Pesqui Vet Bras. 2013;33(6):705-9.

11 Afshan K, Fortes-Lima CA, Artigas P, Valero AM, Qayyum M, Mas-Coma S. Impact of climate change and man-made irrigation systems on the transmission risk, long-term trend and seasonality of human and animal fascioliasis in Pakistan. Geospat Health. 2014;8(2):317-34.

12 Afshan K, Kabeer S, Firasat S, Jahan S, Qayyum M. Seroepidemiology of human fascioliasis and its relationship with anti-*Fasciola* IgG and liver enzymes

as biomarkers of pathogenicity. Afr Health Sci. 2020;20(1):208-18.

13 Afshan K, Qayyum M, Rizvi SSR, Mukhtar M, Mushtaq M, Miller J E. Serological and coprological comparison for rapid diagnosis of *Fasciola hepatica* infection in small ruminants from sub-tropical area of Pakistan. Small Rumin Res. 2013;113(1):267-72.

14 Aftab A, Lall R, Bisen S, Anandanarayanan A, Rialch A, Chamuah JK, Raina OK. Serodiagnosis of *Fasciola gigantica* infection in buffaloes with native Cathepsin-L proteases and recombinant Cathepsin L1-D. Acta Parasitol. 2020;65(2):413-21.

15 Agaoglu S, Bicek K, Alemdar S, Deger S. The prevalence of some parasites and enteric pathogens in people working in food sector. Indian Vet J. 2004;81(11):1274-5.

16 Ahmad M, Wan-Nor-Amilah WAW, Kin WW, Arizam MF, Noor-Izani NJ. Prevalence and risk factors of bovine fascioliasis in Kelantan, Malaysia: A cross-sectional study. Trop Life Sci Res. 2021;32(2):1-14.

17 Ahmad I, Yakubu Y, Chafe UM, Bolajoko BM, Muhammad U. Prevalence of fasciolosis (Liver flukes) infection in cattle in Zamfara, Nigeria: A slaughterhouse surveillance data utilizing postmortem examination. Vet. Parasitol. Reg. Stud. Reports 2020;22.

18 Ahmad I, Durrani AZ, Khan MS, Ashraf K, Avais M, Ijaz M, *et al*. Molecular epidemiology of small ruminant fasciolosis in selected regions of Azad and Jammu and Kashmir. J of Anim and Plant Sci. 2017;27(5):1552-9.

19 Ahmad M, Khan MN, Sajid MS, Muhammad G, Qudoos A, Rizwan HM. Prevalence, economic analysis and chemotherapeutic control of small ruminant fasciolosis in the Sargodha district of Punjab, Pakistan. Vet Ital. 2017;53(1):47-53.

20 Ahmed S, Nawaz M, Gul R, Zakir M, Razzaq A. Diversity and prevalence of trematodes in livers of sheep and goat in Quetta, Pakistan. Pakistan J Zool. 2005;37(3):205-10.

21 Akca A, Gokce H I, Mor N. Seroprevalence of *Fasciola hepatica* infection in cattle and sheep in the province of Kars, Turkey, as determined by ELISA. Helminthologia. 2014;51(2):94-7.

22 Akkari H, Gharbi M, Darghouth MA. Dynamics of infestation of tracers lambs by gastrointestinal helminths under a traditional management system in the

north of Tunisia. Parasite. 2012;19(4):407-15.

23 Alawa CBI, Adamu AM, Gefu JO, Ajanusi OJ, Abdu PA, Chiezey NP. In vivo efficacy of vernonia amygdalina (compositae) against natural helminth infection in Bunaji (Bos indicus) calves. Pakistan Vet J. 2010;30(4):215-8.

24 Albuquerque RBdFe, Pereira SA, Melo SNd, Belo VS, Arruda MMd, Mazetto D, *et al*. Spatial distribution analysis of bovine fascioliasis cases recorded in an abattoir in the state of Santa Catarina, Brazil. Cienc Rural. 2022;52(3):e20210030.

25 Ali TS, Zarichehr V, Reza TM, Amroallah B, Hossin T, Amir M, *et al*. Prevalence of liver flukes infections in slaughtered animals in Kashan, Isfahan province, central Iran. IIOAB J. 2011;2(5):14-8.

26 Alsulami MN, Wakid MH, Al-matary M, Abdel-Gaber R, Al-Megrin WAI, Bakhraibah AO, *et al*. Identification and genetic characterization of *Fasciola hepatica* isolated from cattle in Jeddah, Saudi Arabia based on sequence analysis of mitochondrial (COI) gene. Infect. Drug Resist. 2022;15:4877-86.

27 Amer S, ElKhatam A, Zidan S, Feng Y, Xiao L. Identity of *Fasciola* spp. in sheep in Egypt. Parasit Vec. 2016;9.

28 Americo L, Padilha MAC, Arruda PM, Drescher G, de Moura AB, Chryssafidis AL. Epidemiological survey and confirmation of autochthonous cases of bovine fasciolosis in the Serrana Mesoregion of Santa Catarina, Brazil. Frontiers in Vet Sci. 2022;9:933462.

29 Aminzare M, Hashemi M, Faz SY, Raeisi M, Hassanzadazar H. Prevalence of liver flukes infections and hydatidosis in slaughtered sheep and goats in Nishapour, Khorasan Razavi, Iran. Vet World. 2018;11(2):146-50.

30 Amiri S, Shemshadi B, Shirali S, Kheirandish F, Fallahi S. Accurate and rapid detection of *Fasciola hepatica* copro-DNA in sheep using loop-mediated isothermal amplification (LAMP) technique. Vet Med Sci. 2021;7(4):1316-24.

31 Andrade RB, Forero J, Ospina JD. Prevalence of trematodes and host risk factors associated with dairy cattle in Colombia. Rev Vet. 2020;31(2):171-7.

32 Andrade RJ, Tarazona LE, Vargas JC. Prevalence of trematodes and some risk factors in dairy cows in Paipa Boyacá (Colombia). Rev Facultad de Med Vet Zootecnia. 2020;67(3):205-18.

33 Anjum R, Khan MN, Sajid M., Javed MT. Frequency distribution of fasciolosis in small ruminants population at district Sargodha. Glob Vet.

2014;12(1):26-32.

34 Ara I, Ahmed J, Dipta PM, Nath SD, Akter T, Adnan MR, et al. Prevalence and severity of gastro-intestinal parasites in buffalo calves at Sylhet division of Bangladesh. J Parasit Dis. 2021;45(3):620-6.

35 Arafa WM, Hassan AI, Snousi SAM, El-Dakhly KM, Holman PJ, Craig TM, et al. *Fasciola hepatica* infections in cattle and the freshwater snail *Galba truncatula* from Dakhla Oasis, Egypt. J Helmin. 2018;92(1):56-63.

36 Aragaw K, Tilahun H. Coprological study of trematode infections and associated host risk factors in cattle during the dry season in and around Bahir Dar, northwest Ethiopia. Vet Anim Sci. 2019;7:100041.

37 Aragaw K, Negus Y, Denbarga Y, Sheferaw D. Fasciolosis in slaughtered cattle in Addis Ababa abattoir, Ethiopia. Glob Vet. 2012;8(2):115-8.

38 Aram AM. Coprological study of gastrointestinal parasites in dairy cattle in Sulaymaniyah province, Kurdistan region, Iraq. Appl Ecol Environ Res. 2020;18(5):7279-87.

39 Arbabi M, Nezami E, Hooshyar H, Delavari M. Epidemiology and economic loss of fasciolosis and dicrocoeliosis in Arak, Iran. Vet World. 2018;11(12):1648-55.

40 Arias M, Lomba C, Dacal V, Vazquez L, Pedreira J, Francisco I, et al. Prevalence of mixed trematode infections in an abattoir receiving cattle from northern Portugal and north-west Spain. Vet Record. 2011;168(15):408.

41 Arias M, Morrondo P, Hillyer GV, Sanchez-Andrade R, Suarez JL, Lomba C, et al. Immunodiagnosis of current fasciolosis in sheep naturally exposed to *Fasciola hepatica* by using a 2.9 kDa recombinant protein. Vet Parasit. 2007;146(1-2):46-9.

42 Arias C, Lucas JR, Rodríguez A, Córdoba D, Lux-Hoppe EG. Economic impact of the liver condemnation of cattle infected with *Fasciola hepatica* in the Peruvian Andes. Trop Anim Health Prod. 2020;52(4):1927-32.

43 Arroyo MI, Gomez L, Hernandez C, Agudelo D, Galván-Díaz AL, Veldsquez LE. Prevalence of *Fasciola hepatica* and Paramphistomidae in dual purpose cattle in a farm in the Colombian low Andean tropic. Revista de la Facultad de Med Vet y de Zootecnia. 2022;69(1):19-32.

44 Aryaeipour M, Kia EB, Heidari Z, Talaie ZS, Rokni MB. Serological study of human fasciolosis in patients referring to the school of public health, Tehran University of Medical Sciences, Tehran, Iran during 2008-2014. Iranian J Parasit. 2015;10(4):517-22.

45 Asadian S, Mohebali M, Mahmoudi M, Kia EB, Heidari Z, Asgari M, et al. Seroprevalence of human fascioliasis in Meshkin-Shahr district, Ardabil Province, Northwestern Iran in 2012. Iranian J Parasit. 2013;8(4):516-21.

46 Ashraf A, Shahardar RA, Wani ZA, Allaie IM. Prevalence of gastro-intestinal helminths in cattle at north Kashmir. Indian J Anim Sci. 2017;87(2):147-52.

47 Ashrafi K, Saadat F, O'Neill S, Rahmati B, Amin Tahmasbi H, Pius Dalton J, et al. The endemicity of human fascioliasis in Guilan Province, Northern Iran: the baseline for implementation of control strategies. Iran J Public Health. 2015;44(4):501-11.

48 Avcioglu H, Guven E, Balkaya I, Kaynar O, Hayirli A. Evaluation of coprological and serological techniques for diagnosis of bovine fasciolosis. Israel J Vet Med. 2014;69(4):203-10.

49 Ayana D, Waktole H. Study on fasciolosis of sheep and its intermediate host snail in and around Debre-Zeit, East Shoa, Oromia regional state, Ethiopia Glob. Vet. 2013;10(5):571-6.

50 Ayaz MM, Raza MA, Murtaza S, Akhtar S. Epidemiological survey of helminths of goats in southern Punjab, Pakistan. Trop Biomed. 2013;30(1):62-71.

51 Ayaz S, Ullah R, AbdEl NM, Shams S, Niaz S. *Fasciola hepatica* in some buffaloes and cattle by PCR and microscopy. Scientific World J. 2014;462084.

52 Bae P, Hong E, Ryu S, Dong JB., Kim H. Prevalence of Fasciola spp. from cattle in slaughterhouse by macroscopic examination. Korean J Vet Service. 2015;38(4):227-32.

53 Balkaya I, Simsek S. Prevalence and economic importance of hydatidosis and fasciolosis in slaughtered cattle in Erzurum Province of Turkey. Kafkas Univ Vet Fak Derg. 2010;16(5):793-7.

54 Bam J, Deori S, Paul V, Bhattacharya D, Bera AK, Bora L, et al. Seasonal prevalence of parasitic infection of yaks in Arunachal Pradesh, India. Asian Pac J Trop Dis. 2012;2(4):264-7.

55 Barbosa R, Pinto C, Garcia P, Rodrigues A. Prevalence of fasciolosis in slaughtered dairy cattle from Sao Miguel Island, Azores, Portugal. Vet Parasitol Reg

Stud Reports. 2019;17.

56 Barburas DA, Gyorke A, Pop LM, Barburas R, Mircean V, Cozma V. Epidemiology of digestive parasites in buffaloes from Romania. J Hellenic Vet Med Society. 2021;72(4):3329-36.

57 Bellet C, Green MJ., Vickers M, Forbes A, Berry E, Kaler J. *Ostertagia* spp., rumen fluke and liver fluke single- and poly-infections in cattle: An abattoir study of prevalence and production impacts in England and Wales. Prev Vet Med. 2016;132:98-106.

58 Bennema SC, Carvalho Scholte RG, Molento MB, Medeiros C, Carvalho OdS. *Fasciola hepatica* in bovines in Brazil data availability and spatial distribution. Rev Inst Med Trop Sao Paulo. 2014;56(1):35-41.

59 Berhanu D, Tadesse G. Prevalence of Bovine Fasciolosis in selected dairy farms of Addis Ababa, Ethiopia. Momona Ethiopian J Sci. 2018;10(2):282-9.

60 Berhe G, Berhane K, Tadesse G. Prevalence and economic significance of fasciolosis in cattle in Mekelle Area of Ethiopia. Trop Anim Health Prod. 2009;41(7):1503-4.

61 Bernardo C, Carneiro MB, Avelar BR, Donatele DM, Martins IV, Pereira MJ. Prevalence of liver condemnation due to bovine fasciolosis in Southern Espírito Santo: temporal distribution and economic losses. Rev Bras Parasitol Vet. 2011;20(1):49-53.

62 Beyhan YE, Yilmaz H. Seroprevalence of fascioliasis in the eastern region of Turkey: an eight-year investigation. Turk J Gastroenterol. 2020;31(11):746-51.

63 Bhutto B, Arijo A, Phullan, MS, Rind, R. Prevalence of fascioliasis in buffaloes under different agroclimatic areas of sindh province of Pakistan. Int J Agric Biol. 2012;14(2):241-5.

64 Bihaqi SJ, Allaie IM, Banday MAA, Wani ZA, Shahardar RA. Prevalence of caprine GI helminths in temperate areas of Jammu and Kashmir. J Parasit Dis. 2017;41(3):843-9.

65 Birhan M, Demewez G, Fentahun T, Mitiku T. Prevalence and economic significance of bovine fasciolosis in cattle slaughtered at Debre-Tabor municipal abattoir. J Worlds Poult Res. 2019;9(6):246-59.

66 Bitew M, Ibrahim N, Abdela N. Study on the prevalence of ovine fasciolosis in and around Dawa-Cheffa, Kemissie. Afr J Agric Res. 2010;5(21):2981-5.

67 Bless PJ, Schaer F, Khieu V, Kramme S, Muth S, Marti H, et al. High prevalence of large trematode eggs in schoolchildren in Cambodia. Acta Tropica. 2015;141:295-302.

68 Bostanci A, Oguz B. Copro-ELISA Prevalence of *Fasciola hepatica* in cattle in Van, Turkey. Acta Sci Vet. 2017;45.

69 Bozorgomid A, Nazari N, Kia EB, Mohebali M, Hajaran H, Heydarian P, et al. Epidemiology of fascioliasis in Kermanshah Province, Western Iran. Iran J Public Health. 2018;47(7):967-72.

70 Bradbury RS, Arguello I, Lane M, Cooley G, Handali S, Dimitrova S D, et al. Parasitic infection surveillance in Mississippi Delta Children. Am J Trop Med Hyg. 2020;103(3):1150-3.

71 Byrne AW, McBride S, Lahuerta A, Guelbenzu M, McNair J, Skuce RA, et al. Liver fluke (*Fasciola hepatica*) infection in cattle in Northern Ireland: a large-scale epidemiological investigation utilising surveillance data. Parasit Vectors. 2016;9:209.

72 Cabada MM, Goodrich MR, Graham B, Villanueva PG, Deichsel EL, Lopez M, et al. Prevalence of intestinal helminths, anemia, and malnutrition in Paucartambo, Peru. Rev Panam Salud Publica. 2015;37(2):69-75.

73 Cabada MM, Morales ML, Webb CM, Yang L, Bravenec CA, Lopez M, et al. Socioeconomic factors associated with *Fasciola hepatica* infection among children from 26 communities of the Cusco Region of Peru. Am J Trop Med Hyg. 2018;99(5):1180-5.

74 Carlos Pinilla J, Florez Munoz AA, Uribe Delgado N. Prevalence and risk factors associated with liver fluke *Fasciola hepatica* in cattle and sheep in three municipalities in the Colombian Northeastern Mountains. Vet Parasitol Reg Stud Reports. 2020;19.

75 Carneiro MB, Alves DP, Donatele DM, Pereira Júnior OdS, Martins IVF. Fasciola hepatica in sheep, goats and buffaloes in southern municipalities of Espí rito Santo state. Arq Inst Biol. 2013;80(4):442-6.

76 Carnevale S, Cabrera MG, Cucher MA, di Risio CA, Malandrini JB, Kamenetzky L, et al. Direct, immunological and molecular techniques for a fasciolosis survey in a rural area of San Luis, Argentina. J Parasit Dis. 2013;37(2):251-9.

77 Carnevale S, Laura Pantano M, Kamenetzky L, Bruno Malandrini J, Cecilia Soria C, Nestor Velasquez J. Molecular diagnosis of natural fasciolosis by DNA

detection in sheep faeces. Acta Parasitol. 2015;60(2):211-7.

78 Carolina Gonzalez L, Guillermo Esteban J, Dolores Bargues M, Adela Valero M, Ortiz P, Naquira C, et al. Hyperendemic human fascioliasis in Andean valleys: An altitudinal transect analysis in children of Cajamarca province, Peru. Acta Trop. 2011;120(1-2):119-29.

79 Casadiegos M, Alberto Mendoza J. Prevalence and economic losses from livers condemnation infested by Fasciola hepatica at the slaughter plant of Pamplona, North of Santander, Colombia. Rev Cient. 2022;32.

80 Çelik ÖY, Aslan Çelik B. Investigation of the Prevalence of Fasciola hepatica in Small Ruminants in the Siirt Region, Turkey. Iran J Parasitol. 2018;13(4):627-31.

81 Celik OY, Celik BA, Irak K, Akgul G. Assessment of prevalence of Fasciola hepatica and associated biochemical alterations in the cattle of Siirt province, Turkey. Indian J Anim Res. 2019;53(2):260-3.

82 Cengiz ZT, Yilmaz H, Dulger AC, Akdeniz H, Karahocagil MK, Cecek M. Seroprevalence of human fascioliasis in Van province, Turkey. Turk J Gastroenterol. 2015;26(3):259-62.

83 Chakraborty P, Prodhan MAM. Coprological prevalence of bovine fascioliasis, its epidemiology and economic significance in Chittagong district, Bangladesh. Livestock Res Rural Develop. 2015;27(11).

84 Chanie M, Begashaw S. Assessment of the economic impact and prevalence of ovine fasciolosis in Menz Lalo Midir District, Northeast Ethiopia. Vet World. 2012;5(5):261-4.

85 Chaouadi M, Harhoura K, Aissi M, Zait H, Zenia S, Tazerouti F. A post-mortem study of bovine fasciolosis in the Mitidja (north center of Algeria): prevalence, risk factors, and comparison of diagnostic methods. Trop Anim Health Prod. 2019;51(8):2315-21.

86 Chaparro JJ, Ramirez NF, Villar D, Fernandez JA, Londono J, Arbelaez C, et al. Survey of gastrointestinal parasites, liver flukes and lungworm in feces from dairy cattle in the high tropics of Antioquia, Colombia. Parasite Epidemiol Control. 2016;1(2):124-30.

87 Chavez-Arce EV, Zumaran G. Frequency of fascioliasis in cattle and Fasciola hepatica in Lymnaeidae snails in the district of Huanca, Arequipa, Peru. Rev

Investig Vet Peru. 2021;32(3).

88 Chen F, Liu Yh, Yang H, Duan Yc, Yang J, Zhao Yj, et al. Epidemiological investigation on fascioliasis and its risk factors in population of Binchuan County, Yunnan Province. Chin J Schistosomiasis Control. 2015;27(4): 399-402.

89 Choubisa SL, Jaroli VJ. Gastrointestinal parasitic infection in diverse species of domestic ruminants inhabiting tribal rural areas of southern Rajasthan, India. J Parasit Dis. 2013;37(2):271-5.

90 Coelho C, Cruz R, Esteves F, Vala H, Pereira MA, Amorim I, et al. Occurrence and distribution of fasciolosis in a cohort of ovine livestock restricted to a mountain plateau in central Portugal. Anim. 2021;11(12).

91 Cornejo W, Alva P, Sevilla C, Huiza A. Cystatin capture ELISA immunodiagnosis of human fasciolosis at Chupaca-Junin province. An Fac Med. 2003;64(4):252-4.

92 Correa S, Liceth Martinez Y, Lissethe Lopez J, Elena Velasquez L. Evaluation of modified Dennis parasitological technique for diagnosis of bovine fascioliasis. Biomed. 2016;36:64-8.

93 Cuervo PF, Cataldo SD, Fantozzi MC, Deis E, Isenrath GD, Viberti G, et al. Liver fluke (Fasciola hepatica) naturally infecting introduced European brown hare (Lepus europaeus) in northern Patagonia: phenotype, prevalence and potential risk. Acta Parasitol. 2015;60(3):536-43.

94 Cuervo P, Sidoti L, Fantozzi C, Neira G, Gerbeno L, Mera y Sierra R. *Fasciola hepatica* infection and association with gastrointestinal parasites in Creole goats from western Argentina. Rev Bras Parasitol Vet. 2012;22(1):53-7.

95 Curtale F, Hassanein YA, El Wakeel A, Mas S, Montresor A. Distribution of human fascioliasis by age and gender among rural population in the Nile Delta, Egypt. J Trop Pediatr. 2003;49(5):264-8.

96 Da Cunha FOV, Marques SMT, De Mattos MJT. Prevalence of slaughter and liver condemnation due to *Fasciola hepatica* among sheep in the state of Rio Grande do Sul, Brazil 2000 and 2005. Parasitol Latinoam. 2007;62(3-4):188-91.

97 das Chagas Bernardo C, Carneiro MB, de Avelar BR, Donatele DM, Martins IVF, Pereira MJS. Prevalence of liver condemnation due to bovine fasciolosis

in Southern Espírito Santo: Temporal distribution and economic losses. Rev Bras Parasitol Vet. 2011;20(1):49-53.

98 Das M, Deka DK, Sarmah AK, Sarmah PC, Islam S. Gastrointestinal parasitic infections in cattle and swamp buffalo of Guwahati, Assam, India. Indian J Anim Res. 2018;52(12):1732-8.

99 Davoodi L, Mizani A, Najafi R, Teshnizi SH, Amouei A, Motavallihaghi S, et al. A descriptive study of human fascioliasis in Qaemshahr, Mazandaran Province, Iran: Its prevalence and risk factors. Arch Clin Infect Dis. 2022;17(4).

100 de Alegría M, Colmenares K, Espasa M, Amor A, Lopez I, Nindia A, et al. Prevalence of *Strongyloides stercoralis* and other intestinal parasite infections in school children in a rural area of Angola: A cross-sectional study. Am J Trop Med Hyg. 2017;97(4):1226-31.

101 de Aquino FM, Soares VE, Marques Rossi GA, Nicaretta JE, Azeredo Bastos TdS, Cruvinel LB, et al. Prevalence of bovine fascioliasis, areas at risk and ensuing losses in the state of Goias, Brazil. Rev Bras Parasitol Vet. 2018;27(2):123-30.

102 Degheidy NS, Al-Malki JS. Epidemiological studies of fasciolosis in human and animals at Taif, Saudi Arabia. World. Appl Sci J. 2012;19(8):1099-104.

103 Degheidy NS, Al-Malki JS. Incidence and evaluation of anthelmentic efficacy of balanitesegyptiaca on fascioliosis among goats in taif. KSA Glob Vet. 2013;10(1):65-70.

104 Denizhan V, Biçek K. Seroprevalence of *Fasciola hepatica* in sheep in the province of Van and some districts. Ataturk Univ Vet Bilim Derg. 2018;13(3):278-284.

105 Dey AR, Begum N, Anisuzzaman Islam MT, Alam MZ. A large-scale epidemiological investigation on trematode infections in small ruminants in Bangladesh. Vet Med Sci. 2022;8(3):1219-28.

106 Diaz C, Frias H, Cahuana GM, Tapia R, Chenet SM, Tejedo JR. High prevalence and risk factors of fascioliasis in cattle in Amazonas, Peru. Parasitol Int 2021;85.

107 Dixit AK, Das G, Baghel RPS. Gastrointestinal helminthosis: prevalence and associated determinants in goats of Jabalpur, India. J Parasit Dis. 2017;41(2):414-6.

108 Diyana JNA, Lokman IH, Fazila SHN, Latiffah H, Ibitoye EB, Hazfalinda HN, et al. A retrospective study on bovine fascioliasis in veterinary regional laboratories in Peninsular Malaysia. J Parasitol Res. 2019;7903682.

109 Dracz RM, Lima Wdos S. Autochthonous infection of buffaloes and cattle by *Fasciola hepatica* in Minas Gerais, Brazil. Rev Bras Parasitol Vet. 2014;23(3):413-6.

110 El Damaty HM, Mahmmod YS, Gouda SM, Sobhy NM. Epidemiological and ultrasonographic investigation of bovine fascioliasis in smallholder production system in Eastern Nile Delta of Egypt. Prev Vet Med. 2018;158:35-42.

111 Elelu N, Ambali A, Coles GC, Eisler MC. Cross-sectional study of *Fasciola gigantica* and other trematode infections of cattle in Edu Local Government Area, Kwara State, north-central Nigeria. Parasit Vectors. 2016;9.

112 Elliott TP, Kelley JM, Rawlin G, Spithill TW. High prevalence of fasciolosis and evaluation of drug efficacy against Fasciola hepatica in dairy cattle in the Maffra and Bairnsdale districts of Gippsland, Victoria, Australia. Vet Parasitol. 2015;209(1-2):117-24.

113 Elmonir W, Elaadli H, Amer A, El H, Bessat M, Mahmoud SF, et al. Prevalence of intestinal parasitic infections and their associated risk factors among preschool and school children in Egypt. Plos One. 2021;16.

114 Elshahawy IS, Metwally AM, Ibrahim DA. An abattoir-based study on helminthes of slaughtered goats (Capra hircus L., 1758) in upper Egypt, Egypt. Helminth. 2014;51(1):67-72.

115 Elshraway NT, Mahmoud WG. Prevalence of fascioliasis (liver flukes) infection in cattle and buffaloes slaughtered at the municipal abattoir of El-Kharga, Egypt. Vet World. 2017;10(8):914-7.

116 El-Tahawy AS, Kwan N, Sugiura K. *Fasciola hepatica* infection in water buffalo Bubalus bubalis in three provinces of the Nile Delta, Egypt: a cross-sectional study. J Vet Med Sci. 2018;80(1):28-35.

117 El-Tahawy AS, Bazh EK, Khalafalla RE. Epidemiology of bovine fascioliasis in the Nile Delta region of Egypt: Its prevalence, evaluation of risk factors, and its economic significance. Vet World. 2017;10(10):1241-9.

118 Ernest MM, Robertson ID. A retrospective study of the prevalence of bovine fasciolosis at major abattoirs in Botswana. Onderstepoort J Vet Res. 2016;83(1):1-5.

119 Eshrati B, Mokhayeri H, Rokni MB, Kheirandish F, Mafi M, Mokhayeri A, et al. Seroepidemiology of human fascioliasis in rural and nomad areas of Lorestan Province, western Iran, in 2016 and 2017. J Parasit Dis. 2020;44(4):806-12.

120 Esteban JG, Gonzalez C, Curtale F, Munoz C, Valero MA, Bargues MD, et al. Hyperendemic fascioliasis associated with schistosomiasis in villages in the Nile Delta of Egypt. Am J Trop Med Hyg. 2003;69(4):429-37.

121 Ezatpour B, Hasanvand A, Azami M, Anbari K, Ahmadpour F. Prevalence of liver fluke infections in slaughtered animals in Lorestan, Iran. J Parasit Dis. 2015;39(4):725-9.

122 Ezatpour B, Hasanvand A, Azami M, Mahmoudvand H, Anbari K. A slaughterhouse study on prevalence of some helminths of cattle in Lorestan provience, west Iran. Asian Pac J Trop Dis. 2014;4(5):416-20.

123 Feichtenschlager C, Hinney B, Klose S, Tichy A, Tix A, Strobl L, et al. The occurrence of helminths in small ruminants in Styria (Austria) with special attention to the efficacy of benzimidazoles and macrocyclic lactones. Wien Tierarztl Monatsschr. 2014;101(11-12):251-62.

124 Fentie T, Erqou S, Gedefaw M, Desta A. Epidemiology of human fascioliasis and intestinal parasitosis among schoolchildren in Lake Tana Basin, northwest Ethiopia. Trans R Soc Trop Med Hyg. 2013;107(8):480-6.

125 Fernandes Mendes TM, Boas Filho DV, Santo Bataglioli A, do Prado AP, Madi RR, Ueta MT, et al. Bovine fasciolisis in Sao Paulo state, Brazil. Vet Parasitol Reg Stud Reports. 2019;17.

126 Florencia Ojeda N, Gonzalez R, Cornelio S, Alonso Peralta J, Luna C, Machain C, et al. Factors associated with the seizure of livers positive to Fasciola sp. in an endemic area of southeastern Mexico. Rev Mex Cienc Pecu. 2020;11(2):565-75.

127 Forero CG, Anaya AMD, Medellín MOP. Prevalence of Fasciola hepatica in cattle in the slaughterhouse of the municipality of Une, cundinamarca, Colombia.Rev Investig Vet Peru. 2016;27(4):751-7.

128 Founta A, Papadopoulos E, Chliounakis S, Bampidis VA, Papazahariadou M. Presence of endoparasites in the Greek buffalo (Bubalus bubalis) from northern Greece. J. the Hellenic Vet Med Society. 2018;69(2):999-1003.

129 Freites A, Colmenares C, Alarcon-Noya B, Eugenia Garcia M, Diaz-Suarez O. Human fasciolosis in Mara municipality, Zulia state. Venezuela: Prevalence and associated factors. Invest Clin. 2009;50(4):497-506.

130 Fromsa A, Meharenet B, Mekibib B. Major trematode infections of cattle slaughtered at Jimma Municipality abattoir and the occurrence of the intermediate hosts in selected water bodies of the zone. J Anim Vet Adv. 2011;10(12):1592-7.

131 Gao X, Zhang L, Tong X, Zhang H, Mehmood K, Jiang X, et al. Epidemiological survey of fasciolosis in yaks and sheep living on the Qinghai-Tibet plateau, China. Acta Trop. 2020;201.

132 Garg R, Yadav CL, Kumar RR, Banerjee PS, Vatsya S, Godara R. The epidemiology of fasciolosis in ruminants in different geo-climatic regions of north India. Trop Anim Health Prod. 2009;41(8):1695-700.

133 Gauta J, Pérez A, Lecuna J, García M, Aguirre A, Armas S. Prevalence of *Fasciola hepatica* in dairy cattle of Bailadores, State of Mérida, Venezuela. Rev Electron de Vet. 2011;12(12).

134 Gebeyehu EB, Jung BY, Byun JW, Oem JK, Kim HY, Lee SJ, et al. Serologic detection of antibodies against Fasciola hepatica in native Korean goats (Capra hircus coreanae). Vet Med. 2013;58(12):609-12.

135 Gebrecherkos Afera B. Prevalence of bovine fascilosis in municipal Abbatoir of Adigrat, Tigray, Ethiopia Prevalencia de la fasciolosis bovina en el matadero municipal de Adigrat, Tigray, Ethiopia. Rev Electron de Vet. 2012;13(9).

136 Getahun D, van Henten S, Abera A, Senkoro M, Owiti P, Lombamo F, et al. Cysts and parasites in an abattoir in Northwest Ethiopia; an urgent call for action on "one health". J Infect Dev Ctries. 2020;14(6):53S-7S.

137 Ghalandarzadeh B, Amniattalab A. Prevalence and pathological evaluation of hepatic fatty change in cattle slaughtered at urmia abattoir, Northwest Iran. Iraqi J Vet Sci. 2019;33(1):45-50.

138 Gharekhani J, Gerami-Sadeghian A, Yousefi M. Parasitic helminth infections in native sheep (Mehraban) in Hamedan, Iran. J Adv Vet Anim Res. 2015;2(2):115-9.

139 Ghazani MHM, Valilou MR, Ahmadzadeh AR, Karami AR, Zirak K. The prevalence of sheep liver trematodes in the Northwest region of Iran. Turk J Vet Anim Sci. 2008;32(4):305-7.

140 Gherroucha D, Ayadi O, Gharbi M, Benhamza L. Parasitic infection of livers and lungs in cattle and sheep in constantine slaughterhouses, algeria, in 2009-2018. Rev Elev Med Vet Pays Trop. 2021;74(3):177-80.

141 Gherroucha D, Benhamza L, Gharbi M. Prevalence of parasitic lesions in lungs and livers of cattle and sheep at Constantine’s slaughterhouse, Northeast Algeria.Rev Elev Med Vet Pays Trop. 2022;75(1).

142 Gillandt K, Stracke J, Hohnholz T, Waßmuth R, Kemper N. A field study on the prevalence of and risk factors for endoparasites in beef suckler cow herds in Germany. Agriculture (Switzerland). 2018;8(9).

143 Giraldo Pinzón E, Pérez Cárdenas J, Aguilar Marín S, Linares Villalba S. Prevalence of bovine fasciolosis in a zone of caldas colombia with evidences of the disease.Rev UDCA Actual Divulg Cient. 2016;19(1):139-48.

144 Gordon CA, Acosta LP, Gobert GN, Jiz M, Olveda RM, Ross AG, et al. High prevalence of *Schistosoma japonicum* and *Fasciola gigantica* in bovines from northern Samar, the Philippines. PLoS Negl Trop Dis. 2015;9(2).

145 Goz Y, Ay din A, Kilinc OO. The prevalence of liver flukes in sheep slaughtered in Yuksekova, Hakkari Province, eastern Region of Turkey. J Anim Vet Adv. 2014;13(5):287-9.

146 Gupta SC, Ghosh S, Raina OK, Joseph D, Rawat P, Singh BP, et al. Status and prevalence of fasciolosis in cattle and buffaloes in different agro-climatic zones of Uttar Pradesh, India. J Vet Parasitol. 2008;22(2): 59-63.

147 Hamiroune M, Dahmane M, Charef A, Cheniguel H, Foughalia H, Saidani K, et al. Evaluation of fascioliasis, hydatidosis, and tuberculosis in domestic animals during post-mortem inspection at Jijel slaughterhouse (Algeria). J Food Qual Hazards Control. 2020;7(3):149-56.

148 Hammami H, Hamed N, Ayadi A. Epidemiological studies on Fasciola hepatica in Gafsa Oases (south west of Tunisia). Parasite. 2007;14(3):261-4.

149 Haymanot F, Kaba T. Prevalence and associated factors of gastrointestinal helminthiasis of lactating cow and effect of strategic deworming on milk quantity, fat, and protein in Kucha, Ethiopia. BMC. Vet Res. 2022;18(1).

150 Hernandez-Guzman K, Molina-Mendoza P, Olivares J, Alcala Y, Olmedo A, Cordova A, et al. Prevalence and seasonal variation of *Fasciola hepatica* in slaughtered cattle: the role of climate and environmental factors in Mexico. J Helminthol. 2021;95.

151 Höglund J, Dahlström F, Engström A, Hessle A, Jakubek EB, Schnieder T, et al. 2010. Antibodies to major pasture borne helminth infections in bulk-tank milk samples from organic and nearby conventional dairy herds in south-central Sweden. Vet Parasitol. 2010;171(3-4), 293-9.

152 Hosseini G, Sarkari B, Moshfe A, Motazedian MH, Abdolahi Khabisi S. Epidemiology of human fascioliasis and intestinal helminthes in rural areas of Boyer-Ahmad township, southwest Iran; a population based study.Iran J Public Health. 2015;44(11), 1520-5.

153 Ibáñez HN, Jara CC, Guerra MA, Díaz LE. Prevalencia del enteroparasitismo en escolares de comunidades nativas del Alto Marañon, Amazonas, Perú.Rev Peru Med Exp Salud Publica. 2004;21(3), 126-33.

154 Ikenna-Ezeh NH, Eke C, Ezeh IO, Obi CF, Chukwu CC. Prevalence and hematological parameters of *Fasciola gigantica*-infected cattle in Nsukka, Southeastern Nigeria. Comp Clin Path. 2019;28(3), 695-9.

155 Imani Baran A, Cheraghi Saray H, Katiraee F. Molecular determination of *Fasciola* Spp. isolates from domestic ruminants fecal samples in the Northwest of Iran. Iran J Parasitol. 2017;12(2), 243-50.

156 Innocent GT, Gilbert L, Jones EO, McLeod JE, Gunn G, McKendrick IJ, et al. Combining slaughterhouse surveillance data with cattle tracing scheme and environmental data to quantify environmental risk factors for liver fluke in cattle. Front Vet Sci. 2017;4.

157 Isah UM. Studies on the prevalence of fascioliasis among ruminant animals in northern Bauchi state, north-eastern Nigeria. Parasite Epidemiol Control. 2019;5.

158 Jajaa IF, Mushonga B, Green E, Muchenje V. Seasonal prevalence, body condition score and risk factors of bovine fasciolosis in South Africa. Vet Anim

Sci. 2020; 4,9.

159 Japa O, Siriwechviriya P, Prakhammin K. Occurrence of fluke infection in beef cattle around Phayao Lake, Phayao, Thailand. Vet World. 2020;13(2), 334-7.

160 Jean-Richard V, Crump L, Abicho AA, Nare NB, Greter H, Hattendorf J, et al. Prevalence of *fasciola gigantica* infection in slaughtered animals in south-eastern Lake Chad area in relation to husbandry practices and seasonal water levels. BMC Vet Res. 2014;10.

161 Jimenez-Rocha AE, Arguello-Vargas S, Romero-Zuniga JJ, Sequeira-Avalos JA, Dolz G, Montenegro-Hidalgo V, et al. Environmental factors associated with *Dictyocaulus viviparus* and *Fasciola hepatica* prevalence in dairy herds from Costa Rica. Vet Parasitol Reg Stud Reports. 2017;9, 115-21.

162 Kabir HB, Eliyas M, Hashem A, Mohiuddin Miazi OF. Prevalence of zoonotic parasitic diseases of domestic animals in different abattoir of Comilla and Brahman Baria region in Bangladesh. University J Zoology Rajshahi University. 2009;28, 21-5.

163 Kadir MA, Ali NH, Ridha RGM. Prevalence of helminthes, pneumonia and hepatitis in Kirkuk slaughter house, Kirkuk, Iraq. Iraqi J Vet Sci. 2012;26 (suppl. 3), 83-8.

164 Kakar MN, Kakarsulemankhel JK. Prevalence of endo (trematodes) and ecto-parasites in cows and buffaloes of Quetta, Pakistan. Pak Vet J. 2008;28(1), 34-6.

165 Kantzoura V, Kouam MK, Demiris N, Feidas H, Theodoropoulos G. Risk factors and geospatial modelling for the presence of *Fasciola hepatica* infection in sheep and goat farms in the Greek temperate Mediterranean environment. Parasitol. 2011;138(7), 926-38.

166 Kara M, Gicik Y, Sari B, Bulut H, Arslan MO. A slaughterhouse study on prevalence of some helminths of cattle and sheep in Malatya Province, Turkey. J Anim Vet Adv. 2009;8(11), 2200-5.

167 Karahocagil MK, Akdeniz H, Sunnetcioglu M, Cicek M, Mete R, Akman N, et al. A familial outbreak of fascioliasis in Eastern Anatolia: a report with review of literature. Acta Trop. 2011;118(3), 177-83.

168 Karapinar A, Yildirim A, Biskin Z, Duzlu O, Inci A. The investigation of fasciolosis in sheep by coproantigen ELISA and sedimentation-zinc sulphate

flotation technique around Zara Region (Turkey). Kafkas Univ Vet Fak Derg. 2012;18, A7-12.

169 Kato M, Murakami Y, Shimizu M, Yamamoto H, Yonemoto Y, Ishii K, et al. Survey of cattle fascioliasis in Tsuyama Abattoir. Environ Health Prev Med 2005;10(3), 162-5.

170 Katsoulos PD, Christodoulopoulos G, Karatzia MA, Pourliotis K, Minas A. Liver flukes promote cholelithiasis in sheep. Vet Parasitol. 2011;179(1-3), 262-5.

171 Kedir S, Deressa B, Tigre W. Small ruminant fasciolosis in jimma area of south western Ethiopia: Its epidemiology and minimum monetary loss. Glob Vet 2012;9(5), 635-41.

172 Khan W, Rahman H, Rafiq N, Kabir M, Ahmed MS, Escalant PLR. Risk factors associated with intestinal pathogenic parasites in schoolchildren. Saudi J Biol Sci. 2022;29(4), 2782-6.

173 Khan MK, Sajid MS, Khan MN, Iqbal Z, Iqbal MU. Bovine fasciolosis: Prevalence, effects of treatment on productivity and cost benefit analysis in five districts of Punjab, Pakistan. Res. Vet. Sci. 2009;87(1), 70-5.

174 Khan MK, Sajid MS, Khan MN, Iqbal Z, Arshad M, Hussain A. Point prevalence of bovine fascioliasis and the influence of chemotherapy on the milk yield in a lactating bovine population from the district of Toba Tek Singh, Pakistan. J Helminthol. 2011;85(3), 334-8.

175 Khan MN, Sajid MS, Khan MK, Iqbal Z, Hussain A. Gastrointestinal helminthiasis: prevalence and associated determinants in domestic ruminants of district Toba Tek Singh, Punjab, Pakistan. Parasitol Res. 2010;107(4), 787-94.

176 Khanjari A, Bahonar A, Fallah S, Bagheri M, Alizadeh A, Fallah M, et al. Prevalence of fasciolosis and dicrocoeliosis in slaughtered sheep and goats in Amol Abattoir, Mazandaran, northern Iran. Asian Pac J Trop Dis. 2014;4(2), 120-4.

177 Kheder D, Mohamed A. A study on causes of cattle liver condemnation at an abattoir in Omdurman area, Khartoum State, Sudan. BMC Vet Res 2021;17(1).

178 Khedri J, Radfar MH, Nikbakht B, Zahedi R, Hosseini M, Azizzadeh M, et al. Parasitic causes of meat and organs in cattle at four slaughterhouses in

Sistan-Baluchestan Province, Southeastern Iran between 2008 and 2016. Vet Med Sci. 2021;7(4), 1230-6.

179 Kheirandish F, Kayedi MH, Ezatpour B, Anbari K, Karimi Rouzbahani HR, Chegeni Sharafi A, et al. Seroprevalence of human fasciolosis in pirabad, Lorestan Province, Western Iran. Iran J Parasitol. 2016;11(1), 24-9.

180 Khoramian H, Arbabi M, Osqoi MM, Delavari M, Hooshyar H, Asgari M. Prevalence of ruminants fascioliasis and their economic effects in Kashan, center of Iran. Asian Pac J Trop Biomed. 2014;4(11), 918-22.

181 Kim YH, Park JE, Kim JI, Seo EJ. Epidemiological study on prevalence of fascioliasis in slaughtered cattle. Korean J vet Serv. 2008;31(2), 207-17.

182 Kipyegen CK, Muleke CI, Otachi EO. Human and animal fasciolosis: Coprological survey in Narok, Baringo and Kisumu counties, Kenya. Onderstepoort J Vet Res. 2022;89(1).

183 Kiswaga C, Mayenga EL, Silayo FV, Swai ES. Prevalence, causes and economic losses resulting from condemnations in the slaughterhouses/slabs in Eastern and North-eastern zones of Tanzania. Livestock Res Rural Develop. 2016;28(11).

184 Kiziewicz B. Natural infection with *Fasciola hepatica* (Linnaeus, 1758) in the European bison (Bison bonasus) in Bialowieza National Park, Poland. Helmin 2013;50(3), 167-71.

185 Kohi AM, Mehdipoor Y, Ghasemi E, Khaledi M, Madmoli Y. Prevalence of some liver parasites in the slaughtered livestocks in the industrial slaughterhouse of Dezful during 2015. Int J Adv Biotechnol Res. 2017;8(3), 2364-70.

186 Kordshooli MS, Solhjoo K, Armand B, Dowlatkhah H, Jahromi ME. A reducing trend of fasciolosis in slaughtered animals based on abattoir data in South of Iran. Vet World. 2017;10(4), 418-23.

187 Kouadio JN, Evack JG, Achi LY, Fritsche D, Ouattara M, Silue KD, et al. Prevalence and distribution of livestock schistosomiasis and fascioliasis in Cote d'Ivoire: results from a cross-sectional survey. BMC Vet Res 2020;16(1).

188 Kouam MK, Meningue R, Fon DE. Parasitic causes of organ condemnation in cattle slaughtered in Fako abattoirs, South-West region of Cameroon, and estimate of financial losses. J Helminthol. 2019;93(3), 367-71.

189 Kowalczyk SJ, Czopowicz M, Weber CN, Müller E, Kaba J. Accuracy of a diagnostic model based on serum biochemical parameters in detecting cows at an increased risk of chronic fascioliasis. Vet Parasitol. 2018;254, 15-20.

190 Kozlowska-Loj J, Loj-Maczulska A. The prevalence of *Fasciola hepatica* L. infection in cattle in the Lublin province in the years 2009-2012. Annals of Parasite.2013;59(4), 207-8.

191 Kozlowska-Loj J. Prevalence of *Fasciola hepatica* L. infection in cattle in the Lublin province (Poland) in the years 2005-2008. Wiad Parazytol. 2011;57(2), 127-8.

192 Krishna MCM, D'Souza PE. Prevalence of bovine fasciolosis based on faecal examination and abattoir survey in Karnataka. J Parasit Dis. 2015;39(2), 123-5.

193 Kuchai JA, Chishti MZ, Zaki MM, Rasool SAM, Ahmad J, Tak H. Some epidemiological aspects of fascioliasis among cattle of Ladakh. Glob Vet. 2011;7(4), 342-6.

194 Kuerpick B, Conraths FJ, Staubach C, Froehlich A, Schnieder T, StrubeC. Seroprevalence and GIS-supported risk factor analysis of *Fasciola hepatica* infections in dairy herds in Germany. Parasitol. 2013;140(8), 1051-60.

195 Kumar B, Maharana BR, Prasad A, Joseph JP, Patel B, Patel JS. Seasonal incidence of parasitic diseases in bovines of south western Gujarat (Junagadh), India. J Parasit Dis. 2016;40(4), 1342-6.

196 Kurnianto H, Ramanoon SZ, Aziz NAA, Indarjulianto S. Prevalence, risk factors, and infection intensity of fasciolosis in dairy cattle in Boyolali, Indonesia. Vet World. 2022;15(6), 1438-48.

197 Kusumarini SR, Permata FS, Widyaputri T, Prasetyo D. Prevalence of fasciolosis emphasis on age, origin, body condition and post mortem by geographic information systems on sacrificial examination in Malang District - East Java. Paper presented J Physics: Conference Series. 2020.

198 Lambacher B, Ambros C, Karner LM, Hinney B, Schoiswohl J, Tichy A, et al. Trematode and lung worm prevalence in slaughtered lambs in styria. Berl Munch Tierarztl Wochenschr. 2020;133(3-4), 145-8.

199 Latchumikanthan A, Soundararajan C. Serodiagnosis of bovine fasciolosis by enzyme linked immuno sorbent assay in Chennai, Tamil Nadu. Indian J Anim Sci. 2013;83(6), 587-90.

200 Liba JW, Atsanda NN, Francis MI. Economic loss from liver condemnation due to fasciolosis in slaughtered ruminants in Maiduguri abattoir, Borno State, Nigeria. J Adv Vet Anim Res. 2017;4(1):65-70.

201 Lino Zumaquero-Rios J, Sarracent-Perez J, Rojas-Garcia R, Rojas-Rivero L, Martinez-Tovilla Y, Adela Valero M, et al. Fascioliasis and intestinal parasitoses affecting schoolchildren in Atlixco, Puebla State, Mexico: Epidemiology and treatment with nitazoxanide. PLoS Negl Trop Dis. 2013;7(11).

202 Livia-Córdova G, Burga-Cisterna C, Quiroz-Dávila A, Rentería-Samamé B, Mercado-Gamarra A, Del Solar-Vela M, et al. Prevalence and risk factors associated with infection by *Fasciola hepatica* infection in cattle from peasant communities of Huancabamba (Piura-Peru). Rev Investig Vet Peru. 2021;32(1).

203 Lopez M, White AC Jr, Cabada MM. Burden of *Fasciola hepatica* infection among children from Paucartambo in Cusco, Peru. Am J Trop Med Hyg. 2012;86(3):481-5.

204 Lukambagire AH, McHaile DN, Nyindo M. Diagnosis of human fascioliasis in Arusha region, northern Tanzania by microscopy and clinical manifestations in patients. BMC Infect Dis. 2015;15:578.

205 Maciel MG, Lima WDS, de Almeida FLM, Coelho LIARC, Araujo GAN, Lima MG, et al. Cross-sectional serological survey of human fascioliasis in Canutama Municipality in Western Amazon, Brazil. J Parasitol Res. 2018;6823638.

206 Malathi S, Shameem U, Komali M. Prevalence of gastrointestinal helminth parasites in domestic ruminants from Srikakulam district, Andhra Pradesh, India. J Parasit Dis. 2021;45(3):823-30.

207 Manouchehri-Naeini K, Mohammad-Nasiri F, Rokni MB, Kheiri S. Seroprevalence of human fascioliasis in Chaharmahal and Bakhtiyari Province, Southwestern Iran. Iran. J. Public. Health. 2016;45(6);774-80.

208 Mantari TC, Chávez VA, Suárez AF, Arana DC, Pinedo VR, Ccenta ER. Fasciolasis in children of three districts of Junín, Peru. Rev Investig Vet Peru

2012;23(4):454-61.

209 Maqbool, I., Shahardar, R.A., Wani, Z.A., Allaie, I.M., Shah, M.M., 2018. Prevalence of gastrointestinal helminths of cattle in south Kashmir. Indian. J. Anim. Sci. 88(8), 910-4.

210 Marcos L, Maco V, Terashima A, Samalvides F, Espinoza JR, Gotuzzo E. Fascioliasis in relatives of patients with *Fasciola hepatica* infection in Peru. Rev Inst Med Trop Sao Paulo. 2005;47(4):219-22.

211 Marcos L, Romani L, Florencio L, Terashima A, Canales M, Nestares J, et al. Hyperendemic and mesoendemic zones of *Fasciola* infection surrounding urban Lima: an emerging disease? Rev Gastroenterol Peru. 2007;27(1): 31-6.

212 Marcos Raymundo LA, Maco Flores V, Terashima A, Samalvides F, Miranda E, Tantalean M, et al. Hiperendemicidad de fasciolosis humana en el Valle del Mantaro, Perú: Factores de riesgo de la infección por *Fasciola Hepática*. Rev Gastroenterol Peru. 2004;24(2):158-64.

213 Marskole P, Verma Y, Dixit AK, Swamy M. Prevalence and burden of gastrointestinal parasites in cattle and buffaloes in Jabalpur, India. Vet World. 2016;9(11):1214-7.

214 Mas-Coma S, Buchon P, Funatsu IR, Angles R, Artigas P, Valero MA, et al. Sheep and cattle reservoirs in the highest human fascioliasis hyperendemic area: Experimental transmission capacity, field epidemiology, and control within a one health initiative in Bolivia. Front Vet Sci. 2020;7.

215 May K, Bruegemann K, Koenig S, Strube C. Patent infections with *Fasciola hepatica* and paramphistomes (Calicophoron daubneyi) in dairy cows and association of fasciolosis with individual milk production and fertility parameters. Vet Parasitol. 2019;267:32-41.

216 Meguini MN, Righi S, Bouchekhchoukh M, Sedraoui S, Benakhla A. Investigation of flukes (*Fasciola hepatica* and *Paramphistomum* sp.) parasites of cattle in north-eastern Algeria. Ann Parasitol. 2021;67(3):455-64.

217 Meharenet B, Shitu D. Concurrent infection of fascioliasis and trypanosomosis and associated risk factors in local Zebu breed cattle of western Ethiopia. Vet Med Res Reports. 2021;12:15-22.

218 Mellau BL, Nonga HE, Karimuribo ED. Slaughter stock abattoir survey of carcasses and organ/offal condemnations in Arusha region, northern Tanzania.

Trop Anim Health Prod. 2011;43(4):857-64.

219 Mendes TMF, Filho DVB, Bataglioli AS, do Prado AP, Madi RR, Ueta MT, et al. Bovine fasciolisis in São Paulo state, Brazil. Vet Parasitol Reg Stud Reports. 2019;17:100293.

220 Mensur S, Ansuar I, Tesfaye A, Abdulkaf K, Ahmed Y. Small ruminant fasciolosis and its economic impact in an export abattoir of Ethiopia. Livestock Res Rural Develop. 2016;28(9).

221 Mimoune N, Hamiroune M, Boukhechem S, Mecherouk C, Harhoura K, Khelef D, et al. Pathological findings in cattle slaughtered in Northeastern Algeria and associated risk factors. Vet Sci. 2022;9(7).

222 Mochankana ME, Robertson ID. A retrospective study of the prevalence of bovine fasciolosis at major abattoirs in Botswana. Onderstepoort J Vet Res. 2016;83(1).

223 Mochankana ME, Robertson ID. Cross-sectional prevalence of *Fasciola gigantica* infections in beef cattle in Botswana. Trop Anim Health Prod. 2018;50(6):1355-63.

224 Mohamadzadeh T, Shams S, Khanaliha K, Marhamatizadeh MH, Vafa A. A study on prevalence of some helminthic infections of the liver and lungs among ruminants in abattoir of Fars province, Iran. Arch Razi Inst. 2016;71(4): 245-51.

225 Molento MB, Dutra LH, Pritsch IC, Garbin VP, Pereira AM, Gavião A, et al. Fasciola hepatica infection in cattle and the use of simulation models for endemic areas. J Helminthol. 2020;94;e185.

226 Molla D, Nazir S, Mohammed A, Tintagu T. Parasitic infections as major cause of abattoir condemnations in cattle slaughtered at an Ethiopian abattoir: 10-year retrospective study. J Helminthol. 2019;4:e31.

227 Monteiro NKM, de Fontes-Pereira AMA, Castillo R, Fernández O, Fonseca O, Percedo MI. Prevalence of condemned livers and economic losses by *Fasciola* sp. in Huambo, Angola. Revista de Salud Anim. 2013;35(2):9-93.

228 Montiel N, Simoes D, Angulo F, Rojas N, de Chirinos N, Chirinos A. Prevalence of *Fasciola hepatica* in buffalos and its control by albendazoles treatment.

Rev Cient. 2001;11(1):5-11.

229 Moussouni L, Benhanifia M, Saidi M, Ayad A. Prevalence of gastrointestinal parasitism infections in cattle of bass Kabylie area: Case of Bejaia province, Algeria. Macedonian Vet Review. 2018;41(1):73-82.

230 Mulatu H, Addis M. Study on the prevalence and risk factors of fasciolosis in small ruminants in and around Hirna town, Ethiopia. Glob Vet. 2011;7(5):497-501.

231 Mungube EO, Sila DM, Kariuki CW, Bauni SM, Tenhagen BA, Wamae L, et al. A cross-sectional survey on fasciolosis in selected settlements of taveta division, coast province, Kenya. Livestock Res Rural Develop. 2012;24:4.

232 Munyeme M, Munang'andu HM, Nambota A, Muma JB, Phiri AM, Nalubamba KS. The Nexus between bovine tuberculosis and fasciolosis infections in cattle of the Kafue Basin Ecosystem in Zambia: Implications on abattoir surveillance. Vet Med Int. 2012;921869.

233 Murthy GSS, Rao PV. Prevalence of gastro intestinal parasites in ruminants and poultry in Telangana region of Andhra Pradesh. J Parasit Dis. 2014;38(2):190-2.

234 Nahed-Toral J, Lopez-Tirado Q, Mendoza-Martinez G, Aluja-Schunemann A, Trigo-Tavera FJ. Epidemiology of parasitosis in the Tzotzil sheep production system. Small Rumin Res. 2003;49(2):199-206.

235 Najib MA, Noor-Izani NJ, Wan-Nor-Amilah WAW, Wong WK, Faez AM. High seroprevalence of anti-*Fasciola* antibody among cattle farm workers and dwellers in Kelantan. Trop Biomed. 2020;37(2):389-96.

236 Nasreldin N, Zaki RS. Biochemical and immunological investigation of fascioliasis in cattle in Egypt. Vet World. 2020;13(5):923-30.

237 Nath S, Dixit AK, Jaini R, Agrawal V, Dongre S, Das G. Epizootiological studies on gastrointestinal parasites of domestic ruminants in Khandwa district of Madhya Pradesh, India. J Vet Parasitol. 2015;29(1):41-6.

238 Nga Thi N, Thinh Cong L, Minh Duc Co V, Hoang Van C, Ly Thi N, Khanh Thi H, et al. High prevalence of cattle fascioliasis in coastal areas of Thua Thien Hue province, Vietnam. J Vet Med Sci. 2017;79(6):1035-42.

239 Nguyen T, Cheong FW, Liew JW, Lau YL. Seroprevalence of fascioliasis, toxocariasis, strongyloidiasis and cysticercosis in blood samples diagnosed in Medic Medical Center Laboratory, Ho Chi Minh City, Vietnam in 2012. Parasit Vectors. 2016;9(1):486.

240 Nguyen ST, Nguyen DT, Van Nguyen T, Huynh VV, Le DQ, Fukuda Y, et al. Prevalence of *Fasciola* in cattle and of its intermediate host Lymnaea snails in central Vietnam. Trop Anim Health Prod. 2012;44(8):1847-53.

241 Nguyen TG, Le TH, Dao TH, Tran TL, Praet N, Speybroeck N, et al. Bovine fasciolosis in the human fasciolosis hyperendemic Binh Dinh province in Central Vietnam. Acta Trop. 2011;117(1):19-22.

242 Nicoletti MT, Astos EC, Temoche LC, De La Cruz SB. *Fasciola hepatica* prevalence in cattle from the abancay, curahuasi and tamburco districts, Abancay province-apurímac, Peru during the 2018 rainy season. J Trop Pathology 2022;51(2):157-64.

243 Nnabuife HE, Dakul AD, Dogo GI, Egwu OK, Weka PR, Ogo IN, et al. A study on helminthiasis of cattle herds in Kachia grazing reserve (KGR) of Kaduna state, Nigeria. Vet World. 2013;6(11):936-40.

244 Novobilsky A, Novak J, Bjorkman C, Hoglund J. Impact of meteorological and environmental factors on the spatial distribution of *Fasciola hepatica* in beef cattle herds in Sweden. BMC Vet Res. 2015;11.

245 Novobilsky A, Sollenberg S, Hoglund J. Distribution of *Fasciola hepatica* in Swedish dairy cattle and associations with pasture management factors. Geospat Health. 2015;9(2):293-300.

246 Nuñez M, Corrales M, Chirife C, Bejarano C, Presentado G. Prevalence *Fasciola hepatica* and economic losses for bovine liver confiscation in a slaughterhause of the central department, republic of Paraguay. Compen Cien Vet. 2017;7(2):17-21.

247 Nurhidayah N, Satrija F, Retnani EB, Astuti DA, Murtini S. Prevalence and risk factors of trematode infection in swamp buffaloes reared under different agro-climatic conditions in Java Island of Indonesia. Vet World. 2020;13(4):687-94.

248 Nyirenda SS, Sakala M, Moonde L, Kayesa E, Fandamu P, Banda F, et al. Prevalence of bovine fascioliasis and economic impact associated with liver condemnation in abattoirs in Mongu district of Zambia. BMC Vet Res. 2019;15.

249 Obi CF, Akata MC, Ezubelu OJ. Prevalence of gastrointestinal helminth parasites of trade cattle in Aguata and Orumba South Local Government Areas, Southeastern Nigeria. J Parasit Dis. 2020;44(3):546-52.

250 Ojeda-Robertos NF, Peralta-Torres JA, Parra-Bracamonte GM, Cruz-Gonzalez AR, Luna-Palomera C, Ulin-Yzquierdo C, et al. First report and risk of infection of *Fasciola hepatica* (Linnaeus, 1761) in water buffaloes (Bubalus bubalis - Linnaeus, 1758) in Mexico. Vet Parasitol Reg Stud Reports. 2022:28.

251 Okajima J, Shibata K, Takahashi E, Nagafuchi T, Okajima K, Nonaka N. Current status and its epidemiological consideration of *Fasciola* and *Eurytrema* infections in beef cattle of Japan. J Vet Med Sci. 2016;78(5):785-90.

252 Ola-Fadunsin SD, Uwabujo PI, Halleed IN, Richards B. Prevalence and financial loss estimation of parasitic diseases detected in slaughtered cattle in Kwara State, North-central Nigeria. J Parasit Dis. 2020;44(1):1-9.

253 Ola-Fadunsin SD, Ganiyu IA, Rabiu M, Hussain K, Sanda IM, Baba AY, et al. Helminth infections of great concern among cattle in Nigeria: Insight to its prevalence, species diversity, patterns of infections and risk factors. Vet World. 2020;13(2):338-44.

254 Oljira W, Mideksa B, Mekonnen G, Kebebew G, Jorga E. Fasciolosis in sheep and goats slaughtered at abattoirs in Central Ethiopia and associated financial losses. Food Waterborne Parasitol. 2022:28.

255 Opara KN. Population dynamics of *Fasciola gigantica* in cattle slaughtered in Uyo, Nigeria. Trop Anim Health Prod. 2005;37(5):363-8.

256 Opio LG, Abdelfattah EM, Terry J, Odongo S, Okello E. Prevalence of fascioliasis and associated economic losses in cattle slaughtered at Lira Municipality Abattoir in Northern Uganda. Animals. 2021;11(3).

257 Ortiz-Pineda MC, Archila-Barrera OA, Bulla-Castañeda DM, Díaz-Anaya AM, Forero JCG, Garcia-Corredor DJ, et al. Post mortem diagnosis of *Fasciola hepatica* in cattle slaughtered at the Sogamoso, Boyacá processing plant (Boyacá, Colombia). Rev Investig Vet Peru. 2021;32(5).

258 Oryan A, Mansourian M, Moazeni M, Nikahval B, Barband S. Liver distomatosis in cattle, sheep and goats of Northeastern Iran. Glob Vet. 2011;6(3):241-6.

259 Ouchene-Khelifi NA, Ouchene N, Dahmani H, Dahmani A, Sadi M, Douifi M. Fasciolosis due to *Fasciola hepatica* in ruminants in abattoirs and its economic impact in two regions in Algeria. Trop Biomed. 2018;35(1):181-7.

260 Ozturhan H, Emekdas G, Sezgin O, Korkmaz M, Altintas E. Seroepidemiology of *Fasciola hepatica* in Mersin province and surrounding towns and the role of family history of the Fascioliasis in the transmission of the parasite. Turk J Gastroenterol. 2009;20(3):198-203.

261 Palacio Collado D, Bertot Valdes J, Molento MB, Vazquez Gil A, Ortiz Vazquez R, Fortune Napoles C. Economic losses and prevalence of *Fasciola hepatica* in cattle slaughtered in two Cuban provinces. Rev MVZ Cordoba. 2020;25(1).

262 Palacio Collado D, Bertot Valdés JA, Beltrao Molento M, Vázquez Gil Á, Izquierdo Pérez N, Arenal Cruz A, et al. Seasonal behavior of *Fasciola hepatica* in cattle slaughtered at Chacuba Abattoir, Camaguey, Cuba. Rev Producción Animal. 2017:30-5.

263 Paltin AC, Ionita M, Bota A, Ionescu EC, Mitrea IL. A coprological study on gastro-intestinal parasite community in water buffaloes from a romanian farm. AgroLife Sci J. 2020;9(2):200-205.

264 Pandya SS, Hasnani JJ, Patel PV, Chauhan VD, Hirani ND, Shukla R, et al. Study on prevalence of fasciolosis in buffaloes at Anand and Ahmedabad districts, Gujarat. India Vet World. 2015;8(7):870-4.

265 Paz-Silva A, Sanchez-Andrade R, Suarez JL, Pedreira J, Arias M, Lopez C, et al. Prevalence of natural ovine fasciolosis shown by demonstrating the presence of serum circulating antigens. Parasitol Res. 2003;91(4):328-31.

266 Pereira SAE, Freitas CdC, Dutra LV, Molento MB. Correlation between climate data and land altitude for *Fasciola hepatica* infection in cattle in Santa Catarina, Brazil. Rev. Bras. Parasitol Vet. 2020;29(3).

267 Perez CJ, Giraldo-Pinzon EJ, Aguilar-Marín S. First report of human fascioliasis in an endemic region of bovine fascioliasis in Caldas-Colombia. Vector Borne Zoonotic Dis. 2016;16(6):377-81.

268 Perez-Creo A, Bejar JP, Diaz P, Lopez CM, Prieto A, Vina M, et al. *Fasciola hepatica* in sheep from north-western Spain. Risk factor analysis using a capture ELISA (MM3 SERO). Small Rumin Res. 2016;145:103-6.

269 Petersson J, Jokelainen P, Lassen B, Tagel M, Viltrop A, Novobilsky A. Seroprevalence of *Fasciola hepatica* in cattle in Estonia. Vet Parasitol Reg Stud Reports. 2017;10:90-4.

270 Pezeshki A, Aminfar H, Aminzare M. An analysis of common foodborne parasitic zoonoses in slaughtered sheep and cattle in Tehran, Iran, during 2015-2018. Vet World. 2018;11(10):1486-90.

271 Phalee A, Wongsawad C. Prevalence of infection and molecular confirmation by using ITS-2 region of *Fasciola gigantica* found in domestic cattle from Chiang Mai province, Thailand. Asian Pac J Trop Med. 2014;7(3):207-11.

272 Phiri AM, Phiri IK, Sikasunge CS, Monrad J. Prevalence of fasciolosis in Zambian cattle observed at selected abattoirs with emphasis on age, sex and origin. J Vet Med B Infect Dis Vet Public Health. 2005;52(9):414-6.

273 Pilarczyk B, Tomza-Marciniak A, Sablik P, Pilarczyk R. Parasites of the digestive tract in cows managed in alternative (organic and biodynamic) as well as conventional farms in West Pomerania. Ann Parasitol. 2019;65(4):387-96.

274 Pilarczyk B, Tomza-Marciniak A, Pilarczyk R, Bombik E, Seremak B, Udala J, et al. A comparison of the prevalence of the parasites of the digestive tract in goats from organic and conventional farms. Animals. 2021;11(9).

275 Pile E, Gazeta G, Santos J, Coelho B, Serra-Freire N. Occurrence of human fascioliasis in Volta Redonda, Rio de Janeiro, Brazil. Rev Saude Publica. 2000;34(4):413-4.

276 Pinilla JC, Delgado NU, Florez AA. Survey of *Fasciola hepatica* and other gastrointestinal parasitosis in dual-purpose cattle of the Sabana de Torres municipality, Santander, Colombia. Rev Investig Vet Peru. 2019;30(3):1240-8.

277 Pinilla JC, Florez Muñoz AA, Uribe Delgado N. Prevalence and risk factors associated with liver fluke *Fasciola hepatica* in cattle and sheep in three municipalities in the Colombian Northeastern Mountains. Vet Parasitol Reg Stud Reports. 2020;19:100364.

278 Pinilla JC, Flórez P, Sierra MT, Morales E, Sierra R, Vásquez MC, et al. Point prevalence of gastrointestinal parasites in double purpose cattle of Rio de Oro and Aguachica municipalities, Cesar state, Colombia. Vet Parasitol Reg Stud Reports. 2018;12:26-30.

279 Pinilla JC, Florez AA, Orlandoni G, Tobón JC, Ortíz D. Current status of prevalence and risk factors associated with liver fluke *Fasciola hepatica* in cattle raised in different altitudinal regions of Colombia. Vet, Parasitol, Reg, Stud, Reports. 2020;22:100487.

280 Pinilla León JC, Delgado NU, Florez AA. Prevalence of gastrointestinal parasites in cattle and sheep in three municipalities in the Colombian Northeastern Mountain. Vet World. 2019;12(1):48-54.

281 Pino Santos A, Núñez Fernández FA, Martínez Sánchez R, Domenech Cañete I, Rodríguez M, Jeres Puebla L, et al. Prevalence and risk factors for intestinal parasitic infections in a rural community in “consolación del sur” municipality, cuba. West Idian Med J. 2014;63(4):333-9.

282 Piri K, Saidijam M, Maghsood A, Matini M, Fallah M. Prevalence of animal fasciolosis and specification of *Fasciola* spp. Isolated from sheep, goats and cattle by molecular method: Hamadan Province, West of Iran. Iran J Parasitol. 2018;13(4):524-31.

283 Poddar PR, Begum N, Alim MA, Dey AR, Hossain MS, Labony SS. Prevalence of gastrointestinal helminths of sheep in Sherpur, Bangladesh. J Adv Vet Anim Res. 2017;4(3):274-80.

284 Pratiwi KW, Koesdarto S, Harijani, N. Fasciolosis prevalence on several cattle breeds. Indian J Public Health Res Dev. 2019;10(9):1478-1481.

285 Pritsch IC, Augustinhak Stanula EC, dos Anjos A, Bertot JA, Molento MB. Fascioliasis in buffaloes: A 5-year forecast analysis of the disease based on a 15-year survey in Brazil. Rev Bras Parasitol Vet. 2019;28(3):410-5.

286 Pulido-Medellín MO, García-Corredor D, Díaz-Anaya A, Andrade-Becerra R. Screening of gastrointestinal parasites on small sheep exploitations from the municipality of Toca, Colombia. Rev Salud Anim. 2014;36(1):65-9.

287 Quevedo LS, Bruhn FRP, Teixeira JLR, Alberti TS, Scheids HV, Raffi MB, et al. Epidemiological and clinical-pathological aspects of fasciolosis in livers of cattle slaughtered in southern Brazil. Pesqui Agropecu Bras. 2018;39(9):1761-6.

288 Quispe PW, Beltran FM, Vargas MN, Cabanillas AJ, Sanchez RE, Valderrama PA. Hyperendemicity of fascioliasis and risk factors in school-age children from the Orurillo district, Puno. Rev Investig Vet Peru. 2021;32(5).

289 Qureshi AW, Tanveer A, Maqbool A, Niaz S. Seasonal and monthly prevalence pattern of fasciolosis in buffaloes and its relation to some climatic factors in

northeastern areas of Punjab, Pakistan. Iran J Vet Res. 2012;13(2):134-7.

290 Qureshi AW, Tanveer A, Mas-Coma S. Epidemiological analysis of human fascioliasis in northeastern Punjab, Pakistan. Acta Trop. 2016;156:157-64.

291 Qureshi AW, Zeb A, Mansoor A, Hayat A, Mas-Coma S. *Fasciola hepatica* infection in children actively detected in a survey in rural areas of Mardan district, Khyber Pakhtunkhawa province, northern Pakistan. Parasitol Int. 2019;69:39-46.

292 Radfar MH, Nourollahi-Fard SR, Mohammadyari N. Bovine fasciolosis: prevalence, relationship between faecal egg count and worm burden and its economic impact due to liver condemnation at Rudsar abattoir, Northern Iran. J of parasite Dis: official organ of the Indian Society for Parasit. 2015;39(3):522-5.

293 Rashid MM, Hoque MA, Alim MA, Huque KS, Bhuiyan AKFH. Prevalence of gastrointestinal parasites in brahman crossbred cattle of Bangladesh. Livestock Res Rural Develop. 2015;27(7).

294 Rassol AM, Ahmed AM, Sobhy HM, Abdelgayed SS, Hekal SH. Effects of fascioliasis on the economic losses of beef liver at Abu Simbel Abattoir, Aswan Governorate, Egypt. Advanc Anim Vet Sci. 2020;8(11):1175-9.

295 Raymundo LA, Flores VM, Terashima A, Samalvides F, Miranda E, Tantalean M, et al. Hyperendemicity of human fasciolosis in the Mantaro Valley, Peru: factors for infection with *Fasciola hepatica*. Rev gastroenterologia del Peru: organo official Sociedad Gastroenterologia del Peru. 2004;24(2):158-64.

296 Raza MA, Ayaz MM, Nazir MM, Akhtar MS, Aziz M, Murtaza S, et al. Cross-sectional survey of helminthiasis in buffaloes at Tehsil Jatoi and Tehsil Muzaffar Garh, Southern Punjab, Pakistan. Buffalo Bulletin. 2016;35(1):39-48.

297 Rinca KF, Prastowo J, Widodo DP, Nugraheni YR. Trematodiasis occurrence in cattle along the Progo River, Yogyakarta, Indonesia. Vet World. 2019;12(4):593-7.

298 Rita N, Mursyidah AK, Khadijah S. The prevalence of helminthiasis in cattle, Terengganu, Peninsular Malaysia. Trop Biomed. 2017;34(2):324-331.

299 Rizwan HM, Sajid MS, Khan MN, Saqib M. Seroepidemiology of goat fascioliasis in district Sargodha, Punjab, Pakistan based on excretory secretory antigens of the indigenous strains of *Fasciola gigantica*. Rev Med Vet. 2016;167(1-2):10-7.

300 Rizwan HM, Sajid MS, Abbas H, Khan MN, Akram Q, Shamim A. Epidemiology, burden and seasonal variation of fasciolosis determined through faecal examination and excretory/secretory antigens based ELISA. J Hellenic Vet Med Soc. 2021;72(4):3449-54.

301 Rodriguez-Ulloa C, Rivera-Jacinto M, del Valle-Mendoza J, Cerna C, Hoban C, Chilon S, et al. Risk factors for human fascioliasis in schoolchildren in Banos del Inca, Cajamarca, Peru. Trans R Soc Trop Med Hyg. 2018;112(5):216-22.

302 Rojas-Araya D, Montero A, León D, Romero J. Prevalence of fasciolosis in cattle of Costa Rica (2014). Comparison of four techniques for its diagnosis. Rev Vet. 2016;27(2):80-5.

303 Roldan C, Begovoeva M, Lopez-Olvera JR, Velarde R, Cabezon O, Molinar Min AR, et al. Endemic occurrence of *Fasciola hepatica* in an alpine ecosystem, Pyrenees, Northeastern Spain. Transbound Emerg Dis. 2021;68(4):2589-94.

304 Ruhoollah Khan W, Al-Jabr OA, Khan T, Khan A, El-Ghareeb WR, Swelum AA. Prevalence of gastrointestinal parasite in small ruminants of District Dir Upper Khyber Pakhtunkhwa Province of Pakistan. Brazilian J biology. 2021;83:e248978.

305 Ruiz de Alegria MLA, Colmenares K, Espasa M, Amor A, Lopez I, Nindia A, Molina I. Prevalence of *Strongyloides stercoralis* and other intestinal parasite infections in school children in a rural area of Angola: A cross-sectional study. American Am J Trop Med Hyg. 2017;97(4):1226-31.

306 Saad K, Mohammad Faiz M, Nadarasan R, Mursyidah AK, Hassan QN. High prevalence fluke infection at four cattle farms located in Kuala Terengganu, Malaysia. Trop Life Sci Res. 2019;30(3):59-67.

307 Saberinasab M, Mohebali M, Molawi G, Beigom Kia E, Aryaeipour M, Rokni MB. Seroprevalence of human fascioliasis using indirect ELISA in isfahan district, central iran in 2013. Iran J Parasitol. 2014;9(4):461-5.

308 Sahin M, Yilmaz G, Arhan M, Sen I. Hepatic granulomas in Turkey: A 6-year clinicopathological study of 35 cases. Turk. J Gastroenterol. 2014;25(5):524-8.

309 Saltan C, Taskin TG. Prevalence of liver trematode infections in cattle in the province of Agri in Turkey. Turkiye Parazitol Derg. 2020;44(3):132-8.

310 Samakkhah SA, Tooryan F, Hushmandi K, Partovi R. Prevalence of parasitic infections in the liver of slaughtered animals and its economic losses in

modern slaughterhouses of Mazandaran Province during 2018-2019; A retrospective study. J Occupational Health Epidemiology. 2021;10(4):209-16.

311 Sanchez AR, Paz-Silva A, Suarez JL, Panadero R, Pedreira J, Lopez C, Morrondo P. Influence of age and breed on natural bovine fasciolosis in an endemic area (Galicia, NW Spain). Vet Res Commun. 2002;26(5):361-70.

312 Sanchez VMJ, Lewis FI. Investigating the impact of fasciolosis on cattle carcase performance. Vet Parasitol. 2013;193(1-3):307-11.

313 Sarkari B, Ghobakhloo N, Moshfea AA, Eilami O. Seroprevalence of human fasciolosis in a New-Emerging focus of fasciolosis in Yasuj District, Southwest of Iran. Iran J Parasitol. 2012;7(2):15-20.

314 Sayeed MA, Islam B, Nahar N, Bari MS, Sultana S, Arfin S, et al. Epidemiology of livestock and poultry diseases in Jhenaidah district of Bangladesh. Adv Anim Vet Sci. 2020;8(8):804-12.

315 Secchioni E, Sgorbini M, Perrucci S. Gastrointestinal parasites, liver flukes and lungworms in domestic ruminants from central Italy. Large. Anim Rev. 2016;22(5):195-201.

316 Sen M, Yildirim A, Biskin Z, Duzlu O, Inci A. The investigation of fasciolosis in cattle by copro-ELISA and stool examination techniques around the Derinkuyu region. Turkiye Parazitol Derg. 2011;35(2): 81-5.

317 Shahatha SS, Ayyed IM, Mousa MO. An epidemiological and therapeutic study of *Fasciola hepatica* parasite in goats of Anbar Province-Iraq. Paper presented at the IOP Conference Series: Earth and Environmental Science. 2021;904(1):12-24.

318 Shahnawaz M, Shahardar RA, Wani ZA, Shah SA. Prevalence of ovine platyhelminth parasite infections in Ganderbal area of Kashmir valley. Indian J Anim Sci. 2011;81(3):245-8.

319 Shahzad W, Mehmood K, Munir R, Aslam W, Ijaz M, Ahmad R, Sabir AJ. Prevalence and molecular diagnosis of *Fasciola hepatica* in sheep and goats in different districts of Punjab, Pakistan. Pak Vet J. 2012;32(4):535-8.

320 Shamsi L, Samaeinasab S, Samani ST. Prevalence of hydatid cyst, *Fasciola* spp. and *Dicrocoelium dendriticum* in cattle and sheep slaughtered in Sabzevar abattoir, Iran. Ann Parasitol. 2020;66(2):211-6.

321 Shinggu PA, Olufemi OT, Nwuku JA, Baba-Onoja EBT, Iyawa PD. Liver flukes egg infection and associated risk factors in white fulani cattle slaughtered in Wukari, Southern Taraba State, Nigeria. Adv Exp Med. 2019;2671620.

322 Shykat CA, Islam S, Ahmed F, Islam KM, Bhuiyan JU, Nath TC. Current status of fasciolosis of goat in Sylhet, Bangladesh: An integrated morphomolecular study. J Parasitol Res. 2022;6159388.

323 Silvia Paucar S, Amanda Chávez V, Eva Casas A, Fidel Suárez A. Prevalence of fascioliasis and paramphistomiasis in dairy cattle in oxapampa, Pasco. Rev Investig Vet Peru. 2010;21(1):87-92.

324 Singh NK, Singh H, Jyoti Haque M, Rath SS. Prevalence of parasitic infections in cattle of Ludhiana district, Punjab. J Parasite Dis official organ Indian Society Parasitol. 2012;36(2):256-9.

325 Sreedevi C, Hafeez M. Prevalence of gastrointestinal parasites in buffaloes (*bubalus bubalis*) in and around tirupati, India. Buffalo Bull. 2014;33(3):251-5.

326 Sreedhar S, Mohan EM, Babu DS. Prevalence of parasitic infections in cattle and buffaloes of Anantapur district of Andhra Pradesh. Indian J Anim Res. 2009;43(3):230-1.

327 Steinmann P, Usubalieva J, Imanalieva C, Minbaeva G, Stefiuk K, Jeandron A, et al. Rapid appraisal of human intestinal helminth infections among schoolchildren in Osh oblast, Kyrgyzstan. Acta Trop. 2010;116(3):178-84.

328 Sun P, Wronski T, Apio A, Edwards L. A holistic model to assess risk factors of fasciolosis in Ankole cattle. Vet Parasitol Reg Stud Reports. 2020;22:100488.

329 Swai ES, Ulicky E. An evaluation of the economic losses resulting from condemnation of cattle livers and loss of carcass weight due to fasciolosis: A case study from Hai town abattoir, Kilimanjaro region, Tanzania. Livest Res Rural Dev. 2009;21(11).

330 Takang EE, LeBreton M, Ayuk CE, MacLeod ET. A socio-economic study of *Fasciola* infections in cattle and sheep at the Etoudi slaughterhouse, Yaoundé, Cameroon. J Helmin. 2019;94:e92.

331 Takeuchi-Storm N, Denwood M, Hansen TVA, Halasa T, Rattenborg E, Boes J, et al. Farm-level risk factors for *Fasciola hepatica* infection in Danish dairy

cattle as evaluated by two diagnostic methods. Parasit Vectors. 2017;10.

332 Tanabe MB, Caravedo MA, Morales ML, Lopez M, Clinton White, Baca-Turpo B, et al. A comparison of the risk for chronic fascioliasis between children 3 to 5 years and children 6 to 12 years of age in the Cusco Region of Peru. Am J Trop Med Hyg. 2021;105(3):684-7.

333 Taş Cengiz Z, Yılmaz H, Dülger AC, Akdeniz H, Karahocagil MK, Çiçek M. Seroprevalence of human fascioliasis in Van province, Turkey. Turk J Gastroenterol. 2015;26(3):259-62.

334 Tas Cengiz Z, Akbayram S, Cicek M, Yilmaz H. Intestinal parasitoses detected in primary schoolchildren in the Van province. Turkiye. Parazitol Derg. 2009;33(4):289-93.

335 Tavakoli HR, Mahmoodzadeh A, Hajia M. A five years study of fascioliasis and dicrocoeliasis in Iran's slaughterhouses. Asian Pac J Trop Med. 2008;1(4):9-13.

336 Teixeira JN, Barcellos RR, Link DT, Mathias LA, Tobias F, Braga FR, et al. Prevalence of bovine fascioliasis and economic losses in an abattoir located in the state of Espirito Santo, Brazil. Cienc Rura. 2023;53(4).

337 Tembo W, Nonga HE. A survey of the causes of cattle organs and/or carcass condemnation, financial losses and magnitude of foetal wastage at an abattoir in Dodoma, Tanzania. Onderstepoort. J Vet Res. 2015;82(1), 855.

338 Tesfaheywet Z, Negash K. Prevalence of ovine fasciolosis in oda bultum woreda, western hararghe, ethiopia. Glob Vet. 2012;9(5):530-4.

339 Thanasuwan S, Tankrathok A. Comparison of polymerase chain reaction and microscopy for the detection of *Fasciola* spp. in the fecal matter of domestic bovines in Kalasin Province, Thailand. Vet World. 2021;14(11):2878-82.

340 Thapa B, Parajuli RP, Dhakal P. Prevalence and burden of gastrointestinal parasites in stray cattle of the Kathmandu Valley. J Parasitic Dis: official organ of the Indian Society Parasit. 2022;46(3):845-53.

341 Ticona SD, Chávez VA, Casas VG, Chavera CA, Li EO. Prevalence of *Fasciola hepatica* in cattle and sheep of Vilcashuaman, Ayacucho. Rev Investig Vet Peru. 2010;21(2):168-74.

342 Toan N, Cheong FW, Liew JWK, Lau YL. Seroprevalence of fascioliasis, toxocariasis, strongyloidiasis and cysticercosis in blood samples diagnosed in Medic Medical Center Laboratory, Ho Chi Minh City, Vietnam in 2012. Parasit Vectors. 2016;9(1):486.

343 Tongjura JDC, Ombugadu JR, Abdullahi MM, Blessing MA, Amuga GA, Mafuyai HB. Intestinal parasites amongst primary school children attending Ta'al model primary school in Lafia Local Government Area of Nasarawa State, Nigeria. Nigerian J Parasit. 2019;40(1):92-6.

344 Traore SI, Achi LY, Krauth SJ, Sanogo, M, Zinsstag J, Utzinger J, N'Goran EK. Distribution of bovine *Fasciola gigantica* (Cobbold, 1885) in the district des Savanes, northern Cote d'Ivoire. Geospat Health 2021;16(2).

345 Trueba G, Guerrero T, Fornasini M, Casariego I, Zapata S, Ontaneda S, Vasco L. Detection of *Fasciola hepatica* infection in a community located in the Ecuadorian Andes. Am J Trop Med Hyg. 2000; 62(4), 518.

346 Turhan O, Korkmaz M, Saba R, Kabaaaliogu A, Inan D, Mamikoglu L. Seroepidemiology of fascioliasis in the Antalya region and uselessness of eosinophil count as a surrogate marker and portable ultrasonography for epidemiological surveillance. Infez Med. 2006;14(4):208-12.

347 Ulayi BM, Umaru-Sule B, Adamu S. Prevalence of *Dicrocoelium hospes* and *Fasciola gigantica* infections in cattle at slaughter in Zaria, Nigeria. J Anim Vet Adv. 2007;6(9):1112-15.

348 Ullah I, Nisar MF, Khan Jadoon AA, Tabassum S. Prevalence of *Fasciola hepatica* in domesticated cattle of district Karak, Khyber Pakhtunkhwa, Pakistan. Asian J Anim Sci. 2016;10(1):85-91.

349 Valencia MN, Pariona DA, Huamán AM, Miranda MF, Quintanilla CS, Gonzáles AA. Seroprevalencia de fasciolosis en escolares y en ganado vacuno en la provincia de huancavelica, Perú Rev Peru Med Exp Salud Publica. 2005;22(2):96-102.

350 Van De N, Minh PN, Bich NN, Chai JY. Seroprevalence of tissue and luminal helminths among patients in hanoi medical university hospital, Vietnam, 2018. Korean J Parasitol. 2020;58(4):387-92.

351 Villa-Mancera A, Molina-Mendoza P, Hernandez-Guzman K, Olivares-Perez J, Sarracent-Perez J, Zumaquero-Rios J. Comparative diagnosis of serum IgG1 and coproantigen ELISA for fasciolosis detection of goats in Mexico. BioMed Res Int. 2016.

352 Webb CM, Morales ML, Lopez M, Baca-Turpo B, Arque E, White AC, et al. Stunting in pre-school and school-age children in the Peruvian highlands and its association with *Fasciola* infection and demographic factors. PLoS Negl Trop Dis. 2021;15(6):e0009519.

353 Wilches C, Jaramillo JG, Muñoz DL, Robledo SM, Vélez I D. Presence of infection by *Fasciola hepatica* in inhabitants from the valle de San Nicolás, eastern Antioquia. Infectio. 2009;13(2):92-9.

354 Yadav CL, Garg R, Kumar RR, Vatsya S. Prevalence of fasciolosis in ruminants in national capital region, Delhi. Indian. J. Anim. Sci. 2008;78(3): 269-71.

355 Yadav CL, Garg R, Banerjee PS, Kumar RR, Kumar S. Epizootiology of *Fasciola gigantica* infection in cattle and buffaloes in Western Uttar Pradesh, India. J Vet Parasitol. 2009;23(2):135-8.

356 Yasin MG, Alim MA, Anisuzzaman, Ahasan SA, Munsi MN, Chowdhury EH, et al. Trematode infections in farm animals and their vector snails in Saint Martin's Island, the southeastern offshore area of Bangladesh in the Bay of Bengal. J Vet Med Sci. 2018;80(4): 684-8.

357 Yatswako S, Alhaji NB. Survey of bovine fasciolosis burdens in trade cattle slaughtered at abattoirs in North-central Nigeria: The associated predisposing factors and economic implication. Parasite Epidemiol Control 2(2). 2017;30-9.

358 Yibar A, Selcuk O, Senlik B. Major causes of organ/carcass condemnation and financial loss estimation in animals slaughtered at two abattoirs in Bursa Province, Turkey. Prev Vet Med. 2015;118(1):28-35.

359 Yilmaz H, Arabaci F, Ozdal N, Tas Z, Metin S, Orunc O. The prevalence of intestinal parasite infections among schoolchildren of Van province, Turkey. Trop Doct. 2007;37(2):123-4.

360 Yilmaz H, Gödekmerdan A. Human fasciolosis in Van province, Turkey. Acta Trop. 2004;92(2):161-2.

361 Zafar A, Khan MK, Sindhu ZUD, Abbas RZ, Masood S, Abbas, Z, et al. Seroprevalence of *Fasciola hepatica* in small ruminants of District Chakwal, Punjab, Pakistan. Pak Vet J. 2019;39(1):96-100.

362 Zalizar L, Rahmawati K, Yaro A. Fasciolosis infection level of various breed cattle in Batu and Pujon district, East Java, Indonesia. 1st International Conference on Bioenergy and Environment. Sustain Agri Tech EDP Sci. 2021.

363 Zaman MA, Sajid M, Sikandar A, Tauseefur R, Awais MM. Point prevalence of gastrointestinal helminths and their association with sex and age of buffaloes in lower Punjab, Pakistan. Int J Agric Biol. 2014;16(6):1229-31.

364 Zaraei M, Arefkhah N, Moshfe A, Ghorbani F, Mikaeili F, Sarkari B. Prevalence of bovine fascioliasis in a new-emerging focus of human fascioliasis in BoyerAhmad district, southwest of Iran.Prevalence of bovine fascioliasis in a new-emerging focus of human fascioliasis in BoyerAhmad district, southwest of Iran. Comp Immunol Microbiol Infect Dis. 2019;66.

365 Zewde A, Bayu Y, Wondimu A. Prevalence of bovine fasciolosis and its economic loss due to liver condemnation at Wolaita Sodo Municipal Abattair, Ethiopia. Vet Med International. 2019; 9572373.

366 Zgabeher EG, Amede Y, Bekele M. Prevalence of ovine fasciolosis in Adigrat, north east Ethiopia. Glob Vet. 2012;9(1): 92-6.

367 Zhang XX, Feng SY, Ma JG, Zheng WB, Yin MY, Qin SY, et al. Seroprevalence and risk factors of fascioliasis in yaks, Bos grunniens, from three counties of Gansu Province, China. Korean J Parasitol. 2017;55(1):89-93.

368 Zhang JL, Si HF, Zhou XZ, Shang XF, Li B, Zhang JY. High prevalence of fasciolosis and evaluation of the efficacy of anthelmintics against *Fasciola hepatica* in buffaloes in Guangxi, China. Int J Parasitol Parasites Wildl. 2019;8: 82-7.

369 Zoghi S, Emami M, Shahriarirad S, Vahedi R, Cheraghi MR, Zamiri B, et al. Human fascioliasis in nomads: A population-based serosurvey in southwest Iran. Infez Med. 2019;27(1): 68-72.

370 Zumaquero-Ríos JL, Sarracent-Pérez J, Rojas-García R, Rojas-Rivero L, Martínez-Tovilla Y, Valero MA, et al. Fascioliasis and intestinal parasitoses affecting schoolchildren in Atlixco, Puebla State, Mexico: epidemiology and treatment with nitazoxanide. PLoS Negl Trop Dis. 2013;7(11): e2553.

371 Northern Ireland disease surveillance, July to September 2010. Vet. Rec. 2010; 167(24):924-7.

A

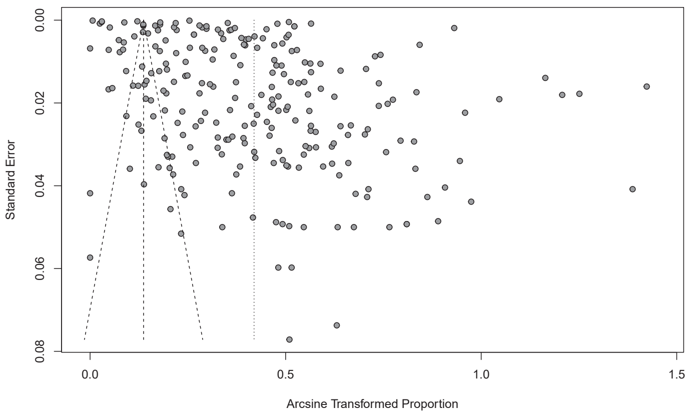

Standard Error
0.00
0.02
0.04
0.06
0.08
0.0
0.5
1.0
1.5
Arcsine Transformed Proportion


B

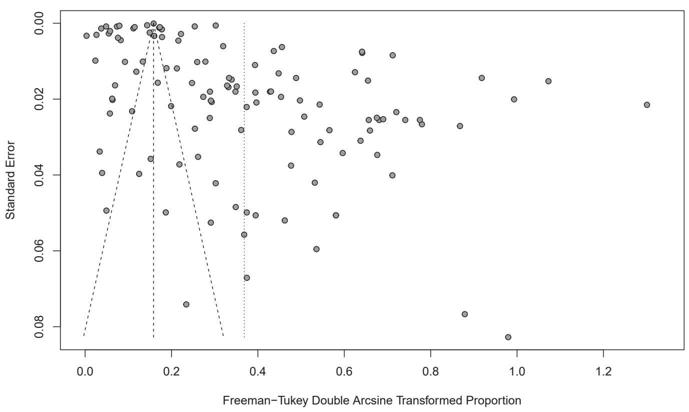

Standard Error
0.00
0.02
0.04
0.06
0.08
0.0
0.2
0.4
0.6
0.8
1.0
1.2
Freeman−Tukey Double Arcsine Transformed Proportion


C

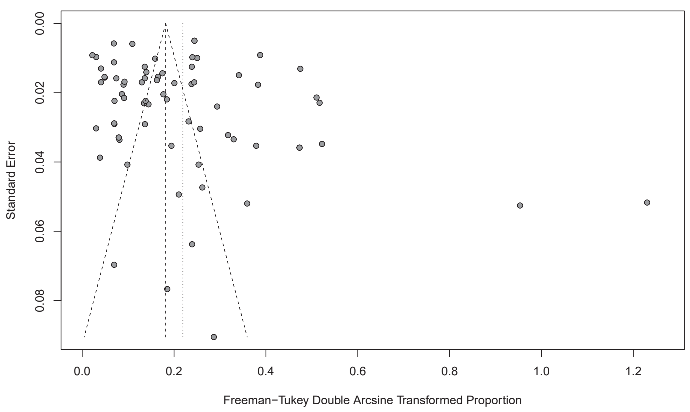

Standard Error
0.00
0.02
0.04
0.06
0.08
0.0
0.2
0.4
0.6
0.8
1.0
1.2
Freeman−Tukey Double Arcsine Transformed Proportion

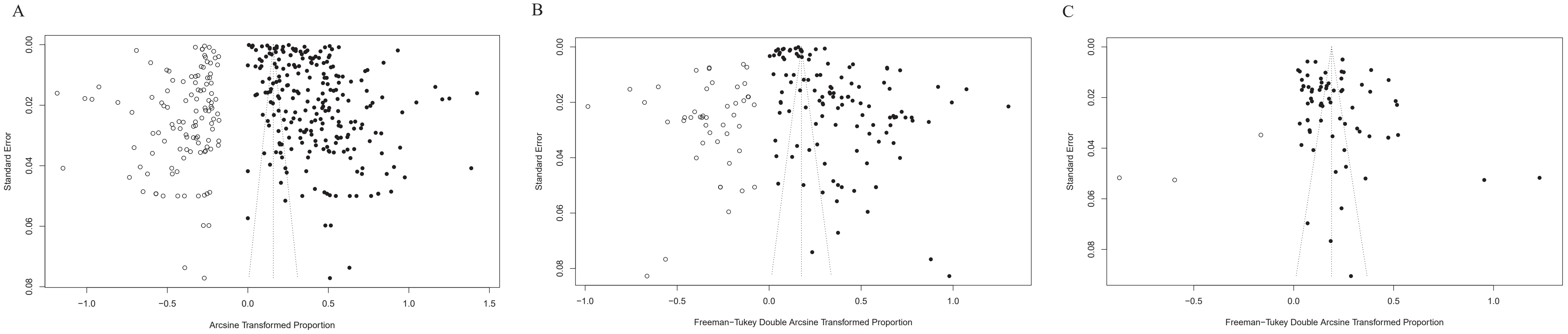
A
Standard Error
Arcsine Transformed Proportion
B
Standard Error
Freeman−Tukey Double Arcsine Transformed Proportion
C
Standard Error
Freeman−Tukey Double Arcsine Transformed Proportion

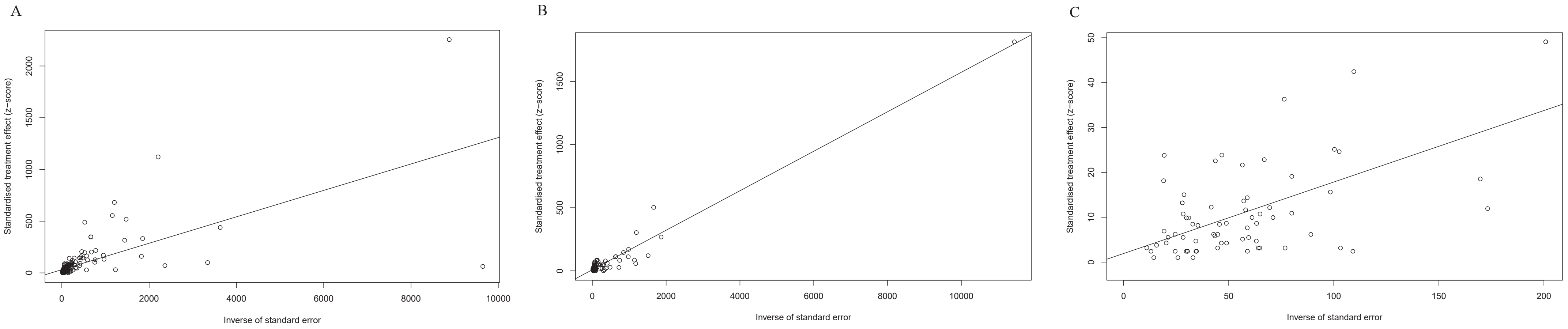
A
Standardised treatment effect (z−score)
0
500
1000
1500
2000
Inverse of standard error
0
2000
4000
6000
8000
10000
B
Standardised treatment effect (z−score)
0
500
1000
1500
Inverse of standard error
0
2000
4000
6000
8000
10000
C
Standardised treatment effect (z−score)
0
10
20
30
40
50
Inverse of standard error
0
50
100
150
200

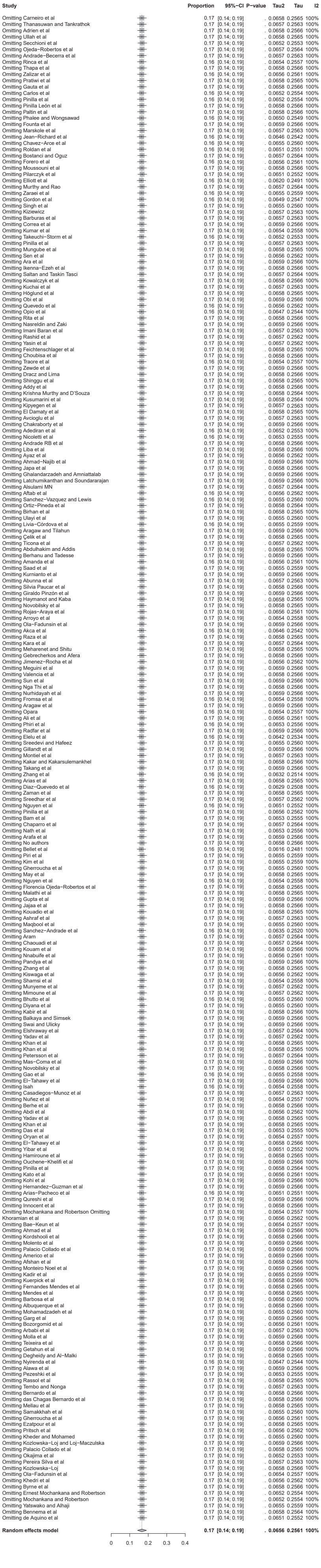
Study
Proportion
95%−CI
P−value
Tau2
Tau
I2
Random effects model
0.17 [0.14; 0.19] . 0.0656 0.2561 100%
0
0.1
0.2
0.3
0.4

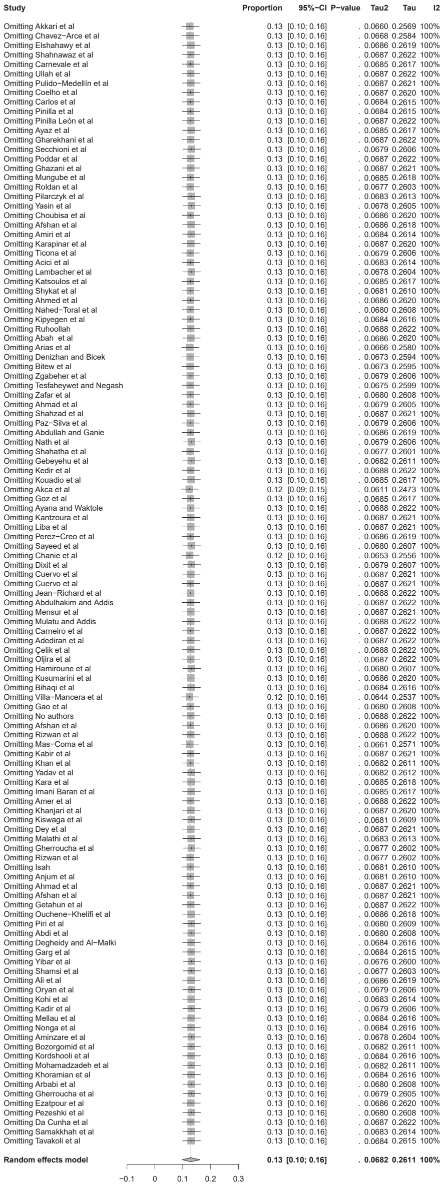
Study
Proportion
95%−CI
P−value
Tau2
Tau
I2
Random effects model
0.13 [0.10; 0.16] . 0.0682 0.2611 100%
−0.1
0
0.1
0.2
0.3

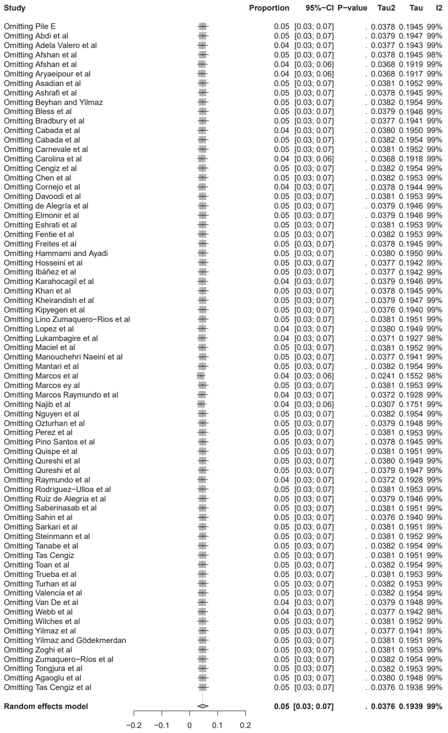
Study
Proportion
95%−CI
P−value
Tau2
Tau
I2
Random effects model
0.05 [0.03; 0.07] . 0.0376 0.1939 99%
−0.2
−0.1
0
0.1
0.2
